# C2ORF68 stabilizes SIVA1 to upregulate BCL-2 and suppress apoptosis in gallbladder cancer

**DOI:** 10.1038/s41420-026-03279-5

**Published:** 2026-07-30

**Authors:** Xuanjia Dong, Lingxiao Zhang, Wenjun Dong, Qiuyi Tang, Jingwei Zhao, Zhaohui Jin, Lin Jiang, Wenting Dai, Yangyang Zhai, Ying Huang

**Affiliations:** Shanghai Key Laboratory of Biliary Tract Disease Research, Department of General Surgery, Xinhua Hospital, Shanghai Jiao Tong University School of Medicine, Shanghai, 200092 China

**Keywords:** Gall bladder cancer, Apoptosis

## Abstract

Gallbladder cancer (GBC) is the most aggressive malignancy of the biliary tract, and its molecular drivers remain insufficiently understood. SIVA1 (apoptosis regulatory protein Siva 1) has been implicated in the regulation of cell survival, but its function in GBC has not been defined. In this study, we found that SIVA1 expression was elevated in GBC tissues and cell lines, and high SIVA1 expression was associated with poor patient prognosis. Gain- and loss-of-function experiments demonstrated that SIVA1 promoted GBC cell proliferation and migration in vitro and facilitated tumor growth in nude mice xenograft model. Mechanistically, SIVA1 was subject to ubiquitin-proteasome-mediated degradation, and we identified C2ORF68 as a binding partner that reduced SIVA1 ubiquitination and stabilized its protein level. Stabilized SIVA1 in turn increased the expression of the anti-apoptotic protein BCL-2, thereby enhancing cell survival. These findings indicate that C2ORF68-mediated stabilization of SIVA1 promotes GBC progression through BCL-2-dependent anti-apoptotic signaling, supporting SIVA1 as a potential therapeutic target.

## Introduction

Gallbladder cancer (GBC) is a rare but highly lethal malignancy of the biliary tract that most commonly arises in the gallbladder fundus, body, and cystic duct. Due to its insidious onset and lack of early symptoms, GBC is frequently misdiagnosed as benign gallbladder disease, and the majority of patients present with advanced-stage tumors at diagnosis [1]. The 5-year overall survival rate remains approximately 5% [2–4]. Adenocarcinoma is the predominant histological subtype, accounting for ~82% of cases [5]. GBC is characterized by strong invasiveness, rapid lymphatic and hematogenous dissemination, and high rates of metastasis to the liver, lungs, and peritoneum [6]. Moreover, most patients respond poorly to conventional chemotherapy and radiotherapy and frequently develop drug resistance [7, 8]. These clinical challenges highlight the urgent need to identify effective therapeutic targets and clarify the molecular mechanisms driving GBC progression.

SIVA1 (apoptosis regulatory protein Siva 1) was initially identified through a yeast two-hybrid screen for proteins interacting with the cytoplasmic tail of CD27, a member of the tumor necrosis factor receptor family [9]. Two transcripts encode SIVA isoforms, among which the primary transcript consists of four exons encoding a 175-amino-acid protein [10]. The shorter isoform, SIVA2, which lacks exon 2, may differ functionally from SIVA1 [11, 12]. SIVA1 is primarily localized to the nucleus as well as mitochondria and cytoplasm, with its subcellular localization being critical for its functional activities [13].

Under physiological conditions, SIVA1 is transcriptionally regulated by p53 [14] and acts as a pro-apoptotic protein [15, 16]. It interacts with BCL-xL via its SAH domain to suppress anti-apoptotic function and enhance apoptosis [17], and similarly sensitizes breast cancer cells to cisplatin by modulating BCL-2 signaling [18]. However, accumulating evidence indicates that SIVA1 may also promote tumor progression in a context-dependent manner [19–21], acting as an oncogenic factor in NSCLC models [22], while suppressing migration in breast cancer cells [23]. These findings suggest that the functional role of SIVA1 is highly cell-type- and pathway-specific.

However, the role of SIVA1 in GBC has remained unclear. We show that SIVA1 is upregulated in GBC and promotes tumor cell proliferation and survival. We identify C2ORF68 as a SIVA1-binding protein that stabilizes SIVA1 by preventing its ubiquitin-proteasome-mediated degradation. Stabilized SIVA1 increases BCL-2 protein levels and suppresses apoptosis. These findings demonstrate that C2ORF68 maintains SIVA1 protein stability to enhance BCL-2-dependent survival signaling in GBC, and support SIVA1 as a potential therapeutic target.

## Results

### SIVA1 is upregulated in gallbladder cancer and predicts poor prognosis

**Fig. 1:**
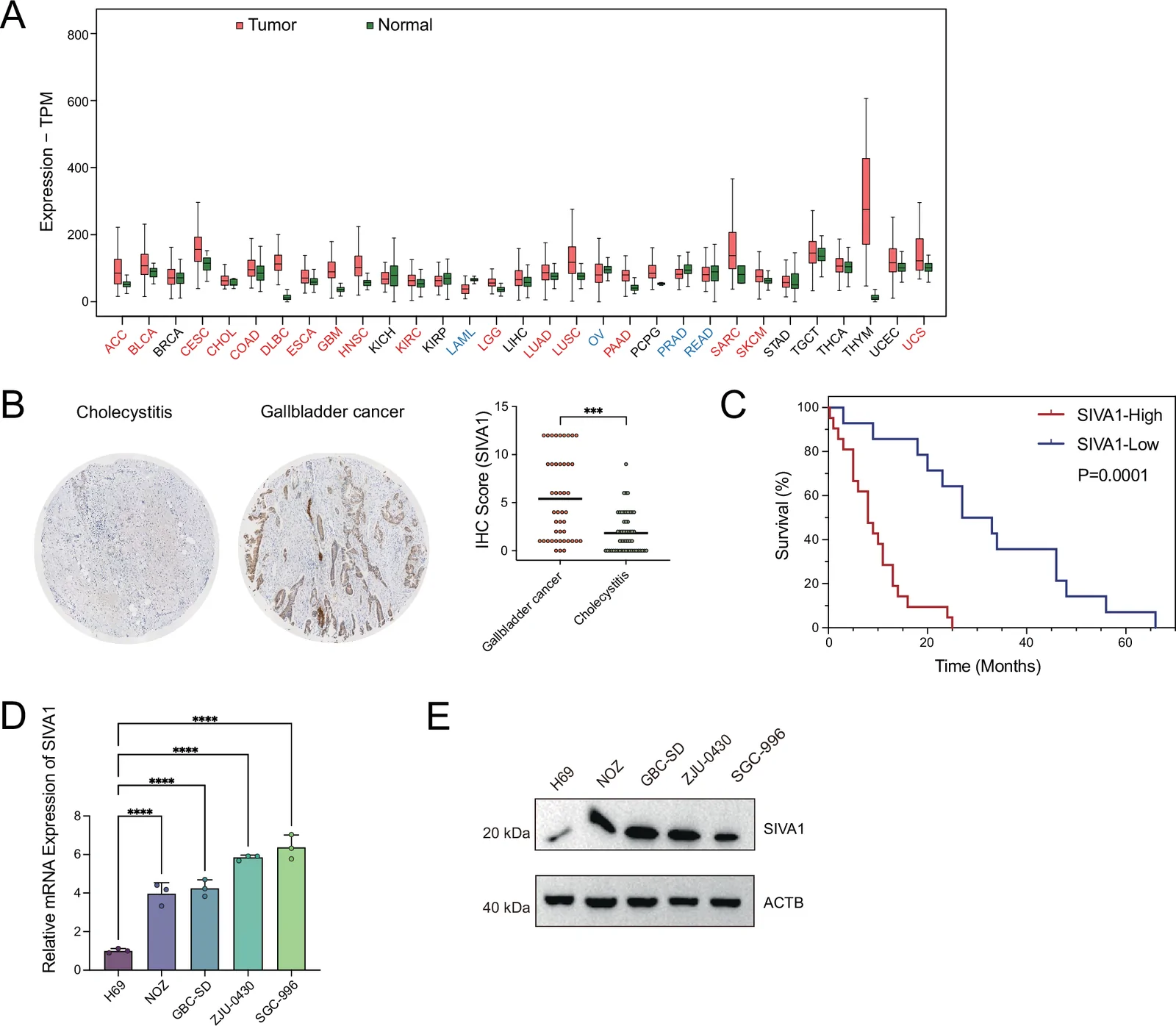
SIVA1 is upregulated in gallbladder cancer and correlates with poor prognosis. **A** SIVA1 expression in tumor and normal tissues based on TCGA and GTEx databases. **B** Representative IHC staining of SIVA1 in GBC and cholecystitis tissues (left), and quantification of IHC scores (right). ***P < 0.001. **C** Kaplan-Meier overall survival analysis of GBC patients stratified by SIVA1 expression (*n* = 47). qRT-PCR (**D**) and Western blotting (**E**) analysis of SIVA1 expression in the nonmalignant biliary epithelial cell line H69 and GBC cell lines. Data information: The experiments were repeated three times and data shown as the mean ± SD.

To investigate the clinical significance of SIVA1 in gallbladder cancer (GBC), we first analyzed its expression profile using the GEPIA database. SIVA1 mRNA expression was increased in several digestive system malignancies, including cholangiocarcinoma (CHOL) and pancreatic adenocarcinoma (PAAD), compared to corresponding normal tissues (Fig. 1A). These initial observations suggested that SIVA1 dysregulation may contribute to carcinogenesis within the hepatobiliary system. We next examined SIVA1 protein levels in a gallbladder cancer tissue microarray (TMA) consisting of 45 GBC specimens and 53 chronic cholecystitis samples. Immunohistochemical (IHC) staining revealed robust cytoplasmic and perinuclear SIVA1 expression in tumor cells, while cholecystitis tissues displayed consistently lower or absent staining (Fig. 1B). Quantification of staining intensity confirmed a substantial elevation of SIVA1 expression levels in GBC tissues. To evaluate its prognostic relevance, overall survival analysis was performed in an independent cohort of 47 GBC patients. Kaplan-Meier analysis demonstrated that high SIVA1 expression correlated strongly with reduced overall survival (P = 0.0001; Fig. 1C). These findings indicate that SIVA1 is upregulated in GBC and may serve as an unfavorable prognostic biomarker.

To further confirm these results, we analyzed SIVA1 expression in multiple GBC cell lines, including NOZ, GBC-SD, ZJU-0430, and SGC-996, alongside the nonmalignant biliary epithelial cell line H69. qRT-PCR and Western blotting consistently demonstrated markedly elevated SIVA1 expression in all four GBC cell lines relative to H69 (Fig. 1D, E). Collectively, these data support that SIVA1 upregulation is a common molecular feature of GBC and is associated with poor clinical outcomes.

### SIVA1 promotes the proliferation and migration of gallbladder cancer cells in vitro

**Fig. 2:**
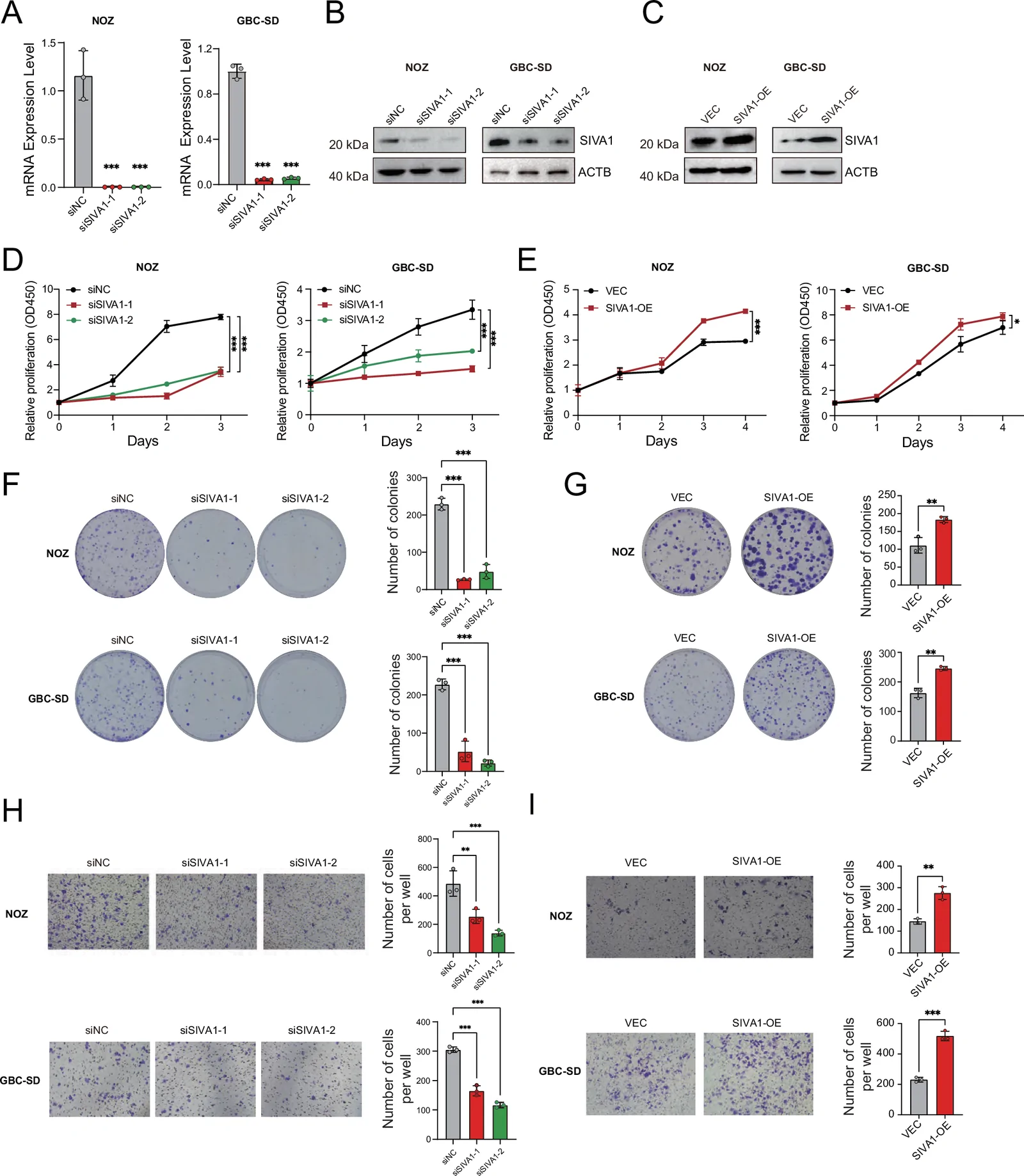
SIVA1 regulates gallbladder cancer cell proliferation and migration in vitro. qRT-PCR (**A**) and Western blotting (**B**) confirming SIVA1 knockdown in NOZ and GBC-SD cells. **C** Western blotting confirming SIVA1 overexpression in NOZ and GBC-SD cells. CCK-8 assays showing reduced proliferation after SIVA1 knockdown (**D**) and increased proliferation after SIVA1 overexpression (**E**). Colony formation assays showing decreased colony number and size upon SIVA1 knockdown (**F**) and increased colony formation upon SIVA1 overexpression (**G**). Transwell migration assays showing impaired migration following SIVA1 knockdown (**H**) and enhanced migration following SIVA1 overexpression (**I**). Data information: The experiments were repeated three times and data shown as the mean ± SD.

To determine the functional role of SIVA1 in GBC progression, both gain- and loss-of-function approaches were employed in NOZ and GBC-SD cells. Two siRNAs (siSIVA1-1 and siSIVA1-2) effectively reduced endogenous SIVA1 expression, while transient transfection with pCMV-SIVA1 resulted in marked SIVA1 upregulation at both mRNA and protein levels (Fig. 2A–C).

Cell proliferation assays revealed that SIVA1 overexpression led to enhanced cell growth in both cell lines, whereas SIVA1 knockdown suppressed proliferation (Fig. 2D, E). Colony formation assays further supported these findings: SIVA1 overexpression increased both colony number and size, whereas knockdown led to substantial reductions in clonogenic capacity (Fig. 2F, G). Furthermore, Transwell assays demonstrated that SIVA1 overexpression promoted cell migration, while SIVA1 depletion dramatically impaired migratory ability (Fig. 2H, I). Together, these results indicate that SIVA1 functions as an oncogenic driver that promotes proliferation and metastatic potential in GBC cells.

### SIVA1 knockdown suppresses tumorigenicity of gallbladder cancer cells in vivo

**Fig. 3:**
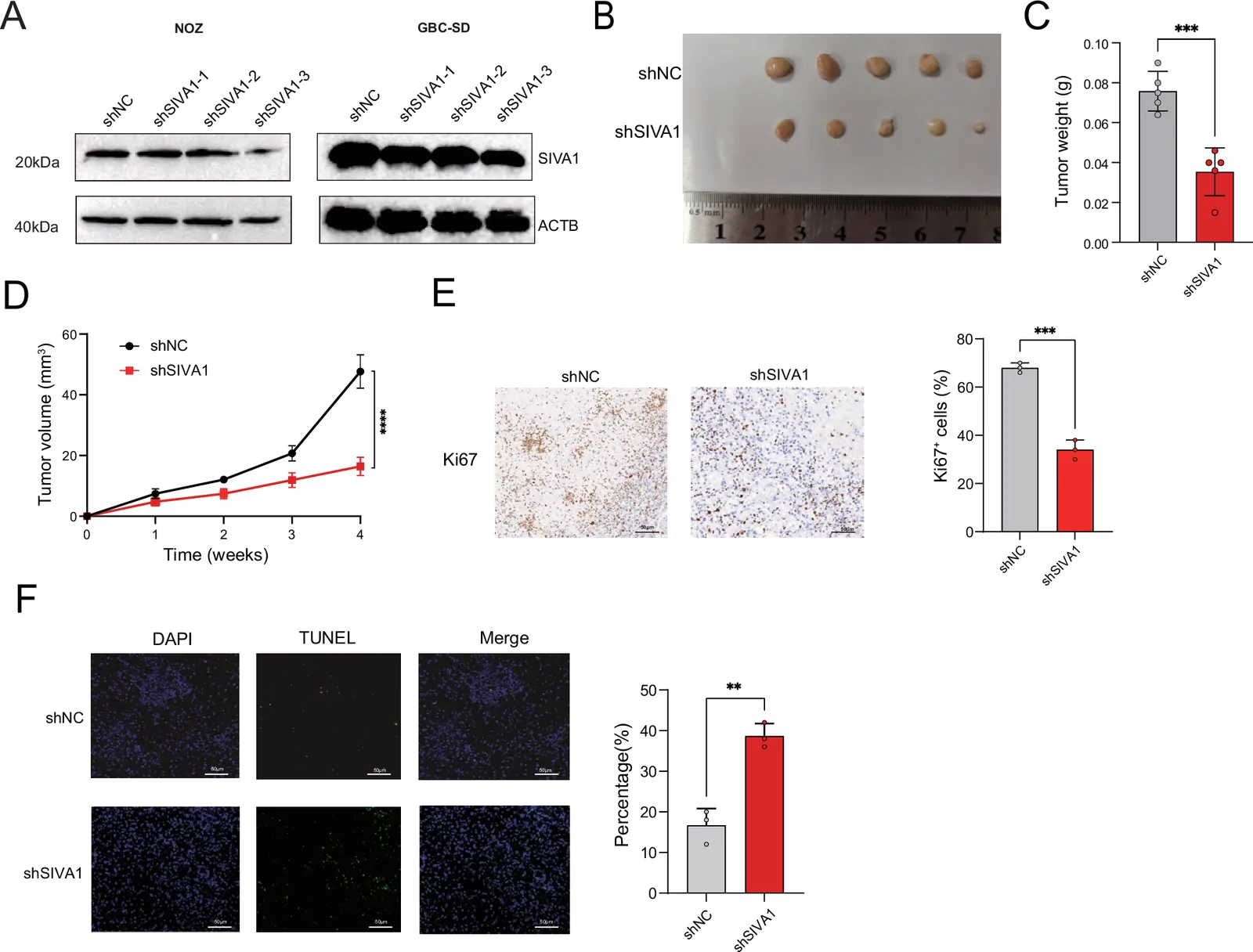
SIVA1 knockdown suppresses tumor growth in vivo. **A** Western blotting confirming SIVA1 knockdown in NOZ and GBC-SD cells transduced with shSIVA1 or shNC. Xenograft tumors formed by NOZ cells in nude mice (*n* = 5 mice/group); tumor morphology (**B**) and tumor weight (**C**). ***p < 0.001. **D** Tumor growth curves over 4 weeks. ***p < 0.001. **E** Ki67 IHC staining showing reduced proliferation in shSIVA1 tumors (scale bar = 50 µm). **F** TUNEL staining showing increased apoptosis in shSIVA1 tumors (scale bar = 50 µm). Data information: In vitro data are representative of three experiments with all data shown as mean ± SD.

To validate these in vitro findings in vivo, NOZ and GBC-SD cells stably expressing SIVA1-targeting shRNA (shSIVA1-3) or control shRNA (shNC) were subcutaneously injected into nude mice. Western blotting confirmed stable knockdown of SIVA1 (Fig. 3A). shSIVA1-3 was selected for in vivo studies based on its superior knockdown efficiency validated in both NOZ and GBC-SD cells (Fig. 3A). Tumor growth was monitored for four weeks. Mice injected with shSIVA1-expressing NOZ cells exhibited notably reduced tumor volume and weight compared to controls (Fig. 3B–D). IHC staining revealed markedly lower Ki67-positive cell density in SIVA1 knockdown tumors, while TUNEL staining showed a substantial increase in SIVA1 knockdown tumors (Fig. 3E, F). These results demonstrate that SIVA1 is required for efficient tumor growth in vivo and further support its oncogenic role in GBC.

### C2ORF68 interacts with SIVA1 and forms a stable protein complex

**Fig. 4:**
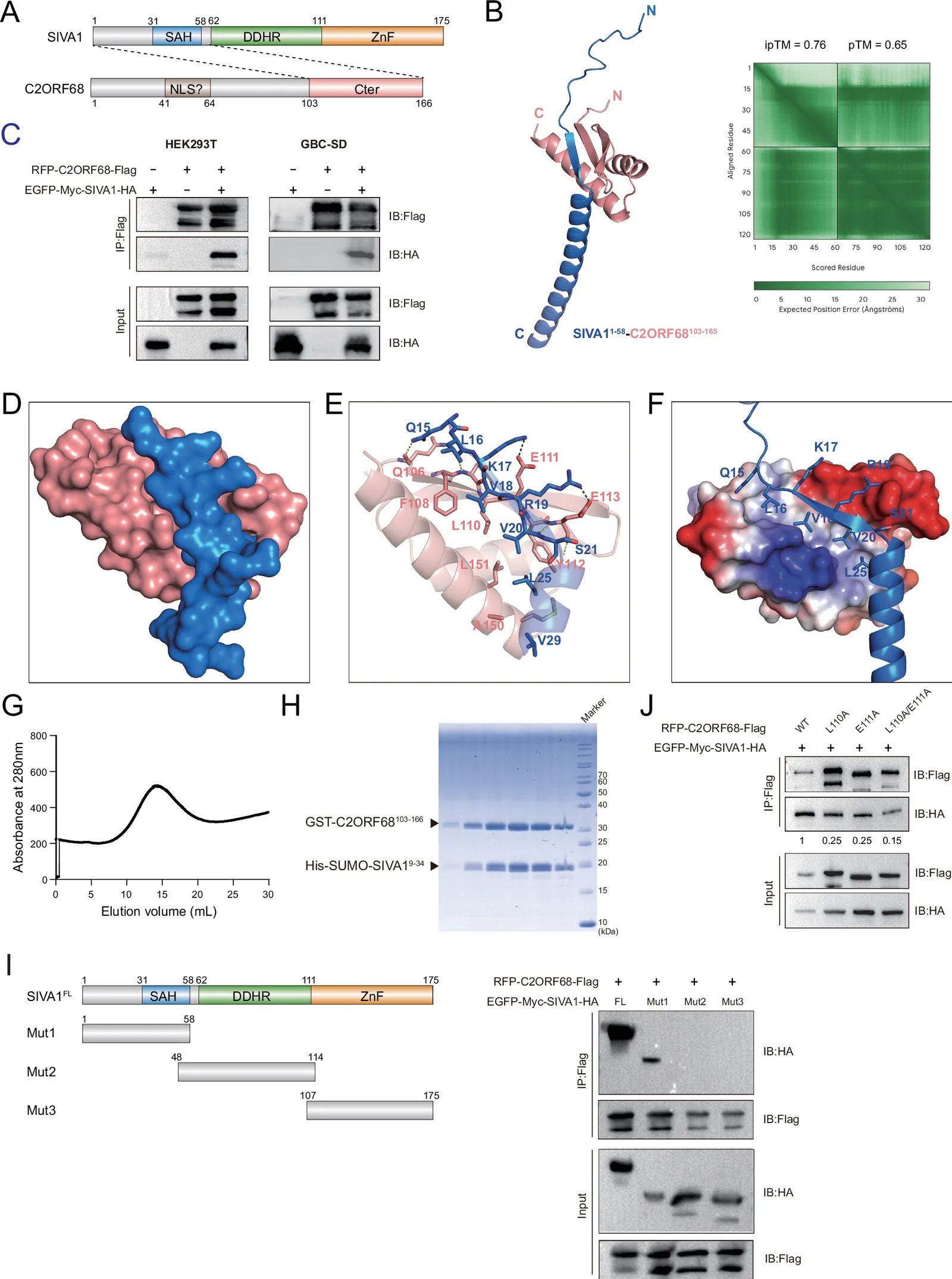
C2ORF68 interacts with SIVA1 to form a stable protein complex. **A** Domain organization of SIVA1 and C2ORF68. **B** AlphaFold 3 structural model and PAE plot (ipTM = 0.76). **C** Co-IP assays confirming intracellular interaction in HEK293T cells and GBC-SD cells. **D** Surface representation of the predicted complex interface. **E** Model showing SIVA1 Q15-V20 segment aligning along the C-terminal groove of C2ORF68. **F** Electrostatic surface potential of C2ORF68. **G** Elution profile of purified C2ORF68^103-166^-SIVA1^9-34^ complex. **H** SDS-PAGE confirming co-purification. **I** Mapping of SIVA1 truncation constructs tested for C2ORF68 binding. **J** Co-IP showing reduced interaction in C2ORF68 L110A, E111A, and L110/E111A mutants. Data information: The experiments were repeated three times.

To elucidate the molecular function of SIVA1, we sought to identify its interacting partners. SIVA1 consists of three structured regions: an N-terminal amphipathic helix (SAH; residues 31-55), a central death-domain–homology region (DDHR; residues 58-114), and a C-terminal zinc finger-like domain (ZnF; residues 112-170) (Fig. 4A, Fig. S1A). The functional relevance of these domains remains unclear. To explore possible interactors, large-scale human protein-protein interaction data of yeast two-hybrid were integrated with AlphaFold3 complex predictions. C2ORF68 emerged as the top candidate. C2ORF68 encodes a 166-amino acid protein located on chromosome 2p11.2 that contains an N-terminal nuclear localization signal and a conserved C-terminal UPF0561 domain (residues 103-166) (Fig. S1B).

AlphaFold3 modeling predicted a well-defined interface between SIVA1 residues 9–34 (SAH region) and the UPF0561 domain of C2ORF68 (residues 106-166), with a moderate-to-high confidence interface score (ipTM = 0.76) (Fig. 4B, Fig. S2A). Co-immunoprecipitation (Co-IP) in HEK293T cells and GBC-SD cells confirmed this interaction in cellulo (Fig. 4C). The predicted interface features a shallow hydrophobic groove in C2ORF68, formed by F108, L110, and Y112, accommodating the amphipathic helix of SIVA1. Residues Q15-V20 of SIVA1 insert into this groove and extend the β-sheet of C2ORF68 through backbone hydrogen bonding. Stabilizing interactions include hydrophobic contacts (F108, L110, Y112, A150, L151 with SIVA1 V18, V20, L25, V29), electrostatic pairing (E111/E113 with K17/R19), and a hydrogen bond between SIVA1 Q15 and C2ORF68 Q106 (Fig. 4D-F).

To confirm direct binding, His_6_-SUMO-SIVA1^9-34^ and GST-C2ORF68^103-166^ were co-expressed in E. coli. SIVA1^9-34^ alone was largely insoluble, whereas co-expression with C2ORF68 markedly increased solubility. Sequential GST and Ni-NTA purification yielded a symmetric peak containing a 1:1 heterodimer, verified by SDS-PAGE (Fig. 4G, H). These findings demonstrate that SIVA1 and C2ORF68 form a stable complex in vitro.

To map the binding region, three SIVA1 truncations were generated: Mut1 (residues 1–58), Mut2 (48–114), and Mut3 (107–175) (Fig. 4I). Co-IP assays showed that only the SAH-containing Mut1 fragment interacted with C2ORF68. To identify key interface residues within C2ORF68, L110A, E111A, and the L110/E111A double mutant were constructed. Co-IP assays revealed progressively reduced binding in these mutants (Fig. 4J), indicating that residues L110 and E111 are essential for stable SIVA1 association.

### C2ORF68 promotes gallbladder cancer cell proliferation in a SIVA1-dependent manner

**Fig. 5:**
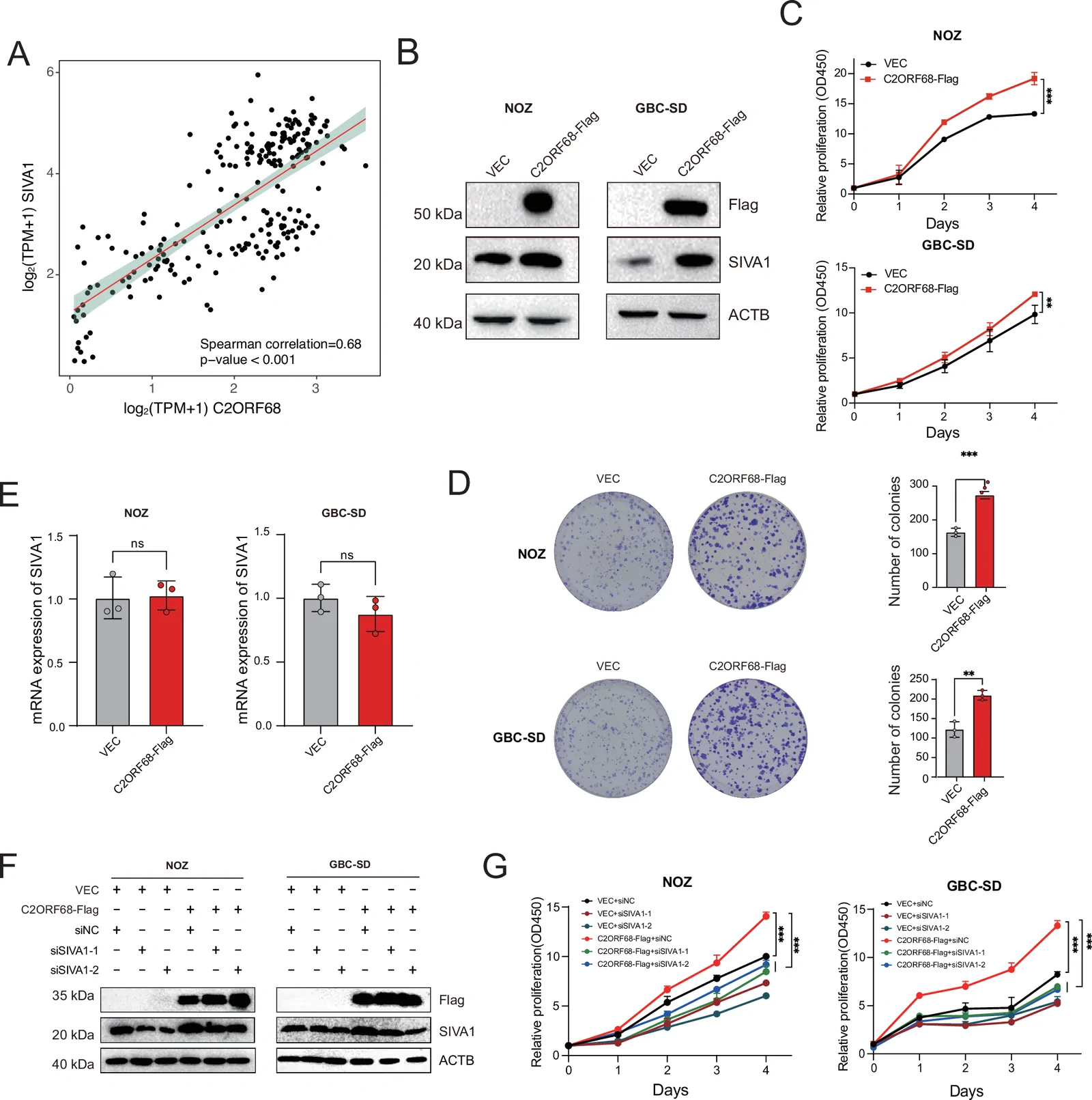
C2ORF68 promotes gallbladder cancer cell proliferation through SIVA1 stabilization. **A** A positive correlation between C2ORF68 and SIVA1 expression (Spearman *r* = 0.68, *P* < 0.001) was identified from an integrated analysis of multiple GBC datasets from GEO (SRP110184, SRP200495, SRP223526, SRP226150, SRP227857, SRP374181, and SRP459586), NODE (OEP00001237), DDBJ (DRP008090), and EGA (EGAS00001003004) databases. Statistical analysis was performed using the Spearman rank correlation test. **B** Western blotting showing increased SIVA1 protein after C2ORF68 overexpression in NOZ and GBC-SD cells. **C** CCK-8 assays showing enhanced proliferation after C2ORF68 overexpression. **D** Colony formation assays showing increased colony number and size. **E** qRT-PCR showing no change in SIVA1 mRNA. **F** Western blotting confirming SIVA1 knockdown in cells co-transfected with C2ORF68 and siSIVA1. **G** CCK-8 assays showing that SIVA1 knockdown abolishes the pro-proliferative effect of C2ORF68. Data information: The experiments were repeated three times and data shown as the mean ± SD.

To substantiate the physiological relevance of the SIVA1-C2ORF68 interaction identified above, we first examined their endogenous expression patterns through an integrated analysis of multiple GBC datasets from GEO, NODE, DDBJ, and EGA databases. This analysis revealed a positive correlation between C2ORF68 and SIVA1 mRNA expression (Spearman *rho* = 0.68, *P* < 0.001; Fig. 5A). This correlated expression pattern was further supported by TCGA pan-cancer datasets, where weak-to-modest but significant positive correlations were detected in multiple cancer types, including thyroid carcinoma (THCA, Spearman *rho* = 0.347, *P* < 0.001), acute myeloid leukemia (LAML, Spearman *rho* = 0.343, *P* < 0.001), and stomach adenocarcinoma (STAD, Spearman *rho* = 0.29, *P* < 0.001) (Fig. S4). Together with the protein-protein interaction and in vitro reconstitution data described above, these clinical observations support the physiological relevance of the C2ORF68-SIVA1 complex and suggest that this axis may operate in GBC and other epithelial malignancies.

Notably, C2ORF68 has been previously reported to be upregulated in colorectal cancer, where it promotes tumor proliferation and migration through PI3K/Akt/mTOR and Wnt/β-catenin signaling [24]. Based on this clinical evidence and the established oncogenic role of C2ORF68 in other malignancies, we then investigated whether C2ORF68 exerts similar oncogenic effects in GBC. NOZ and GBC-SD cells were transiently transfected with a C2ORF68 overexpression plasmid. Because no commercial antibody is currently available for C2ORF68, a C-terminal Flag tag was introduced to facilitate detection (Fig. 5B). CCK-8 assays showed that C2ORF68 overexpression led to enhanced proliferation in both cell lines (Fig. 5C), and colony formation assays revealed a corresponding increase in colony number and size (Fig. 5D).

To assess whether this oncogenic effect is mediated through SIVA1, we examined SIVA1 expression following C2ORF68 overexpression. qRT-PCR showed no appreciable change in SIVA1 mRNA (Fig. 5E), whereas Western blotting demonstrated a substantial increase in SIVA1 protein abundance (Fig. 5B), indicating post-transcriptional regulation. Given that SIVA1 promotes GBC cell proliferation, we hypothesized that C2ORF68 requires SIVA1 to exert its oncogenic effect. Rescue experiments were conducted by co-transfecting C2ORF68 plasmids with SIVA1-targeting siRNAs. Western blotting confirmed efficient C2ORF68 overexpression and SIVA1 knockdown (Fig. 5F). As expected, SIVA1 depletion abolished the growth-promoting effect of C2ORF68 (Fig. 5G). These results suggest that C2ORF68 enhances GBC cell proliferation, at least in part, through stabilizing SIVA1 protein.

### C2ORF68 stabilizes SIVA1 by modulating its ubiquitin-mediated proteasomal degradation

**Fig. 6:**
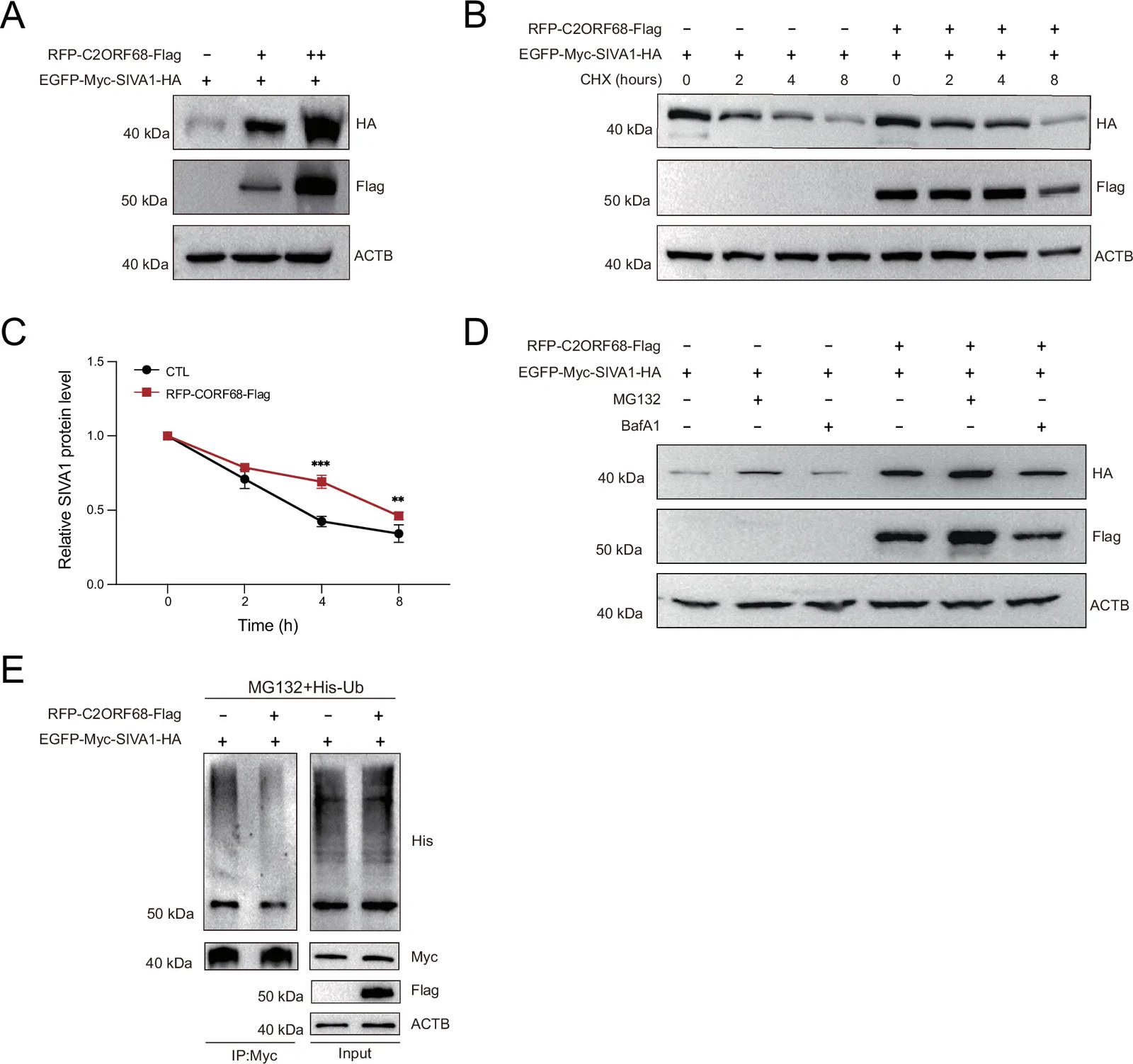
C2ORF68 stabilizes SIVA1 by inhibiting its ubiquitination. **A** Dose-dependent increase in SIVA1 protein with increasing C2ORF68 expression. **B** CHX chase assays showing extended SIVA1 half-life in presence of C2ORF68. **C** Quantification of SIVA1 degradation rates. **D** MG132, but not bafilomycin A, increases SIVA1 levels; C2ORF68 further enhances accumulation. **E** Ubiquitination assay showing reduced SIVA1 polyubiquitination upon C2ORF68 expression. Data information: The experiments were repeated three times and data shown as the mean ± SD.

To elucidate how C2ORF68 stabilizes SIVA1, HEK293T cells were co-transfected with EGFP-Myc-SIVA1-HA and increasing amounts of RFP-C2ORF68-Flag. Western blotting showed a dose-dependent increase in SIVA1 protein, indicating that C2ORF68 enhances SIVA1 stability (Fig. 6A). Cycloheximide (CHX) chase assays were then performed to assess SIVA1 protein turnover [25]. In control cells, SIVA1 levels began to decline markedly after 2 h of CHX treatment, whereas C2ORF68 overexpression delayed this reduction until ~4 h. SIVA1 levels also remained higher in C2ORF68-overexpressing cells at later time points, indicating that C2ORF68 slows SIVA1 degradation (Fig. 6B, C).

To identify the degradation pathway involved, HEK293T cells expressing SIVA1 with or without C2ORF68 were treated with MG132 (proteasome inhibitor) [26] or BafA1 (autophagy inhibitor) [27]. MG132 markedly increased SIVA1 levels, whereas BafA1 had minimal effect. Importantly, C2ORF68 overexpression further increased SIVA1 abundance in MG132-treated cells (Fig. 6D), which is consistent with the role of C2ORF68 in modulating the ubiquitin-proteasome pathway.

To directly assess ubiquitination, cells were co-transfected with EGFP-Myc-SIVA1-HA and His-ubiquitin, with or without C2ORF68 overexpression, and treated with MG132. Immunoprecipitation followed by Western blotting revealed extensive ubiquitination of SIVA1 in control cells, whereas C2ORF68 overexpression substantially reduced ubiquitinated SIVA1 species (Fig. 6E). These results suggest that C2ORF68 stabilizes SIVA1 potentially by attenuating its ubiquitin-mediated proteasomal degradation.

### SIVA1 suppresses apoptosis in gallbladder cancer cells

**Fig. 7:**
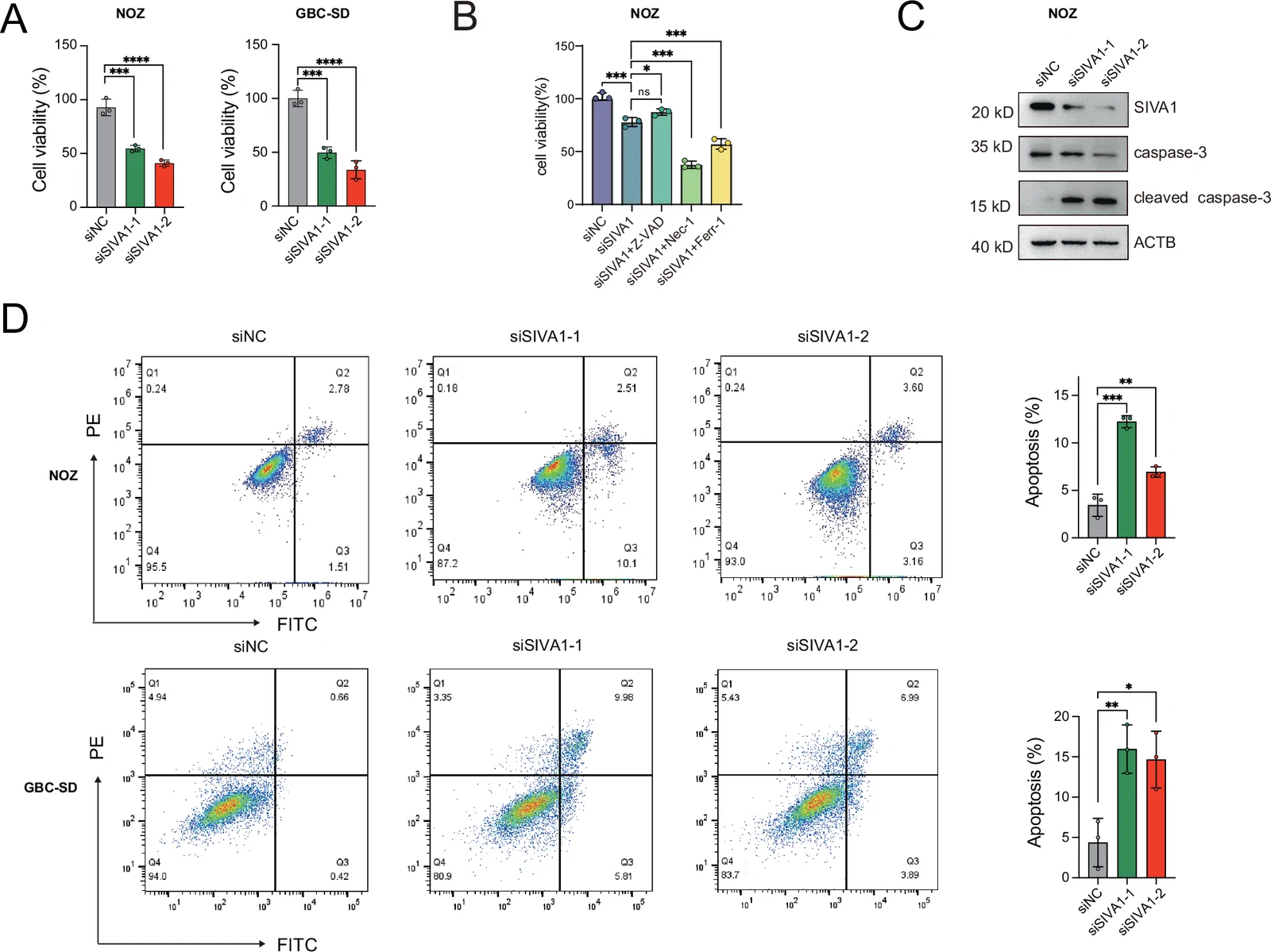
SIVA1 suppresses apoptosis in gallbladder cancer cells. **A** CCK-8 assays of NOZ and GBC-SD cells following transfection with siNC or siSIVA1. **B** Cell viability assessed in SIVA1-depleted cells treated with Z-VAD (30 μM), Nec-1 (20 μM), or Ferr-1 (20 μM). All data were normalized to the siNC + DMSO control group. Statistical significance was determined by two complementary one-way ANOVA analyses with Dunnett’s post-hoc test. Data information: The experiments were repeated three times and data shown as the mean ± SD; *P < 0.05. **C** Western blotting showing increased cleaved caspase-3 after SIVA1 knockdown. **D** Flow cytometry confirming increased apoptosis in NOZ and GBC-SD cells. Data information: The experiments were repeated three times and data shown as the mean ± SD.

We next investigated how SIVA1 influences GBC cell proliferation. SIVA1 has been reported to function as a tumor-promoting factor in non-small cell lung cancer (NSCLC) [22], suggesting that it may similarly support cell survival in GBC. Consistent with this idea, CCK-8 assays showed that SIVA1 knockdown markedly reduced cell viability in both NOZ and GBC-SD cells (Fig. 7A), indicating that SIVA1 is required for maintaining GBC cells survival.

Given that SIVA1 interacts with multiple apoptosis-related proteins [14, 28, 29], we examined whether the reduced viability observed upon SIVA1 depletion was due to apoptosis or other cell death types. Three selective inhibitors were tested: Z-VAD-FMK (Z-VAD), a pan-caspase inhibitor that blocks apoptosis [30]; Necrostatin-1 (Nec-1), a RIP1 kinase inhibitor that suppresses necroptosis [31]; and Ferrostatin-1 (Ferr-1), a ferroptosis inhibitor that prevents lipid peroxidation [32]. Following 48 h treatment, only Z-VAD partially restored viability in SIVA1-silenced cells, whereas Nec-1 and Ferr-1 had minimal effect (Fig. 7B), suggesting that apoptosis is the primary mode of cell death induced by SIVA1 depletion.

Western blotting analysis further confirmed a marked increase in cleaved caspase-3 levels after SIVA1 knockdown (Fig. 7C). In addition, Annexin V-FITC/propidium iodide (PI) flow cytometry demonstrated elevated apoptotic cell populations in both NOZ and GBC-SD cells lacking SIVA1 (Fig. 7D). Together, these results demonstrate that SIVA1 promotes GBC cells survival primarily by suppressing apoptosis.

### SIVA1 promotes GBC cell proliferation by upregulating BCL-2

**Fig. 8:**
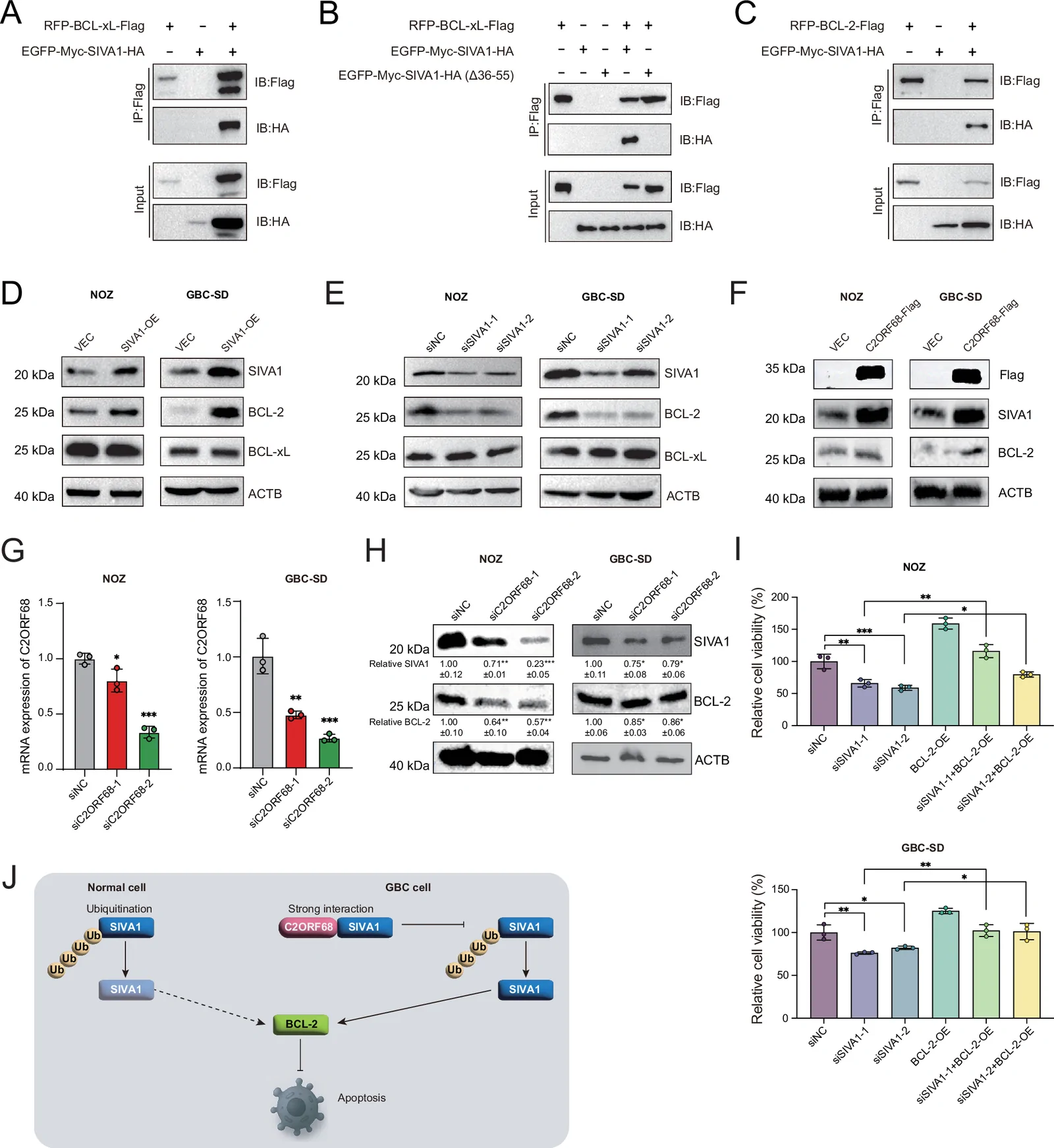
SIVA1 promotes cell survival by selectively stabilizing BCL-2. **A** Co-IP confirming interaction between SIVA1 and BCL-xL. **B** Deletion of SIVA1 SAH (Δ36–55) abolishes interaction with BCL-xL. **C** Co-IP confirming interaction between SIVA1 and BCL-2. **D,E** Western blotting showing selective regulation of BCL-2 but not BCL-xL. **F** C2ORF68-Flag overexpression increases both SIVA1 and BCL-2 protein levels. **G** RT-qPCR validation of C2ORF68 knockdown efficiency using two independent siRNAs (siC2ORF68-1 and siC2ORF68-2) in NOZ and GBC-SD cells. **H** C2ORF68 knockdown reduces both SIVA1 and BCL-2 protein levels. Quantified band intensities from three independent biological replicates are shown below the blots as mean ± SD. SIVA1 and BCL-2 signals were normalized to ACTB and then to the corresponding siNC control within each cell line. Statistical significance was calculated separately for each cell line relative to siNC. *P < 0.05, **P < 0.01, ***P < 0.001. **I** CCK-8 rescue assays showing BCL-2 overexpression restores proliferation suppressed by SIVA1 knockdown. The proliferation ratio (*OD*_*48h*_/*OD*_*Day1*_) was calculated for each group and normalized to the siNC control to determine relative growth capacity (%); values > 100% reflect enhanced growth capacity relative to the baseline. **J** Schematic model illustrating how C2ORF68-mediated stabilization of SIVA1 increases BCL-2 and suppresses apoptosis. Data information: The experiments were repeated three times and data shown as the mean ± SD.

To investigate the anti-apoptotic mechanism of SIVA1, we examined its interactions with BCL-2 family proteins [33]. Co-IP assays in HEK293T cells confirmed that SIVA1 associates with BCL-xL (Fig. 8A), and deletion of the SIVA1 SAH motif (residues 36–55) abolished this interaction (Fig. 8B), indicating that the SAH region mediates binding. Given the high sequence similarity (~70%) between BCL-2 and BCL-xL (also known as BCL2L1; Fig. S3A), we tested whether SIVA1 also interacts with BCL-2. Co-IP assays confirmed this binding (Fig. 8C).

We next evaluated whether SIVA1 influences the abundance of BCL-2 and BCL-xL. In both NOZ and GBC-SD cells, SIVA1 overexpression increased BCL-2 protein levels, whereas SIVA1 knockdown reduced BCL-2; with no corresponding changes in BCL-xL (Fig. 8D, E). qRT-PCR analysis showed no alterations in BCL-2 or BCL-xL mRNA, indicating post-transcriptional regulation (Fig. S3B). Furthermore, we examined whether the upstream stabilizer C2ORF68 similarly regulates BCL-2 levels. Western blotting revealed that C2ORF68 overexpression increased BCL-2 protein levels (Fig. 8F), whereas its depletion by two independent siRNAs, with knockdown efficiency validated at the mRNA level owing to the absence of a commercially available C2ORF68 antibody (Fig. 8G), markedly reduced BCL-2 abundance (Fig. 8H), supporting that C2ORF68 maintains BCL-2 levels through SIVA1 stabilization. The effect was less pronounced in GBC-SD cells than in NOZ cells, suggesting that the C2ORF68-SIVA1-BCL-2 axis may be more robust in NOZ cells. Rescue experiments further demonstrated that overexpression of BCL-2 restored relative cell viability reduced by SIVA1 knockdown (Fig. 8I).

Together, these findings suggest that SIVA1 interacts with both BCL-xL and BCL-2, and its selective stabilization of BCL-2 may contribute to the suppression of apoptosis and promotion of GBC cell proliferation (Fig. 8J).

## Discussion

This study identifies SIVA1 as a tumor-promoting factor in gallbladder cancer and characterizes C2ORF68 as a previously unstudied SIVA1-binding protein that stabilizes SIVA1 and enhances its pro-survival function. We show that C2ORF68 prevents the ubiquitin–proteasome–mediated degradation of SIVA1, resulting in increased BCL-2 protein abundance and suppression of apoptosis. These findings reveal a previously unrecognized post-translational regulatory mechanism contributing to gallbladder cancer progression.

SIVA1 was originally described as a pro-apoptotic protein and a binding partner of CD27. However, accumulating evidence suggests that its biological role is highly context dependent [20, 34, 35]. In breast cancer cells, SIVA1 interacts with the anti-apoptotic protein BCL-xL through its N-terminal SAH and enhances sensitivity to apoptotic stimuli [17]. In contrast, our results demonstrate that in GBC, SIVA1 primarily supports cell survival. SIVA1 depletion triggers apoptosis and reduces cell viability, whereas its overexpression enhances proliferation. These observations highlight that the functional outcome of SIVA1 expression depends on cell type and the set of interacting partners present.

The ZnF domain of SIVA1 is the least understood among its structured regions. Given that zinc finger motifs commonly participate in nucleic acid or protein binding [36, 37], this region may mediate additional molecular interactions or influence subcellular localization. Structural prediction suggests that the ZnF domain forms a compact zinc-binding cluster, potentially stabilizing protein conformation or interaction surfaces. Further work will be needed to determine whether this domain contributes independently to the pro-survival function of SIVA1 or cooperates with the SAH region.

C2ORF68 has not been functionally characterized in most tumor settings [24, 38]. Our study provides the first mechanistic insight into its role in GBC. Sequence analysis indicates that C2ORF68 contains a conserved UPF0561 domain, whose biological function has not been defined. Structural modeling predicted a moderate-to-high confidence interaction interface between this domain and the SIVA1 SAH region, mediated by hydrophobic and electrostatic contacts; mutational analysis confirmed the essential roles of L110 and E111 in maintaining this interaction. Functionally, C2ORF68 increases SIVA1 protein levels without altering SIVA1 mRNA abundance and markedly reduces SIVA1 polyubiquitination, indicating that it acts as a stabilizing regulator. However, the concomitant stabilization of C2ORF68 by MG132 (Fig. 6D) suggests that the increased abundance of C2ORF68 under proteasomal inhibition likely contributes to the robust stabilization of SIVA1. Notably, while SIVA1 is a known transcriptional target of p53, the steady-state levels of p53 protein in wild-type NOZ and GBC-SD cells are typically low due to rapid turnover in the absence of exogenous stress. Our findings suggest that C2ORF68-mediated protein stabilization represents a post-translational regulatory layer that likely complements p53-dependent transcription. The presence of a predicted nuclear localization signal suggests that C2ORF68 may also modulate the spatial distribution of SIVA1, a possibility that will require additional investigation.

The coordinated regulation observed among C2ORF68, SIVA1, and BCL-2 suggests a mechanism linking protein stability control to apoptosis resistance. Although we did not isolate a ternary complex, our data support a model in which C2ORF68 maintains SIVA1 stability, and stabilized SIVA1 selectively maintains BCL-2 protein levels without affecting BCL-xL. While these results point to a potential functional axis, we acknowledge that the signaling landscape in GBC is complex, and the involvement of other intermediate regulatory factors cannot be fully excluded. This selectivity suggests post-translational control of BCL-2, potentially by shielding it from E3 ubiquitin ligases or facilitating deubiquitination [39]. Similarly, while our data demonstrate that C2ORF68 reduces SIVA1 polyubiquitination, the specific E3 ligases or deubiquitinases that directly govern this process remain to be identified. C2ORF68 may function by sterically shielding SIVA1 from its cognate E3 ligases or by facilitating the recruitment of deubiquitinating enzymes. Determining whether SIVA1 directly recruits deubiquitinases or indirectly influences BCL-2 turnover will require biochemical reconstitution and proteomic mapping.

These findings have therapeutic implications. The defined C2ORF68–SIVA1 interaction interface presents a potential site for small-molecule disruption. Inhibiting this interaction may destabilize SIVA1 and sensitize gallbladder cancer cells to apoptosis. Structure-guided design strategies, including AlphaFold3-assisted modeling, could be used to identify compounds that occupy the hydrophobic binding interface. Moreover, combining such agents with clinically available BCL-2 inhibitors such as ABT-737 [40] or Navitoclax [41], may produce synergistic anti-tumor effects by simultaneously reducing BCL-2 accumulation and blocking its function. Because SIVA1 is upregulated in multiple digestive system cancers, similar regulatory mechanisms may be operative beyond GBC.

## Materials and methods

### Clinical specimens

All patients enrolled in this study were diagnosed with primary gallbladder cancer, confirmed by histopathological examination. Complete clinical and follow-up data were available for all participants. None of the patients received chemotherapy, radiotherapy, or other anticancer treatments before surgery. Tumor specimens were collected with informed consent from the patients and their families. All procedures were approved by the institutional ethics committee (Approval No. XHEC-NSFC-2023-214).

### Cell lines and culture

H69 cells were obtained from the Department of Biliary-Pancreatic Surgery, Renji Hospital, Shanghai Jiao Tong University School of Medicine. HEK293T, NOZ, ZJU-0430, SGC-996, and GBC-SD cell lines were acquired from the Shanghai Institute for Biological Sciences, Chinese Academy of Sciences (Shanghai, China). Based on previous genomic characterization [42], both NOZ and GBC-SD cell lines used in this study harbor wild-type TP53. Cells were cultured in Dulbecco’s Modified Eagle Medium (DMEM, Gibco) supplemented with 10% fetal bovine serum (FBS, Gibco) and 1% penicillin-streptomycin (Sangon, China) at 37 °C in a humidified incubator with 5% CO_2_.

### Quantitative real-time PCR (qRT-PCR)

Total RNA was extracted using TRIzol reagent (Takara, Japan) and reverse-transcribed into cDNA using the Advance Fast 1st Strand cDNA Synthesis Kit (Yeasen, China). Quantitative real-time PCR was performed using SYBR Green Master Mix (Yeasen, China) on a real-time PCR detection system (RocGene Technology, China). Gene expression levels were normalized to GAPDH and calculated using the ΔΔCt method. Primer sequences are listed in Supplementary Table 1.

### Western blotting

Cells were lysed on ice in RIPA buffer containing 1 mM PMSF for 30 min. Protein concentrations were measured using a BCA assay (Beyotime, China). Equal amounts of protein (20 µg) were denatured in 2× SDS loading buffer at 95 °C for 5 min, separated on 10–15% SDS-PAGE gels, and transferred to PVDF membranes. Membranes were blocked in 5% non-fat milk in TBST for 30 min at room temperature, incubated with primary antibodies (1:1000) for 4 h, and then with HRP-conjugated secondary antibodies (1:5000) for 1 h. Signals were detected using enhanced chemiluminescence (Beyotime, China) on a Tanon imaging system, and band intensities quantified using ImageJ. Antibodies are listed in Supplementary Table 2.

### RNA interference and plasmid transfection

Small interfering RNAs (siRNAs) targeting *SIVA1* were transfected using Lipofectamine 3000 (Thermo Fisher Scientific) according to the manufacturer’s instructions. The siRNA sequences were as follows:

si-*SIVA1*-1: sense 5′-GCAGUGACAUGUACGAGAATT-3′; antisense 5′-UUCUCGUACAUGUCACUGCTT-3′

si-*SIVA1*-2: sense 5′-CAGUGACAUGUACGAGAAATT-3′; antisense 5′-UUUCUCGUACAUGUCACUGTT-3′

(siRNAs synthesized by Sangon, China).

Short hairpin RNAs (shRNAs) candidates screened in this study are provided:

shSIVA1-1 (Target: 5′-GCAGTGACATGTACGAGAA-3′): 5′-CCGGGCAGTGACATGTACGAGAACTCGAGTTCTCGTACATGTCACTGCTTTTTT-3′

shSIVA1-2 (Target: 5′-AGGTCTTCGAGAAGACCAA-3′): 5′- CCGGAGGTCTTCGAGAAGACCAACTCGAGTTGGTCTTCTCGAAGACCTTTTTTT-3’

shSIVA1-3 (Target: 5′-CGCAGGAGGTCTTCGAGAA-3′): 5′-CCGGCGCAGGAGGTCTTCGAGAACTCGAGTTCTCGAAGACCTCCTGCGTTTTTT-3’

(shRNAs synthesized by Tsingke, China).

si-*C2ORF68*-1: sense 5′-CAUCAGUUAUCGUCUAUCA-3′; antisense 5′- UGAUAGACGAUAACUGAUG-3′

si-*C2ORF68*-2: sense 5′-GUGAGAAGGUGUCGGCACA-3′; antisense 5′- UGUGCCGACACCUUCUCAC-3′

(siRNAs synthesized by Sangon, China).

Full-length SIVA1, C2ORF68, BCL2, and BCL2L1 coding sequences were cloned into pCMV vectors, with empty vectors as controls. SIVA1 truncations and C2ORF68 or BCL-xL mutants were generated using PCR-based cloning. Plasmid transfections were performed using Lipofectamine 3000. Primer sequences for construct generation are provided in Supplementary Table 3.

### Cell viability (CCK-8) assay

Cell viability was measured using the CCK-8 assay. A total of 2 × 10³ cells were seeded per well in 96-well plates. After incubation with 10 µL of CCK-8 solution (Yeasen, China) at 37 °C for 2 h, absorbance at 450 nm was recorded using a Multiskan Sky microplate reader (Thermo Fisher Scientific) every 24 h for up to 96 h. Cell viability (%) was calculated using the formula [(*OD*_*Treatment*_-*OD*_*Blank*_)/(*OD*_*control*_-*OD*_*Blank*_)]×100%.

For relative cell proliferation assays, the proliferation ratio (*OD*_*48h*_/*OD*_*Day1*_) was calculated for each group and normalized to the control group to yield relative proliferation (%).

### Colony formation assay

Cells (1×10³ per well) were seeded in 6-well plates and cultured in 2 mL medium containing 10% FBS for two weeks. Colonies were fixed with 4% paraformaldehyde (PFA) and stained with 0.1% crystal violet. Colony numbers were counted to assess clonogenic potential.

### Transwell migration assay

Migration assays were conducted using 24-well plates equipped with 8 µm transwell chambers (NEST, China). A total of 5 × 10^4^ cells in serum-free medium were seeded into the upper chamber, while the lower chamber contained medium supplemented with 10% FBS medium as a chemoattractant. After 24 h, non-migrated cells were removed with cotton swabs. Migrated cells on the lower membrane surface were fixed with 4% PFA for 30 min, stained with 0.1% crystal violet for 15 min, and counted in five random microscopic fields.

### Flag beads pull-down assay

HEK293T cells were transfected with recombinant plasmids using PEI (Beyotime, China) and harvested 24 h post-transfection. Lysates were incubated overnight at 4 °C with anti-Flag agarose beads (Sigma-Aldrich), washed three times with buffer (20 mM Tris, pH 8.0, 150 mM NaCl, 0.1% Triton X-100), and eluted by boiling in 2× SDS loading buffer. Bound proteins were analyzed by Western blotting.

### Protein expression and purification

The human SIVA1 (residues 9–34) and C2ORF68 (residues 103-166) fragments were co-expressed in *E. coli* BL21 (DE3) cells. The SIVA1 fragment was cloned into pET28a containing an N-terminal His_6_-SUMO tag, and the C2ORF68 fragment into pGEX-6p-1 with an N-terminal GST tag. Co-transformed cells were grown in LB medium supplemented with 50 µg/mL kanamycin and 100 µg/mL ampicillin at 37 °C until the optical density (OD₆₀₀) reached 0.6. Protein expression was induced with 0.2 mM IPTG, and cultures were incubated overnight at 16 °C.

Cells were harvested by centrifugation at 4000 rpm for 15 min at 4 °C and lysed using a French press (JNBIO) in lysis buffer containing 20 mM Tris, pH 8.0, 150 mM NaCl, 1 mM DTT, and protease inhibitor cocktail. The lysate was clarified by centrifugation at 12,000 × *g* for 30 min at 4 °C, and the supernatant was sequentially passed through a GSTrap HP column (Cytiva) and a HisTrap affinity column (Cytiva). Bound proteins were eluted with buffers containing 10 mM reduced glutathione or 250 mM imidazole, respectively. Protein purity were verified by SDS-PAGE and Coomassie blue staining.

### Xenograft mouse models

Six-week-old female BALB/c nude mice (18-20 g) were randomly divided into two groups (Conrtrol group and SIVA1 knockdown group) of five and maintained under specific pathogen-free (SPF) conditions. After one week of acclimation, each mouse was subcutaneously injected with 1 × 10^6^ NOZ cells in 50 µL PBS mixed 1:1 with 50 µL Matrigel. Tumor size was measured weekly using calipers, and volume was calculated as *V* = *π/6 × width* [2] *× length*. After four weeks, mice were euthanized, and tumors were excised, weighed, and processed for analysis. All animal experiments were approved by the Animal Research Ethics Committee of Xinhua Hospital, Shanghai Jiao Tong University School of Medicine (Approval No. XHEC-NSFC-2023-214).

### Co-immunoprecipitation

Cells were lysed in ice-cold IP buffer (20 mM Tris, pH 7.4, 150 mM NaCl, 1% NP-40, 1 mM EDTA, 5% glycerol, and protease inhibitor cocktail). Lysates were centrifuged at 12,000 × *g* for 10 min at 4 °C, and the supernatant was incubated with 2–5 µg of the indicated antibody overnight at 4 °C with rotation. Protein A/G agarose beads (MCE, China) were added and incubated for an additional 2 h at 4 °C. After three washes with IP buffer, bound proteins were eluted by boiling in SDS loading buffer and analyzed by Western blotting.

### Structural modeling

The structural interaction between SIVA1 and C2ORF68 was predicted using AlphaFold Multimer (AlphaFold 3, Google DeepMind). Full-length or truncated version of sequences corresponding to human SIVA1 and C2ORF68 were used as inputs. The predicted interface confidence was evaluated by the interface predicted template modeling (ipTM) score. Molecular visualization and interface analysis were performed in PyMOL v3.1 (Schrödinger LLC).

### Cycloheximide chase assay

HEK293T cells were co-transfected with Flag-tagged C2ORF68 and Myc-tagged SIVA1 plasmids for 24 h, followed by CHX treatment (final concentration of 50 µg/mL) for 0, 2, 4, or 8 h. Cells were lysed and analyzed by Western blotting to evaluate SIVA1 stability. Band intensities were quantified using ImageJ to determine SIVA1 degradation rates.

### Ubiquitination assay

HEK293T cells were co-transfected with Flag-tagged C2ORF68, Myc-tagged SIVA1, and His-tagged ubiquitin plasmids for 48 h. Cells were treated with MG132 (10 µM) for 6 h before harvest. Lysates were prepared in buffer containing 20 mM Tris, pH 7.4, 150 mM NaCl, 0.1 mM EDTA, 1% NP-40, 5% glycerol, 1% protease inhibitor cocktail, and 1 mM PMSF. Lysates were centrifuged at 12,000 × *g* for 5 min at 4 °C, supernatants were incubated overnight at 4 °C with 5 µg anti-Myc antibody, followed by Protein A/G agarose beads (MCE, China) for 2 h. After three washes, bound proteins were eluted by boiling in SDS loading buffer and analyzed by Western blotting.

### Apoptosis assay

Apoptosis was evaluated using the Annexin V-FITC/PI dual-staining kit (Beyotime, China) following the manufacturer’s protocol. Cells (5 × 10^5^) were collected, washed with PBS, and resuspended in 100 µL binding buffer containing 5 µL Annexin V-FITC and 5 µL PI. After incubation for 20 min at room temperature in the dark, samples were analyzed by flow cytometry (Beckman).

### Statistical analysis

All statistical analyses were performed using GraphPad Prism 9.0. Data are expressed as mean ± SD. Normal distribution was assessed prior to testing. Differences between two groups were analyzed using an unpaired two-tailed *t* test, and multiple-group comparisons were analyzed by one-way ANOVA with Dunnett’s post hoc test. Significance levels are indicated as follows: ns indicates no significant difference, *p < 0.05, **p < 0.01, ***p < 0.001.

## Supplementary information

Supplementary Material

Uncropped western blots

## Data Availability

Uncropped Western blotting images are available in the Supplementary Material.
