# Supplementary material for: C2ORF68 stabilizes SIVA1 to upregulate BCL-2 and suppress apoptosis in gallbladder cancer
Source: Cell Death Discov. 2026 Jul 30;12:413. doi: 10.1038/s41420-026-03279-5 (PMC13652811; doi:10.1038/s41420-026-03279-5)
Supplement: Supplementary file 1 [file 41420_2026_3279_MOESM1_ESM.pdf]

SUPPLEMENTARY MATERIALS

C2ORF68 stabilizes SIVA1 to upregulate BCL-2 and suppress apoptosis in gallbladder cancer

Xuanjia Dong, Lingxiao Zhang, Wenjun Dong, Qiuyi Tang, Jingwei Zhao, Zhaohui Jin, Lin Jiang, Wenting Dai, Yangyang Zhai, Ying Huang

Supplementary Table 1. qRT-PCR primers used in this study.

Primer	Sequence (5'-3')
BCL-xL-F	CCCAGAAGGGACTGAATCGG
BCL-xL-R	GCAGTGGCTCCATTCACCG
BCL-2-F	AAAAATACAACATCACAGAGGAAGT
BCL-2-R	AGGGGGTGTCTTCAATCACG
SIVA1-F	CTCCGAAGCTGACCCATCTG
SIVA1-R	ATGTCACTGCAGTCCACGAG
GAPDH-F	TGCACCACCAACTGCTTAG
GAPDH-R	GGATGCAGGGATGATGTTC

Supplementary Table 2. Antibodies used in this study.

Antibody	Sources	Catalog
Anti-BCL-2	Affinity Biosciences	AF6139
Anti-ACTB	ABclonal	AC038
Anti-BCL-xL	ABclonal	A23766
Anti-HA	ABclonal	AE105
Anti-Flag	Beyotime	AF2855
Anti-cleaved caspase-3	Affinity Biosciences	AF7022
Anti-caspase-3	ABclonal	A19654
Anti-Myc	ABclonal	AE070

Supplementary Table 3. PCR primers.

Primer	Sequence (5'-3')
RFP-BCL-xL-F	CACTCCACCGGCGCCCCTATGTCTCAGAGC
BCL-xL-Flag-R	CGTCATCGTCTTTGTAGTCCTCTTTCCGA
RFP-BCL-2-F	ACTCCACCGGCGCCCCTATGGCGCACGCTGGGAGA
BCL-2-Flag-R	ATCGTCTTTGTAGTCCTCCTTGTGGCCCAGATA
C2ORF68-L110A-F	TTCTGCGCAGAATACGAGGCAGACAGTGGAGA
C2ORF68-L110A-R	CTCGTATTCTGCGCAGAAGAGCTGATGGC
C2ORF68-E111A-F	CACTGTCTGCCTCGTATGCTAAGCAGAAGAGCT
C2ORF68-E111A-R	GCTTAGCATACGAGGCAGACAGTGGAGAGGTC
C2ORF68-L110/E111A-F	TGCCTCGTAGGCTGCGCAGAAGAGCTGATGG
C2ORF68-L110/E111A-R	CTGCGCAGCCTACGAGGCAGACAGTGGA
RFP-C2ORF68-FL-F	ACTCCACCGGCGCCCCTATGGAGGCGGGGCC
C2ORF68-FL-Flag-R	CGTCATCGTCTTTGTAGTCCTCGTGTTGGCTC
EGFP-Myc-SIVA1-1-F	TGAAGAGGACTTGTCCGGTTCCATGCCCAAGCG
SIVA1-58-HA-R	GGGTAGGAACCGGAACCCCACACGTGGTCC
EGFP-Myc-SIVA1-48-F	GAGGACTTGTCCGGTTCCTGGGATGAAGGCTGT
SIVA1-114-HA-R	ATAAGGGTAGGAACCGGAACCGGCAATGGA
EGFP-Myc-SIVA1-107-F	GAGGACTTGTCCGGTTCCTCTGGGGTAGCGTCC
SIVA1-175-HA-R	GACATCATAAGGGTAGGAACCGGAACCGGTCTC
RFP-C2ORF68-FL-F	ACTCCACCGGCGCCCCTATGGAGGCGGGGCC
C2ORF68-FL-Flag-R	CGTCATCGTCTTTGTAGTCCTCGTGTTGGCTC

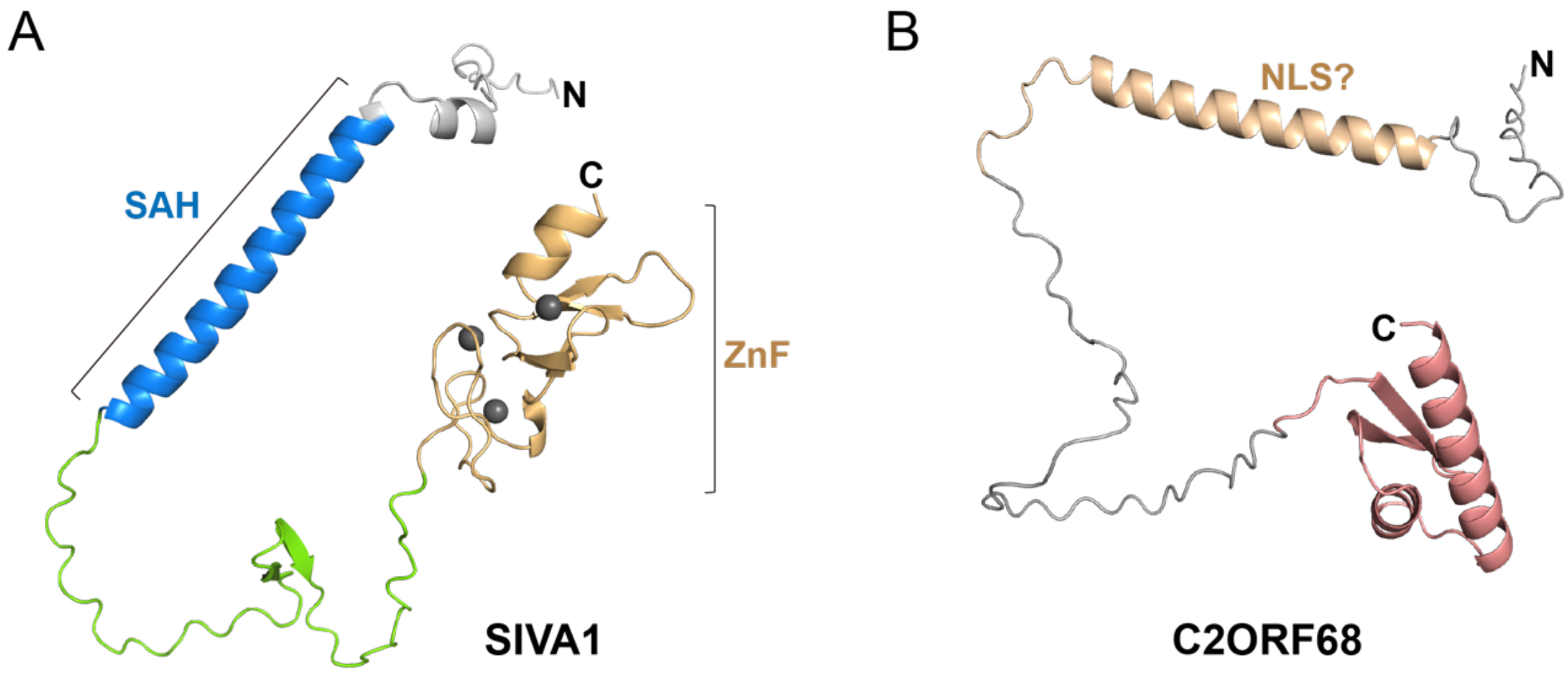

Supplementary Fig 1.

A Schematic diagram of the full-length structure of SIVA1 predicted by AlphaFold 3.

B Schematic diagram of the full-length structure of C2ORF68 predicted by AlphaFold 3.

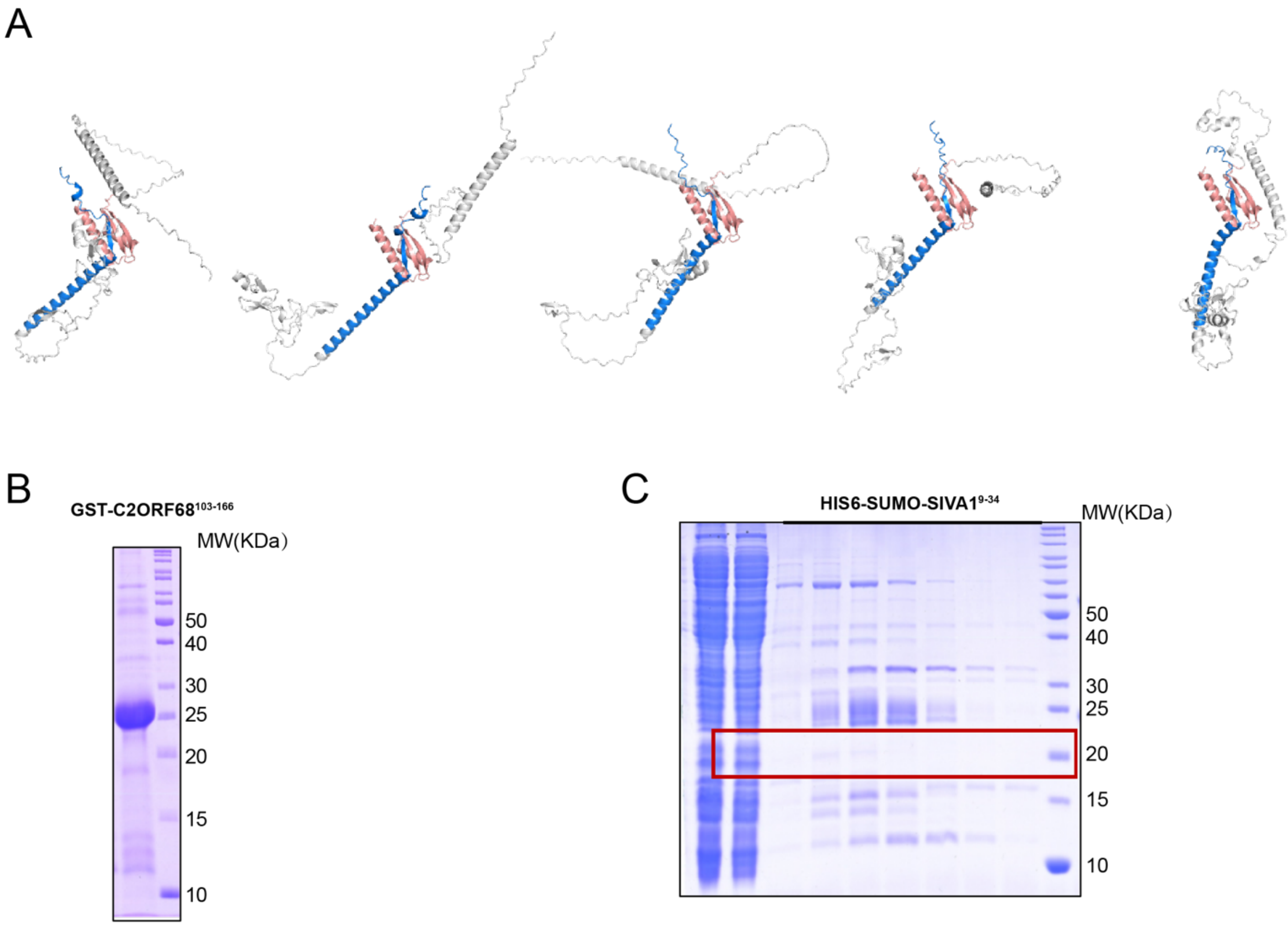

Supplementary Fig 2

A The top five ranked models of the SIVA1/C2ORF68 complex predicted by AlphaFold 3. The UPF0561 domain of C2ORF68 and SIVA1 are shown in red and blue, respectively, with the loop regions in grey. **B** SDS-PAGE and Coomassie blue staining of GST-C2ORF68$^{103\text{-}166}$. **C** SDS-PAGE and Coomassie blue staining of His$_6$-SUMO-SIVA1$^{9\text{-}34}$.

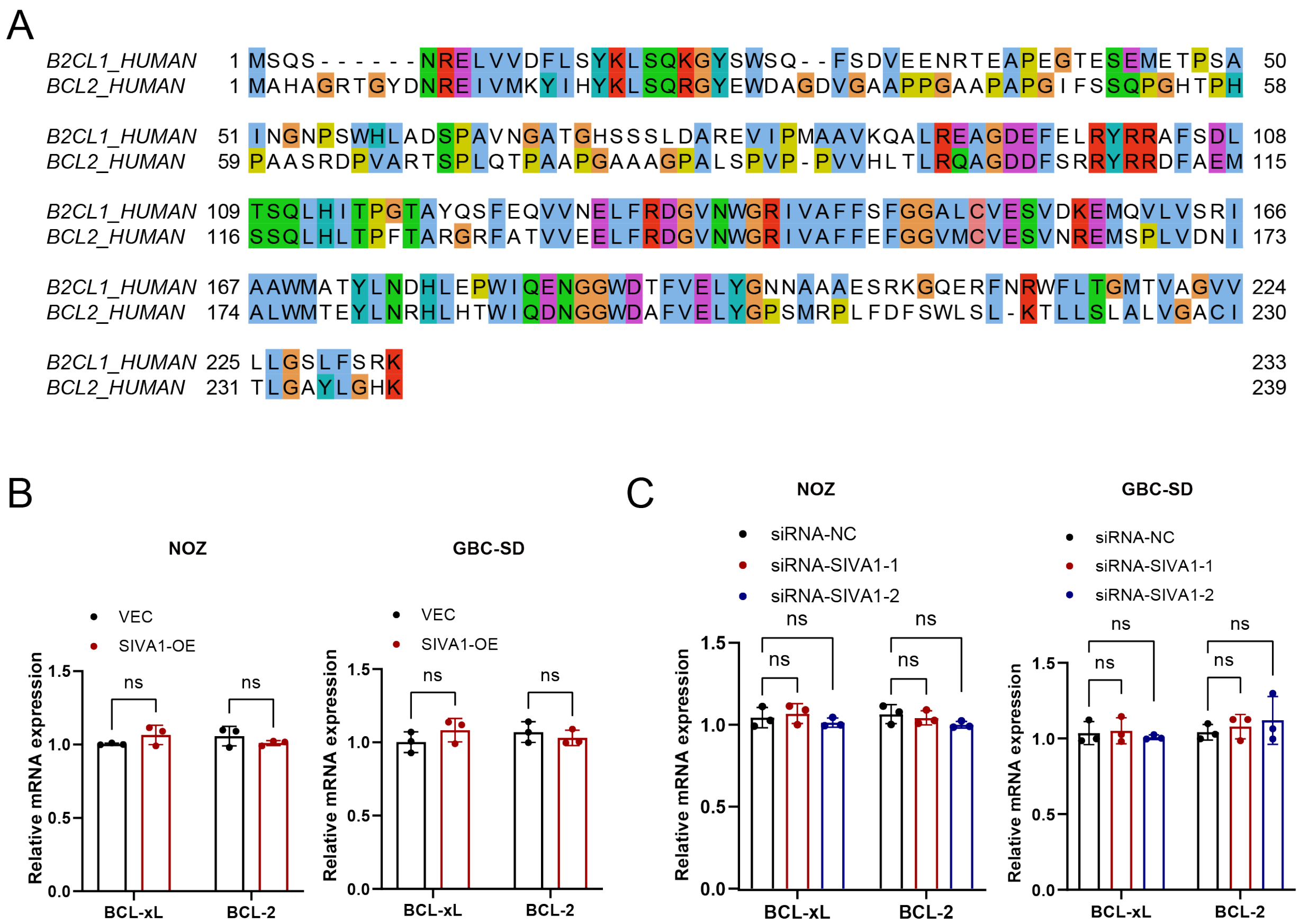

Supplementary Fig 3

A The amino acid sequence alignment of BCL-2 and BCL-xL (also known as B2CL1). **B** qRT-PCR analysis of the effects of SIVA1 overexpression on BCL-xL and BCL-2 mRNA levels in NOZ and GBC-SD cells. **C** qRT-PCR analysis of the effects of SIVA1 knockdown on BCL-xL and BCL-2 mRNA levels in NOZ and GBC-SD cells.

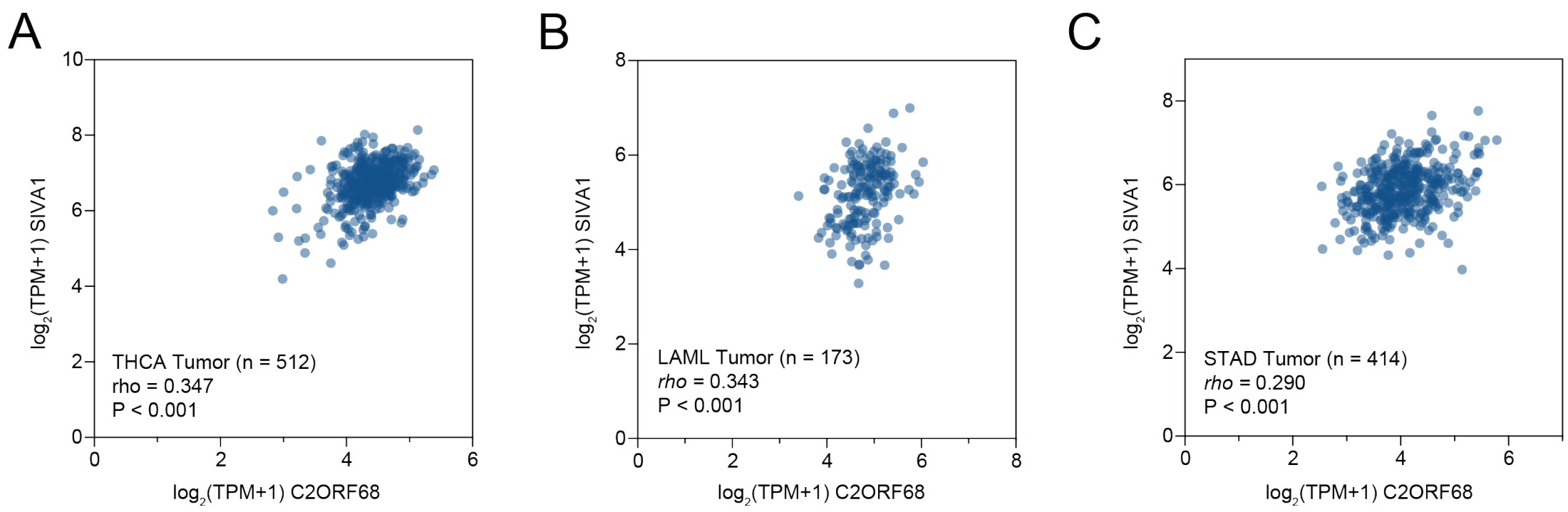

Supplementary Fig 4

Correlation analysis of endogenous C2ORF68 and SIVA1 expression in TCGA cancer datasets. Spearman correlation analysis of endogenous C2ORF68 and SIVA1 mRNA expression in selected TCGA cancer datasets using GEPIA3. Weak-to-modest but statistically significant positive correlations were observed in (**A**) thyroid carcinoma (THCA; *rho* = 0.347, P < 0.001), (**B**) acute myeloid leukemia (LAML; *rho* = 0.343, P < 0.001), and (**C**) stomach adenocarcinoma (STAD; *rho* = 0.290, P < 0.001).
