# Supplementary material for: C2ORF68 stabilizes SIVA1 to upregulate BCL-2 and suppress apoptosis in gallbladder cancer
Source: Cell Death Discov. 2026 Jul 30;12:413. doi: 10.1038/s41420-026-03279-5 (PMC13652811; doi:10.1038/s41420-026-03279-5)
Supplement: Supplementary file 2 [file 41420_2026_3279_MOESM2_ESM.pdf]

Fig 1E

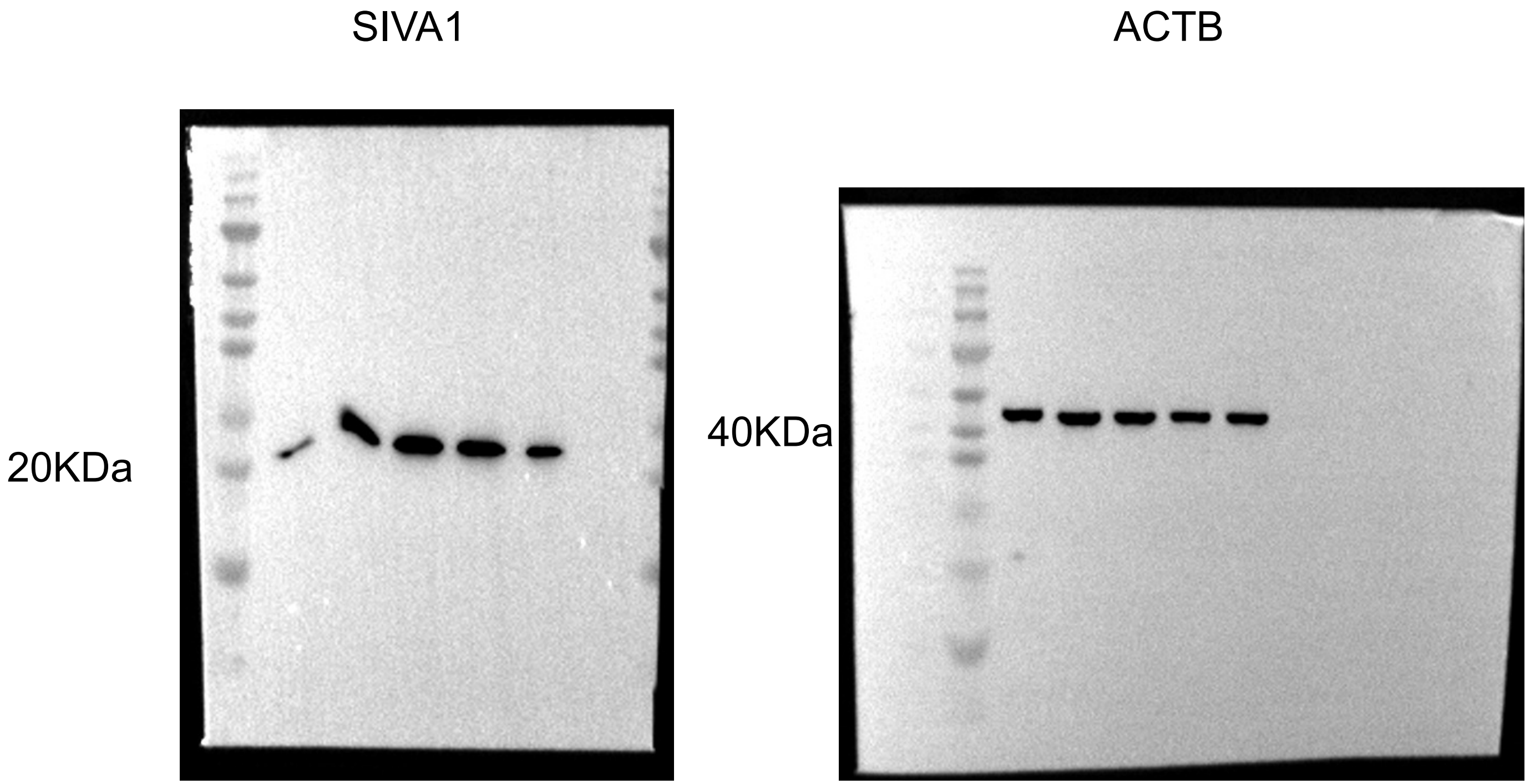

Fig 2B

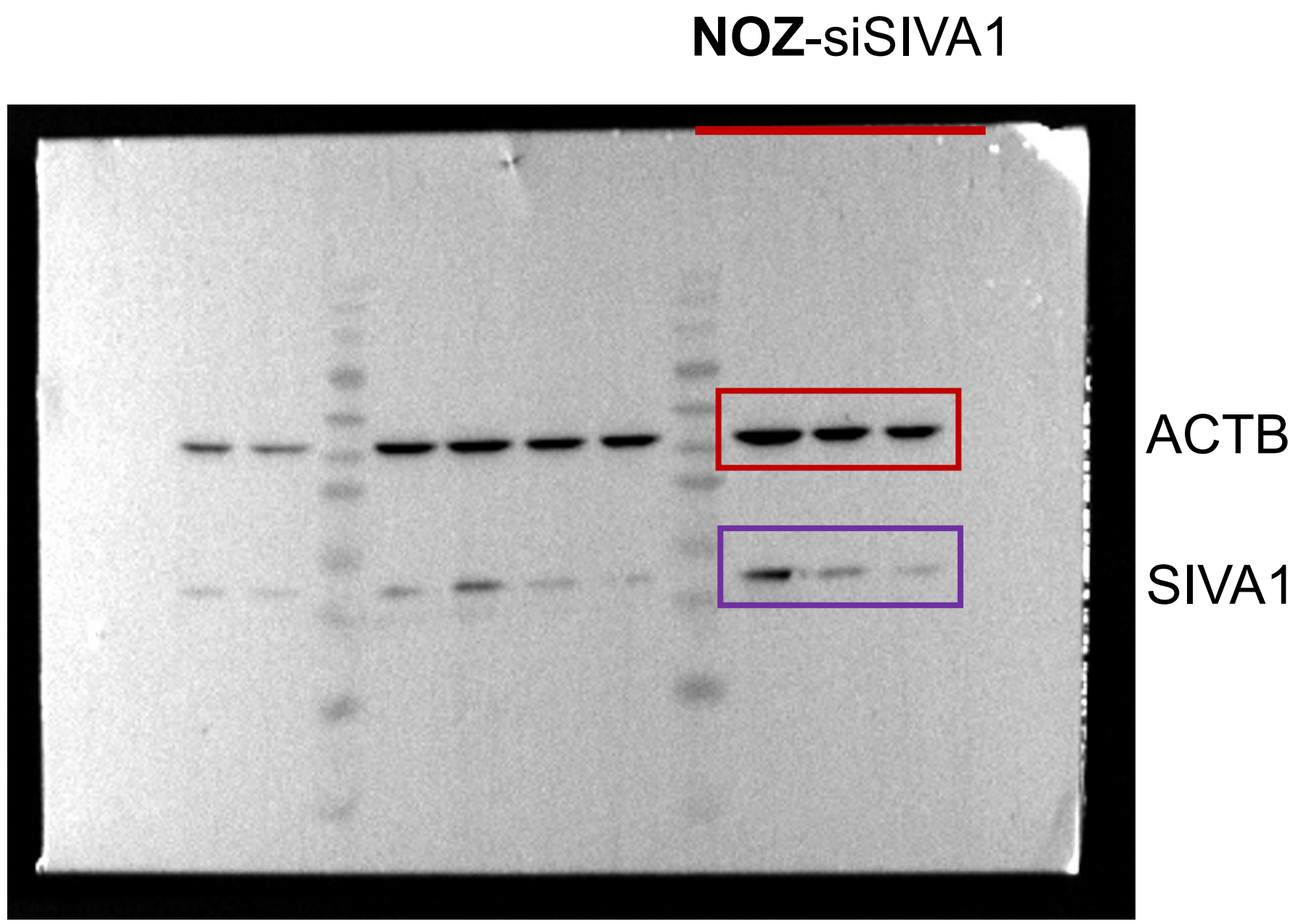

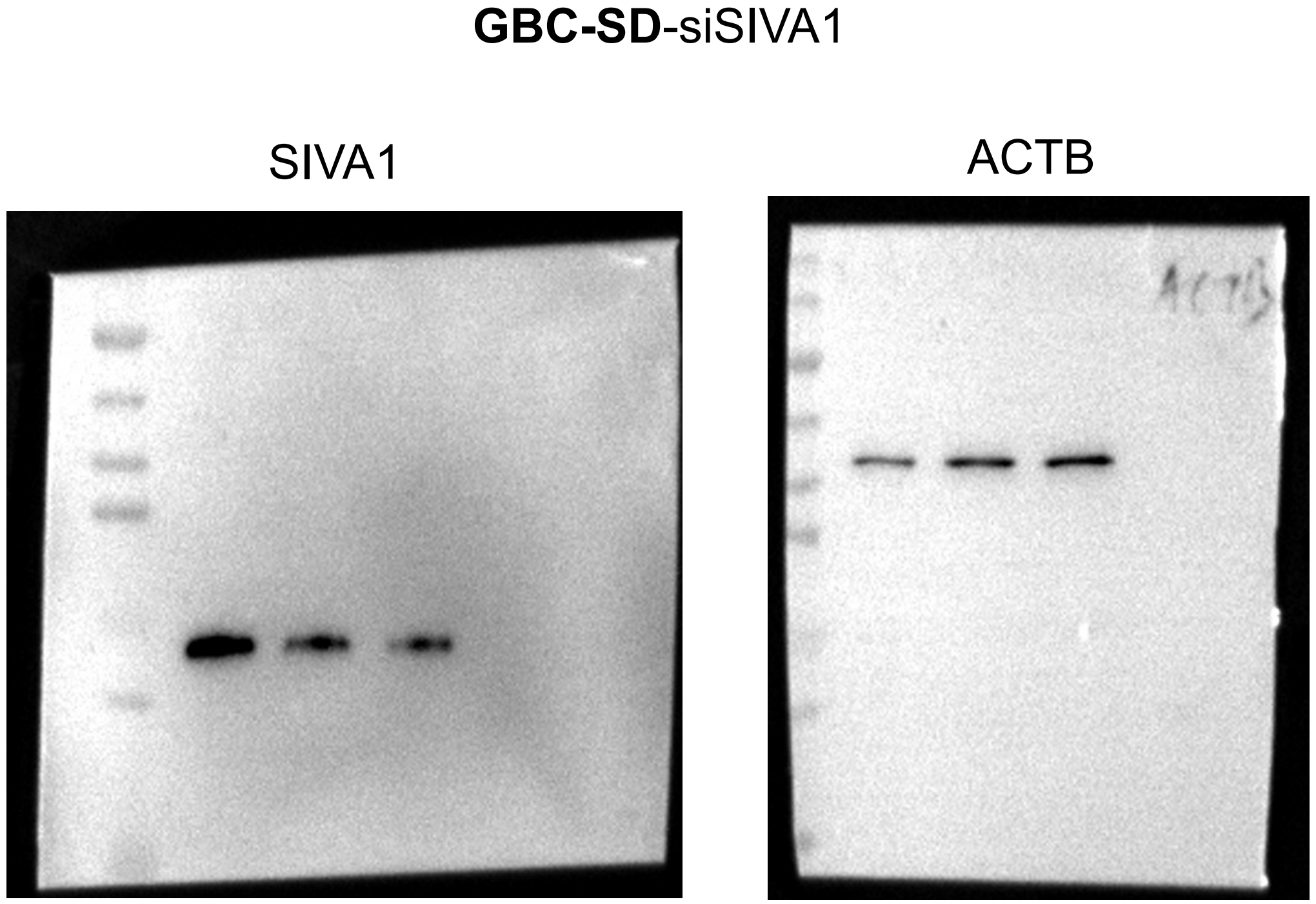

Fig 2C

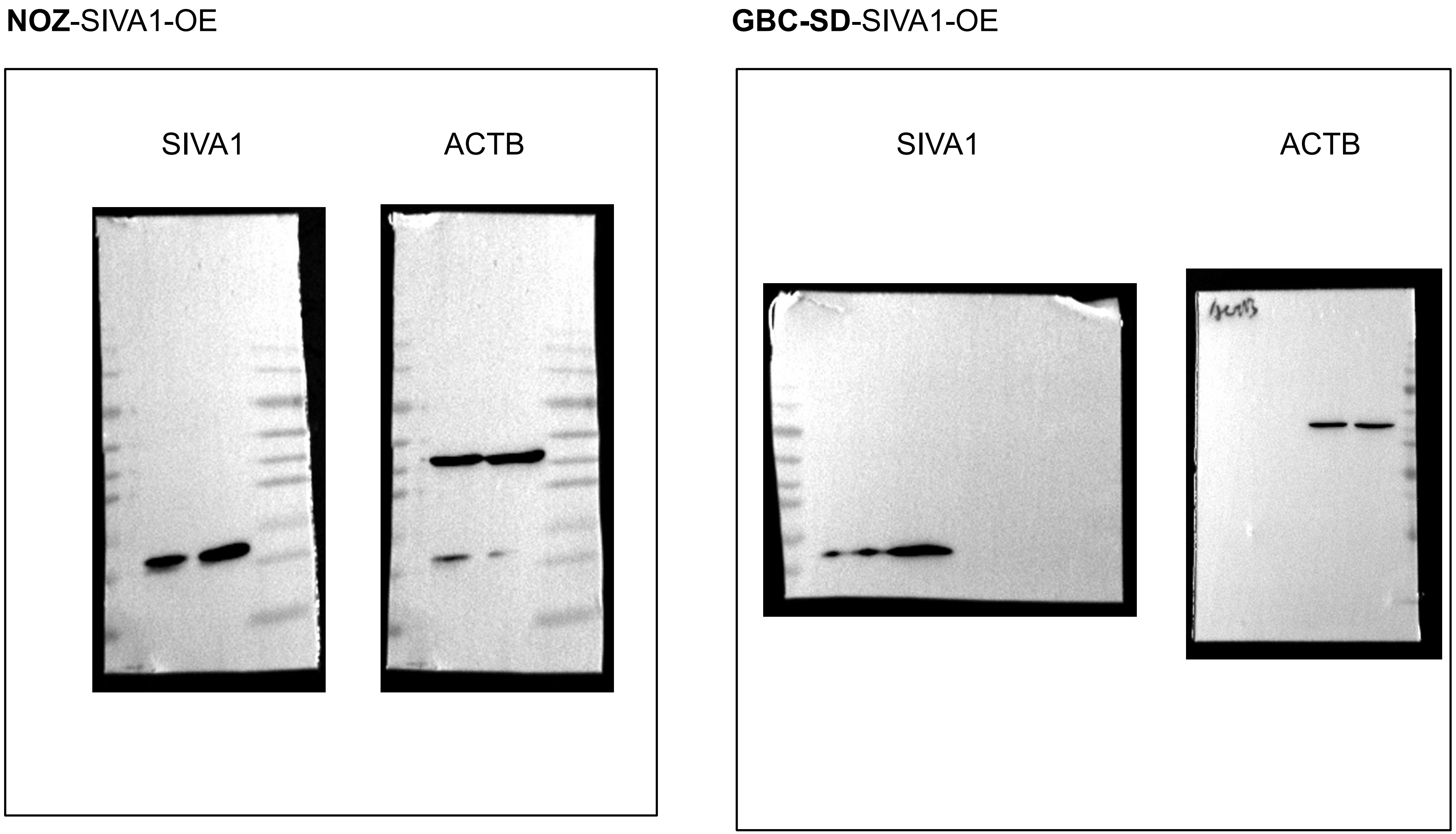

Fig 3A

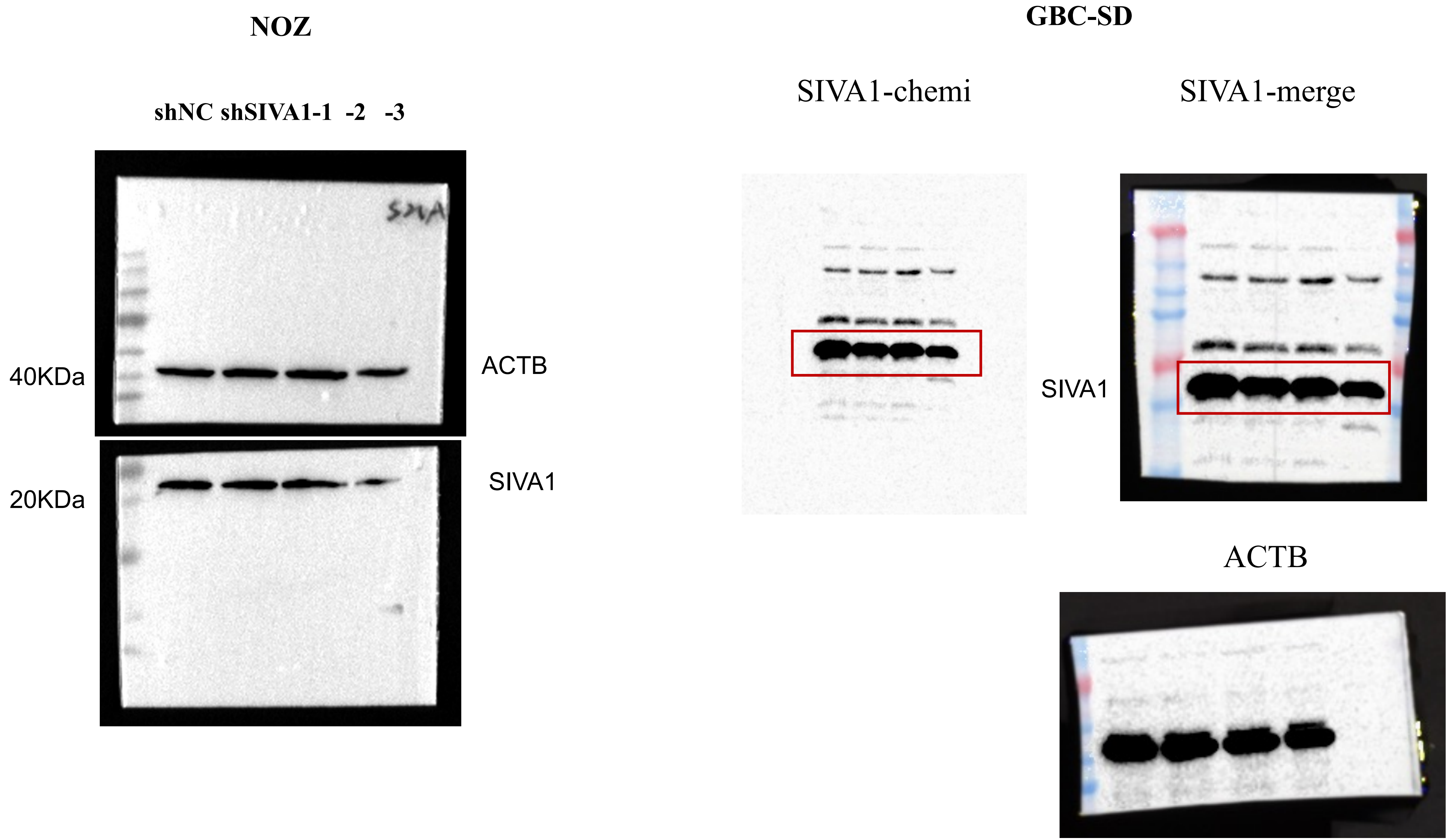
NOZ
shNC shSIVA1-1 -2 -3
40KDa
ACTB
20KDa
SIVA1
GBC-SD
SIVA1-chemi
SIVA1-merge
SIVA1
ACTB

Fig 4C HEK293T

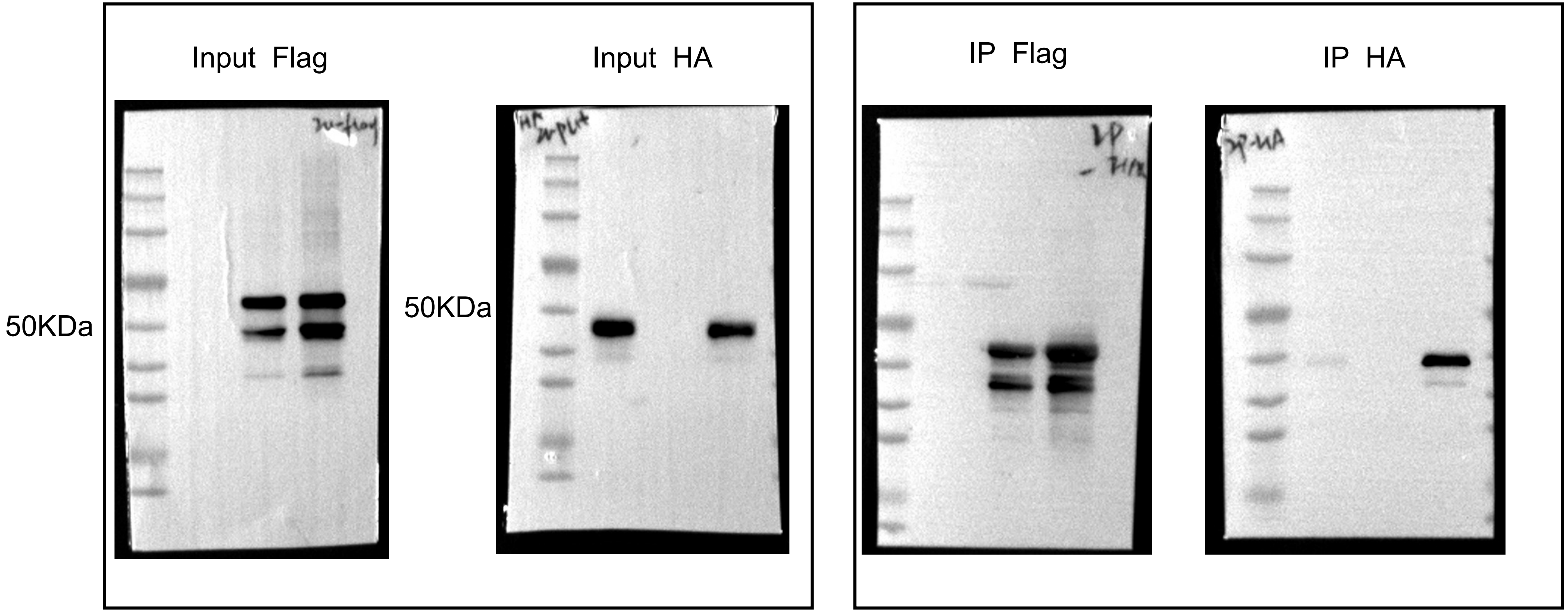

Fig 4C-GBC-SD

Flag Input Flag IP

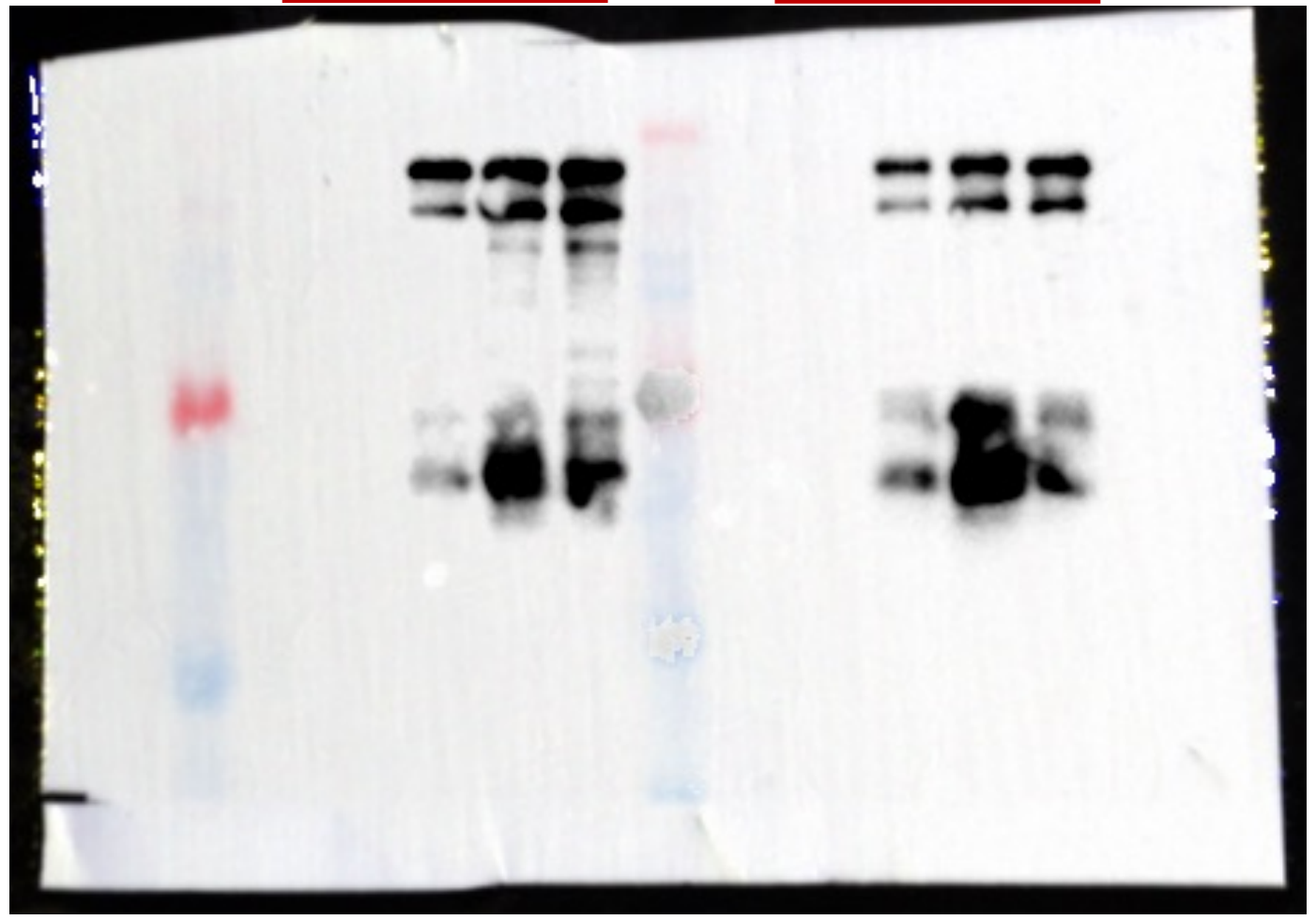

Input-HA

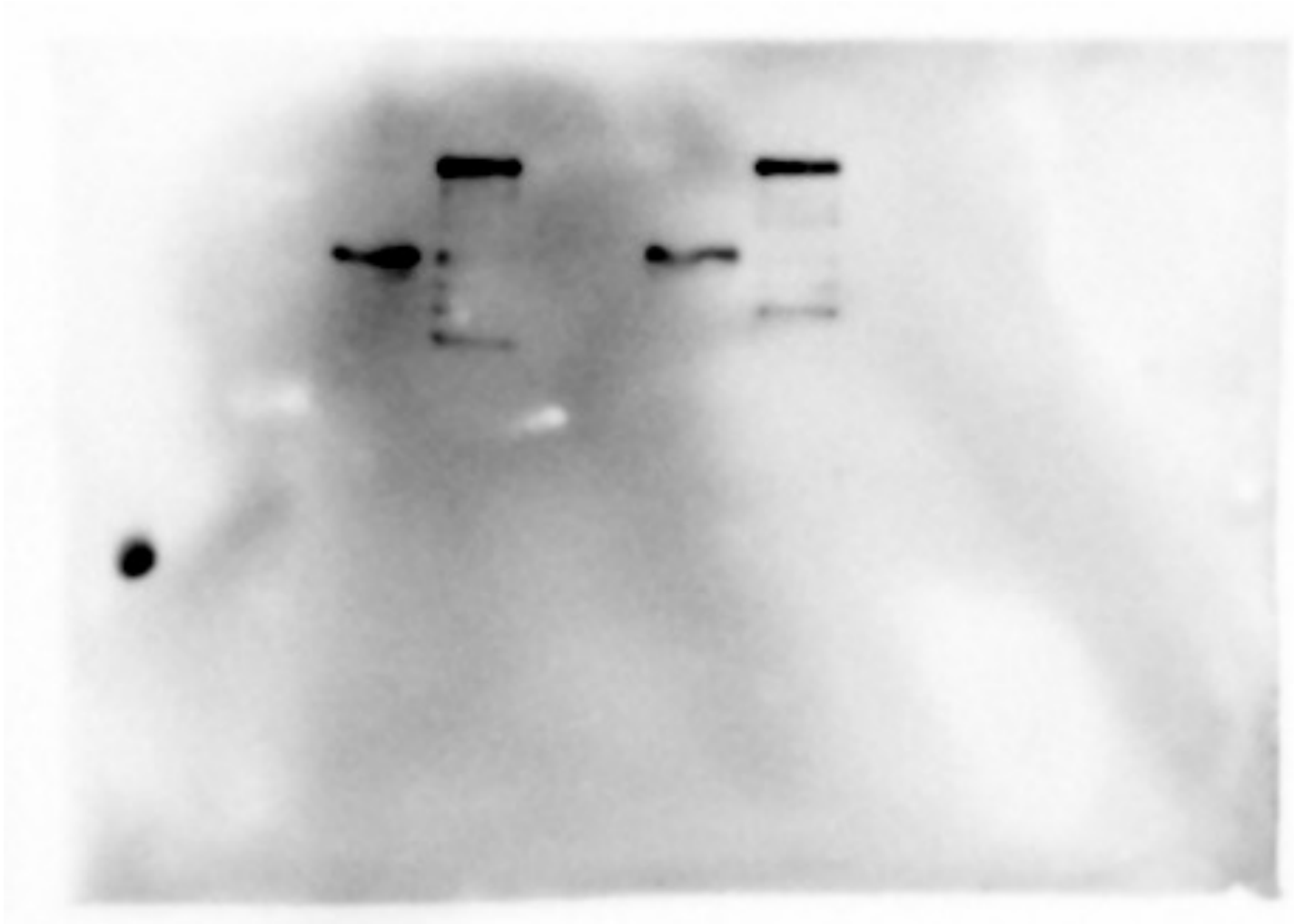

IP-HA

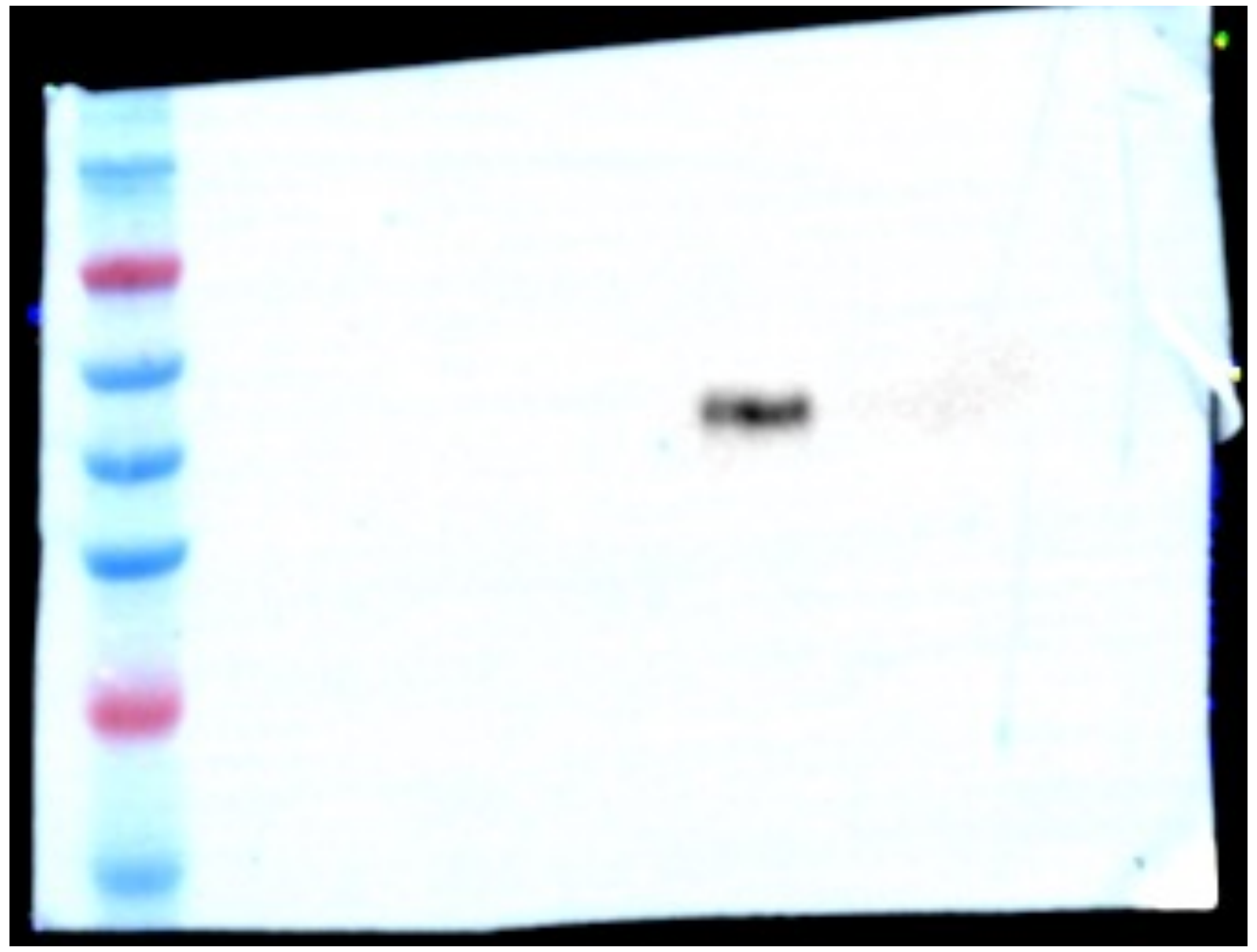

Fig 4I

Flag

Input IP

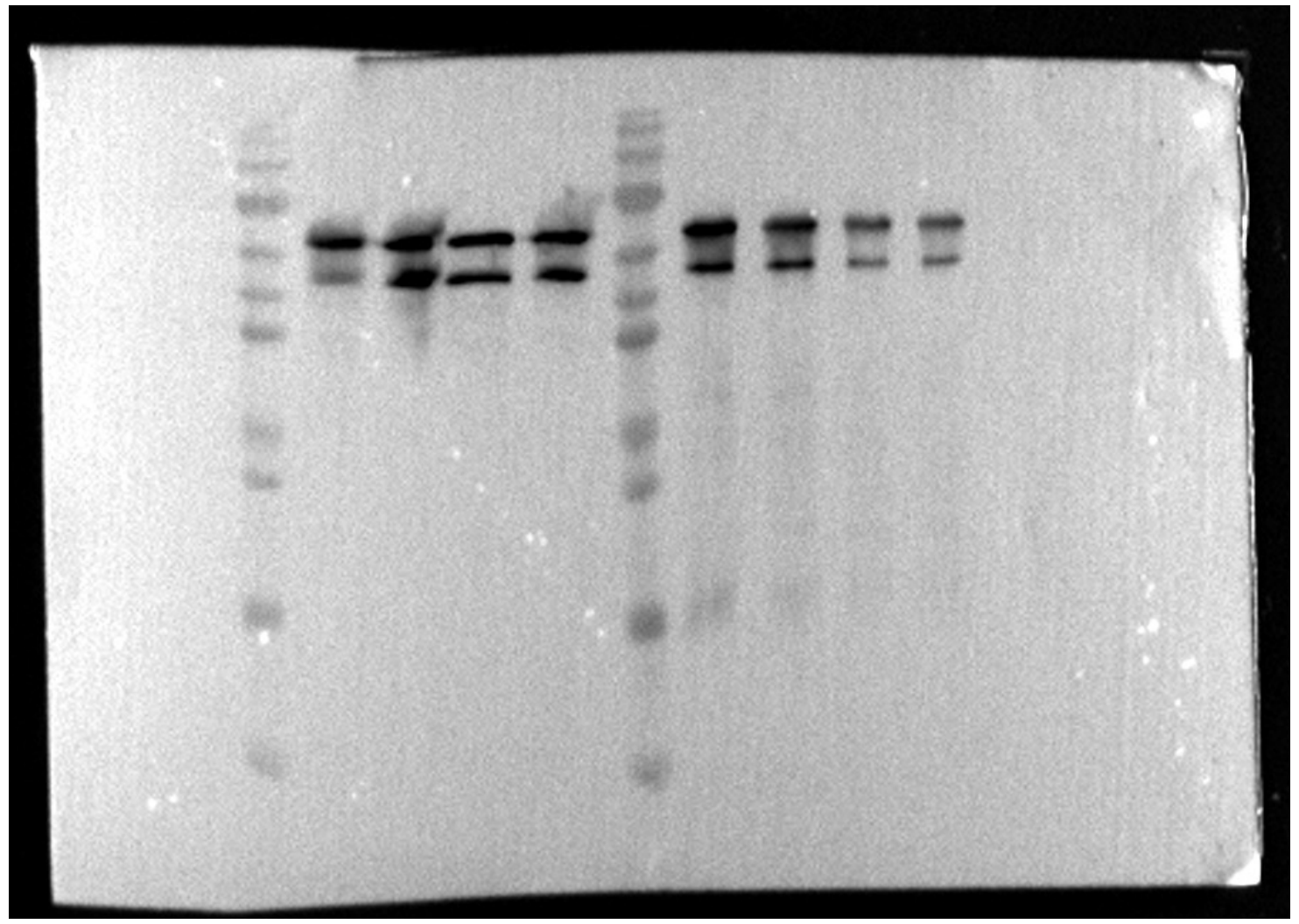

HA

Input IP

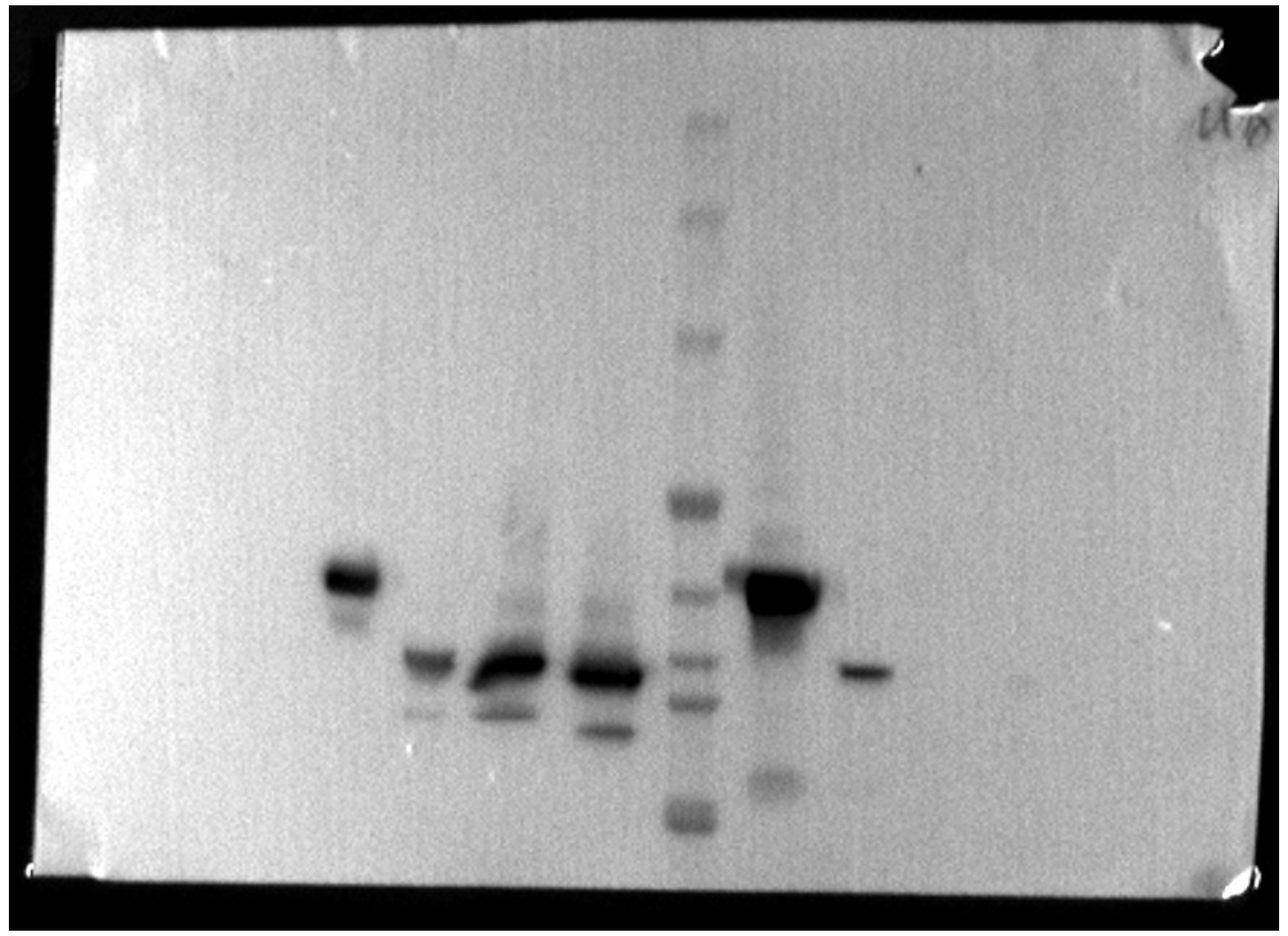

Fig 4J

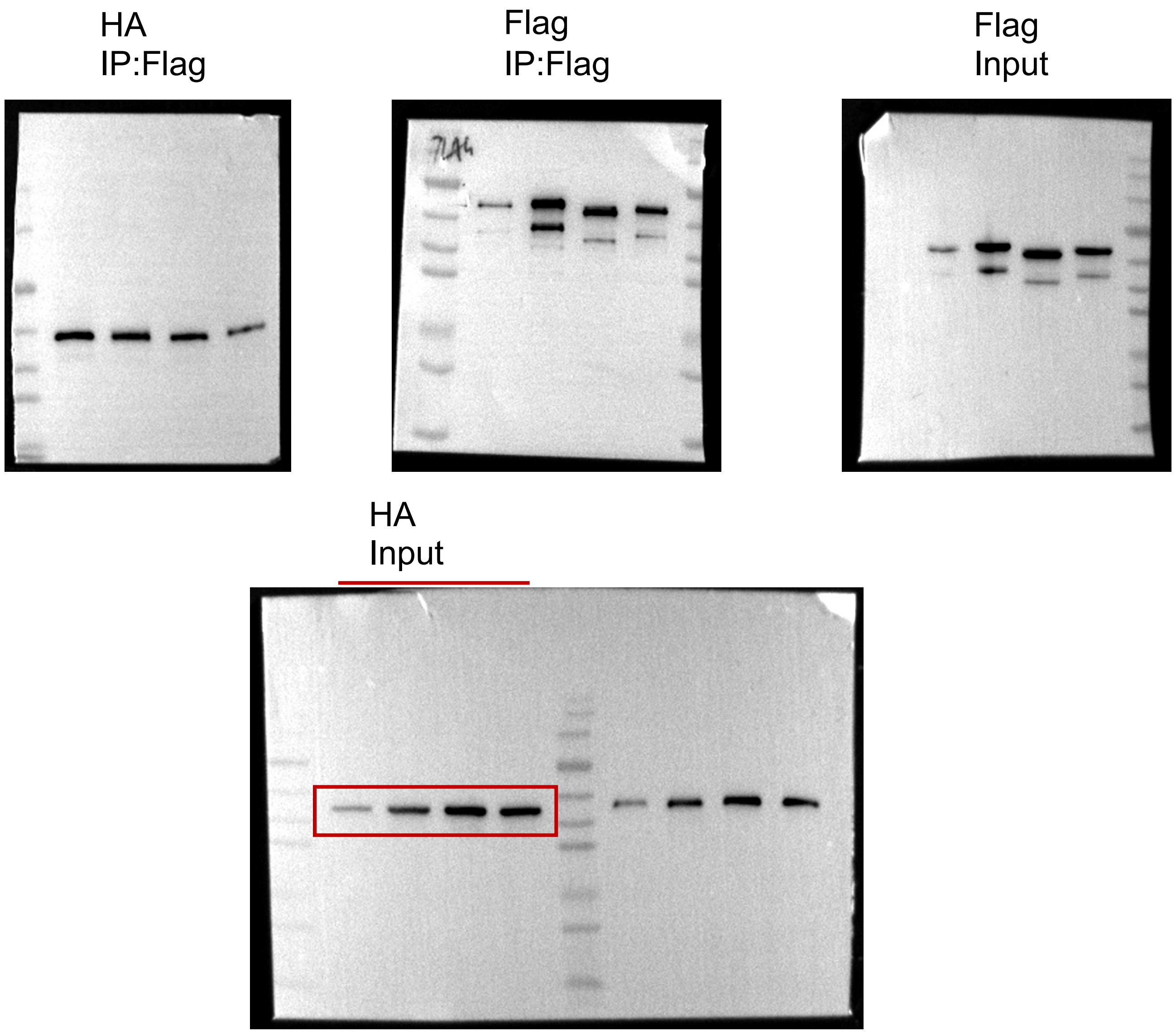

Fig 5A

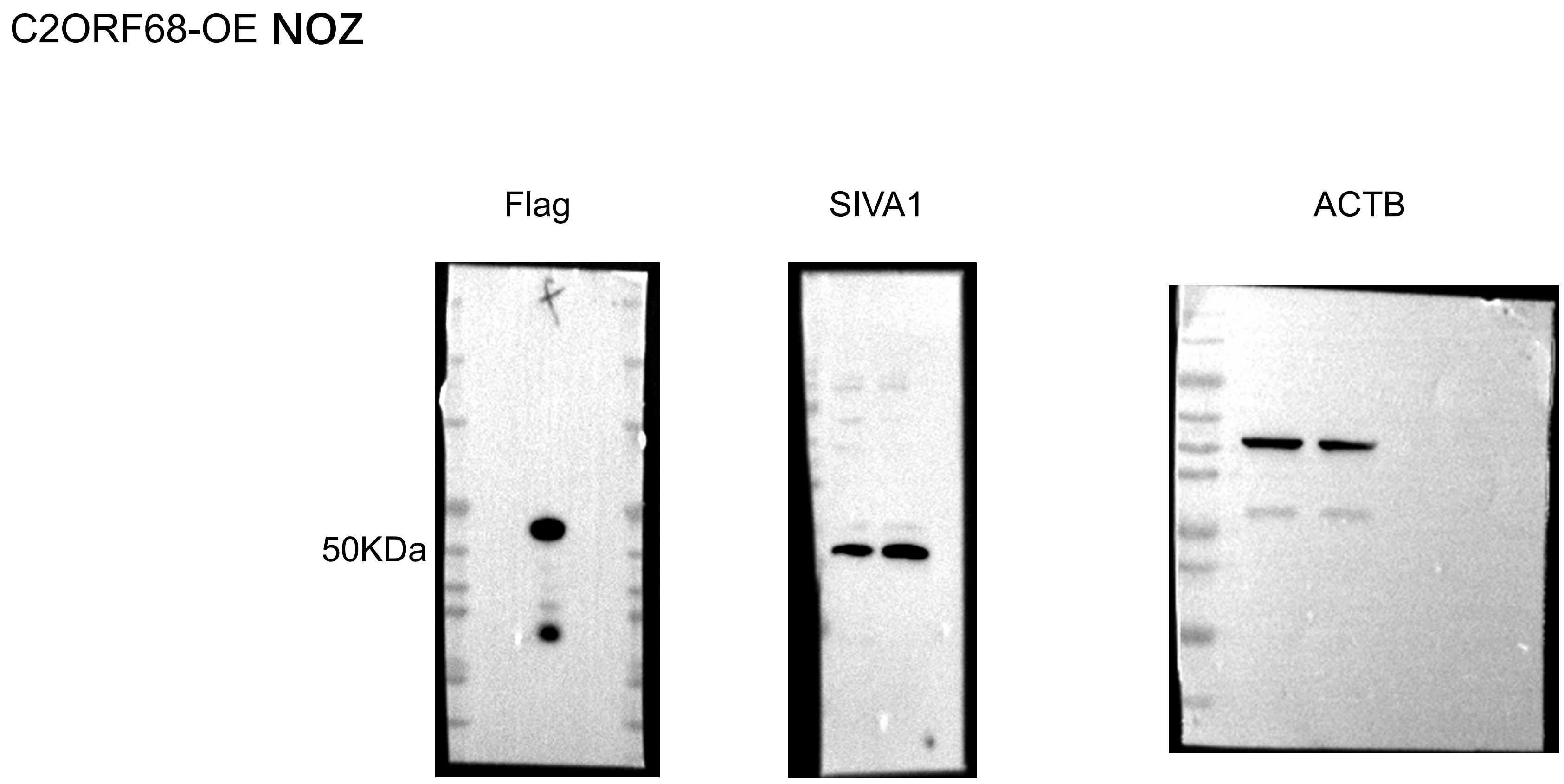

Fig 5B

C2ORF68-OE **GBC-SD**

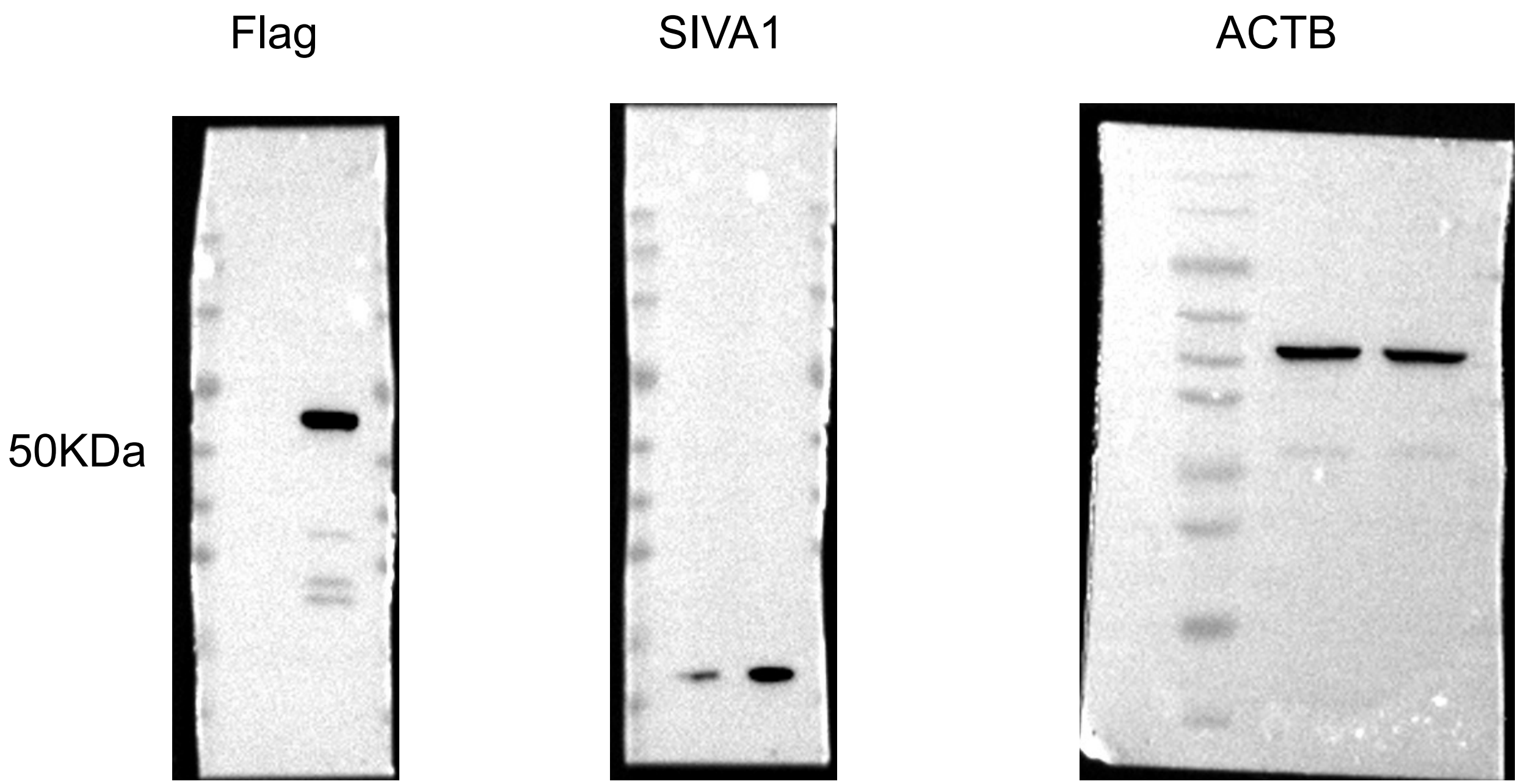

Fig 5F

NOZ

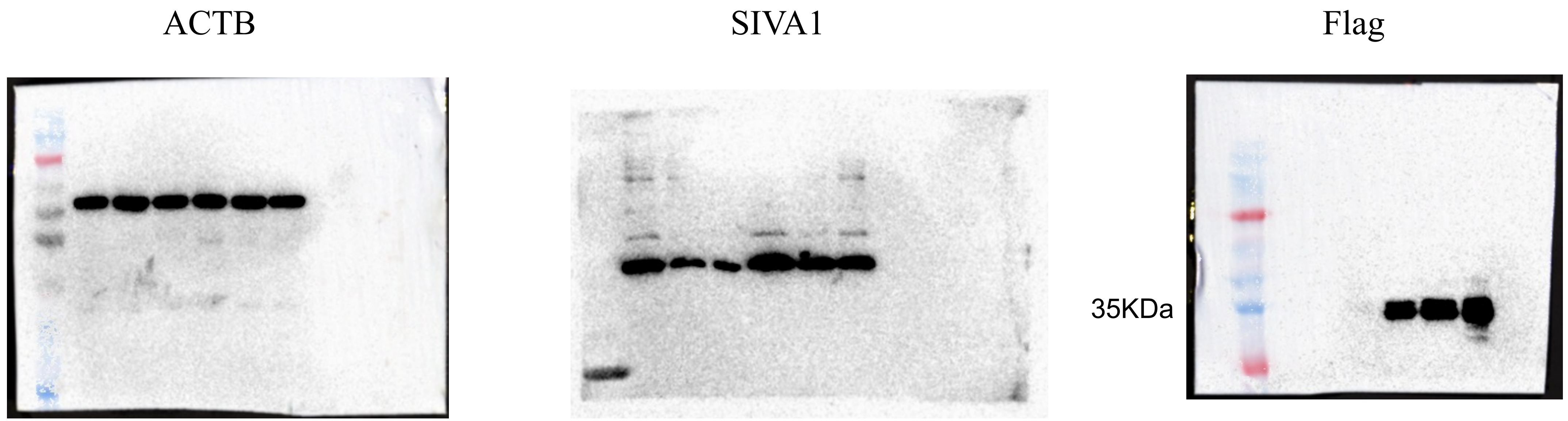

Fig 5F

GBC-SD

ACTB

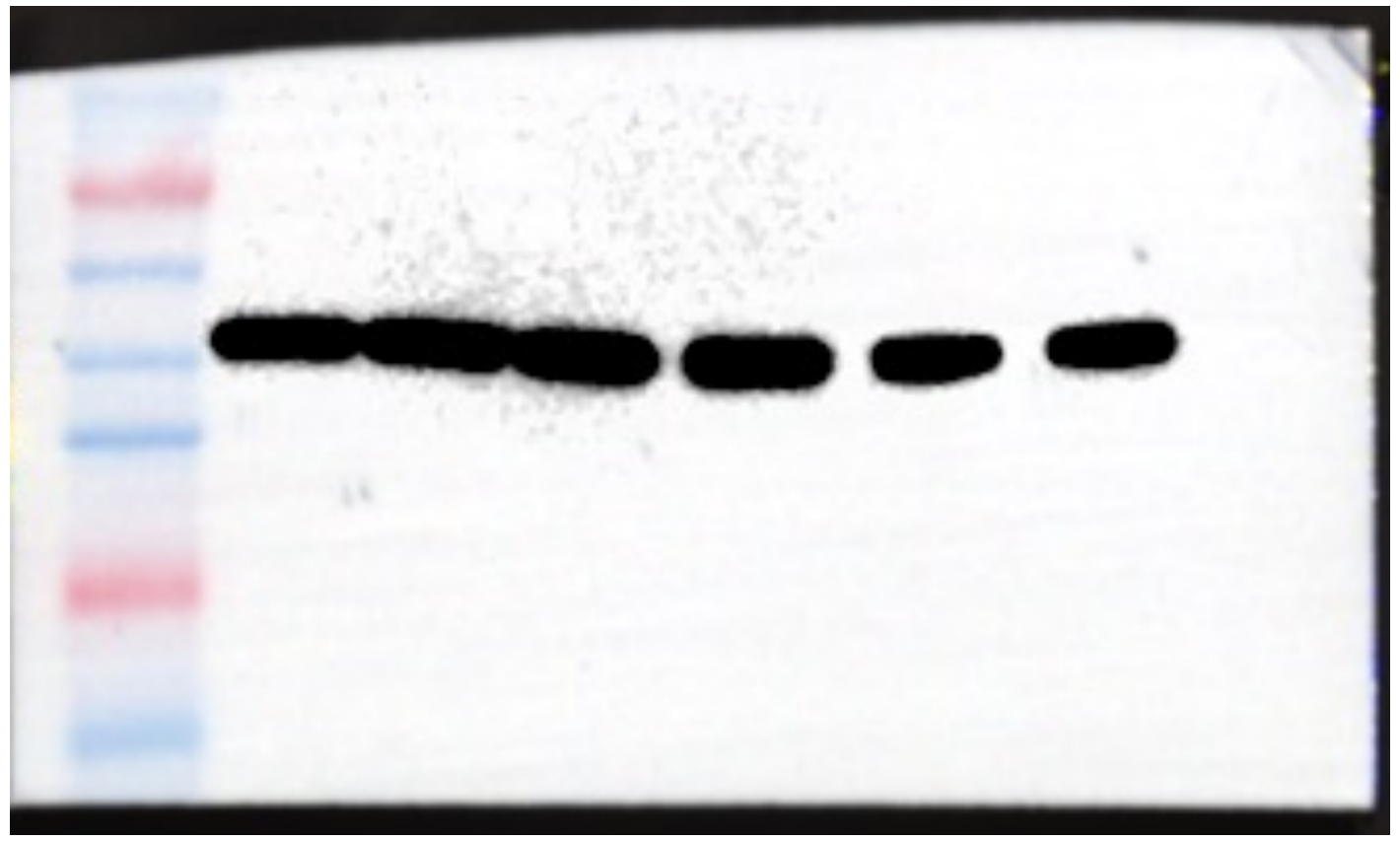

SIVA1

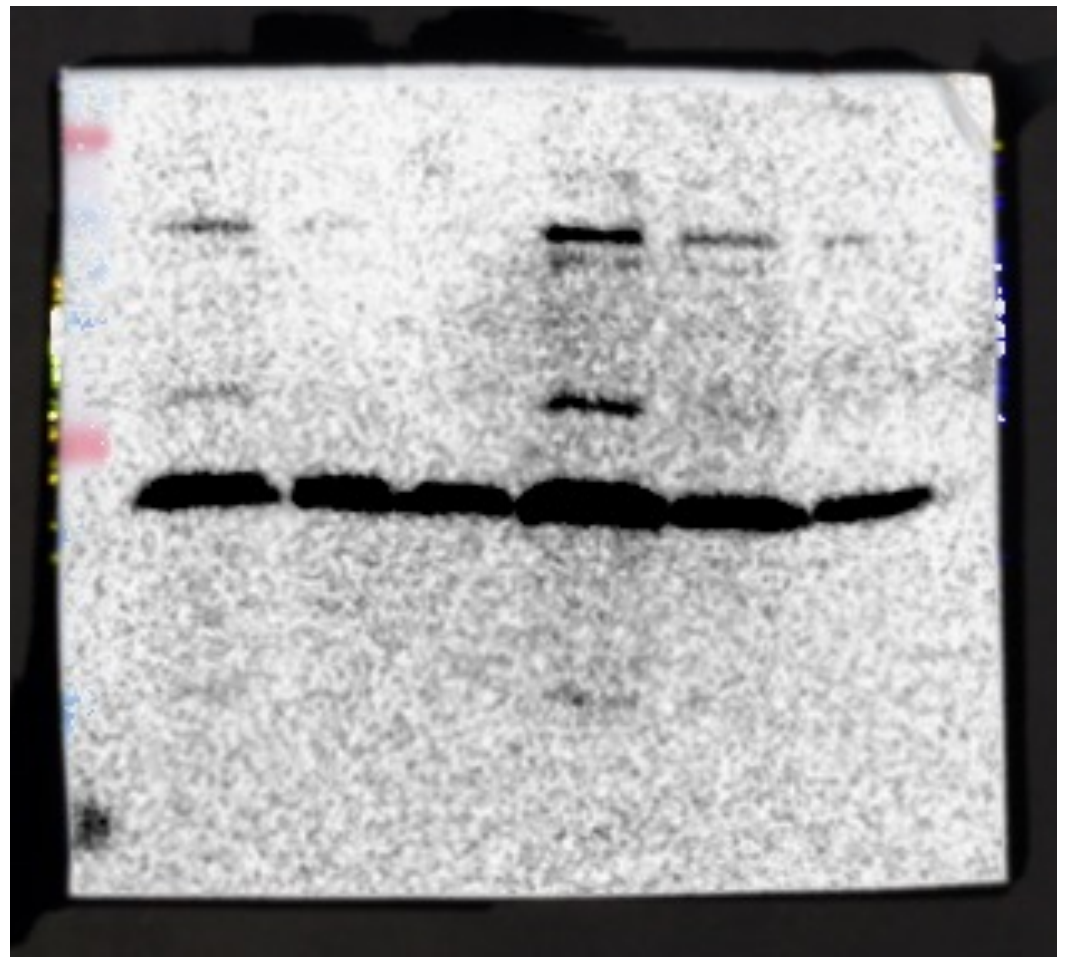

Flag

35KDa

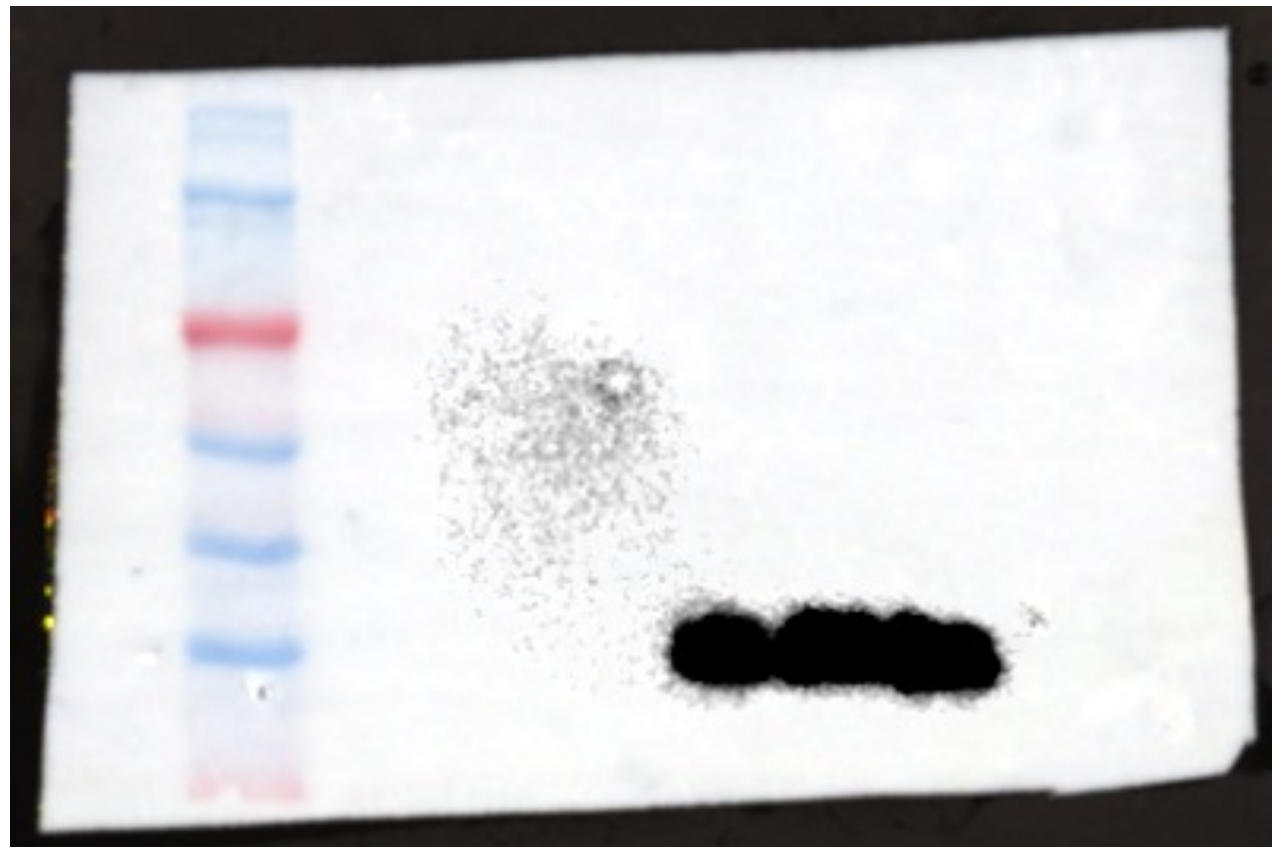

Fig 6A

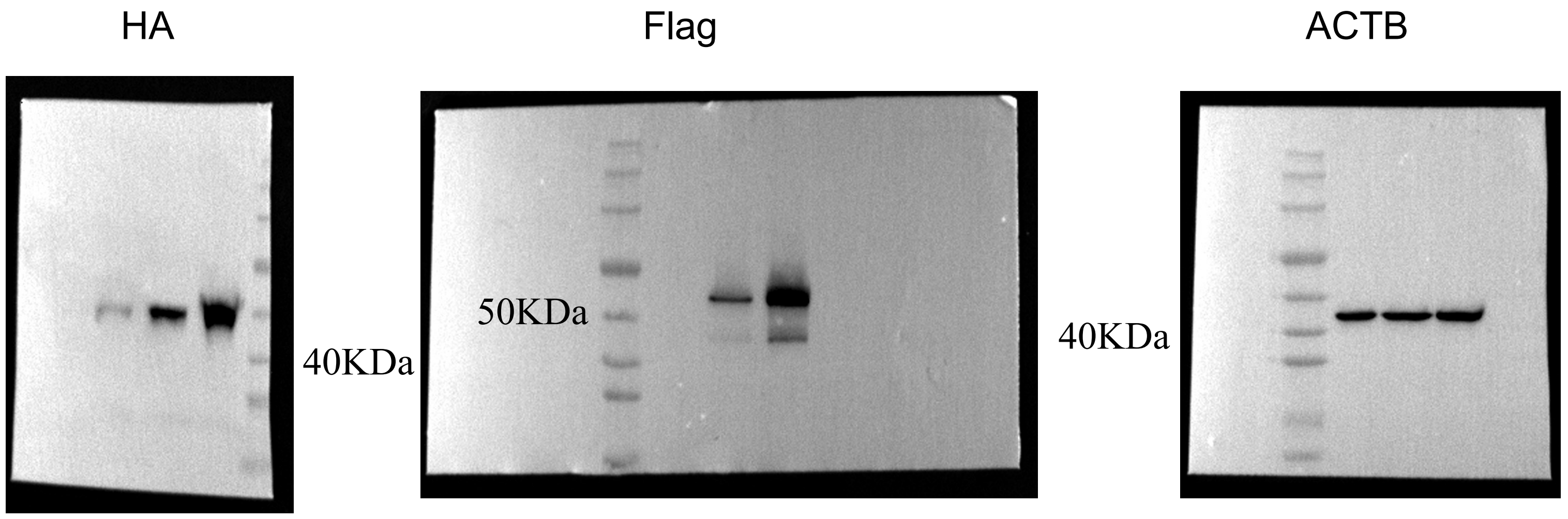

Fig 6B

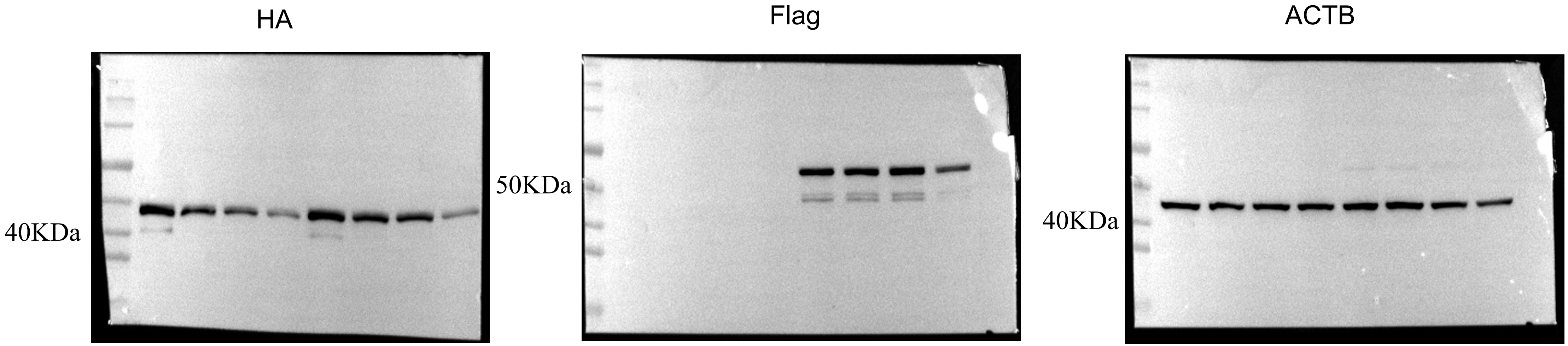

Fig 6D

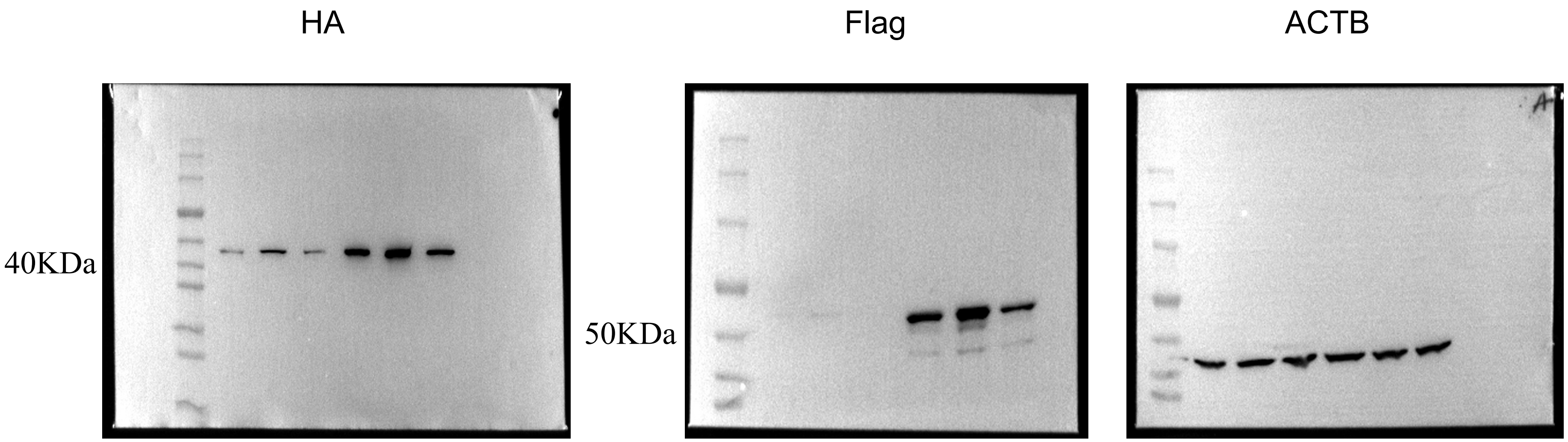

Fig 6E

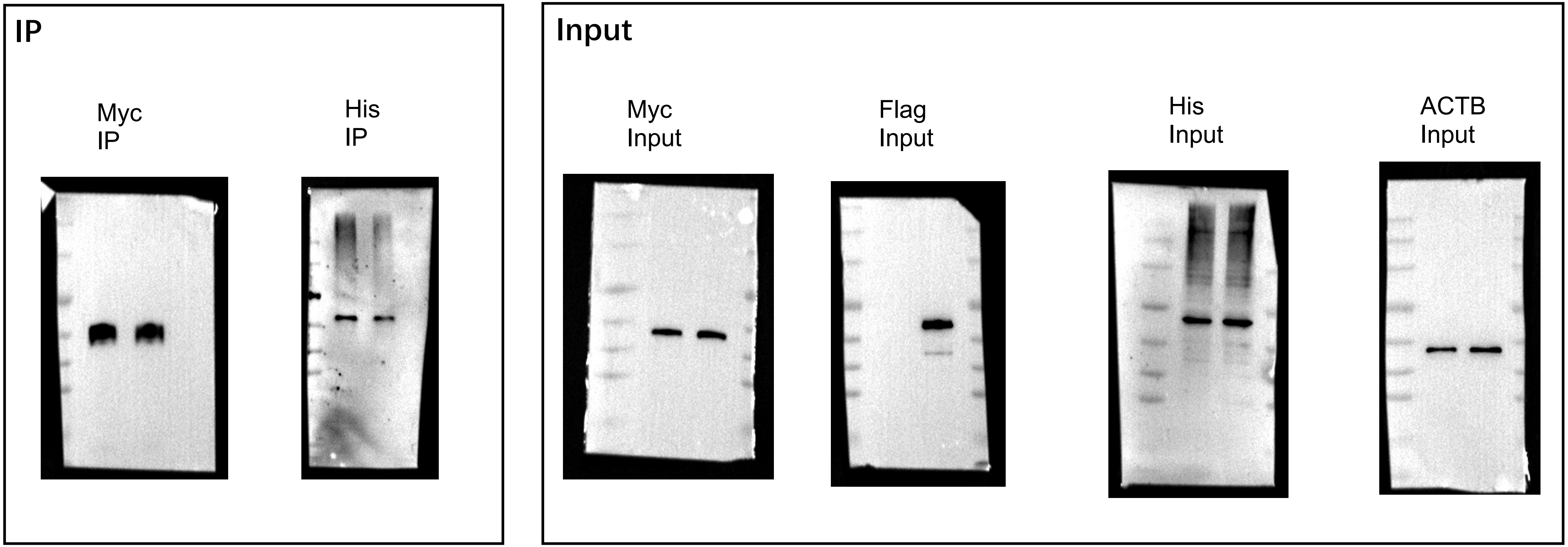
IP
Myc
IP
His
IP
Input
Myc
Input
Flag
Input
His
Input
ACTB
Input

Fig 7C

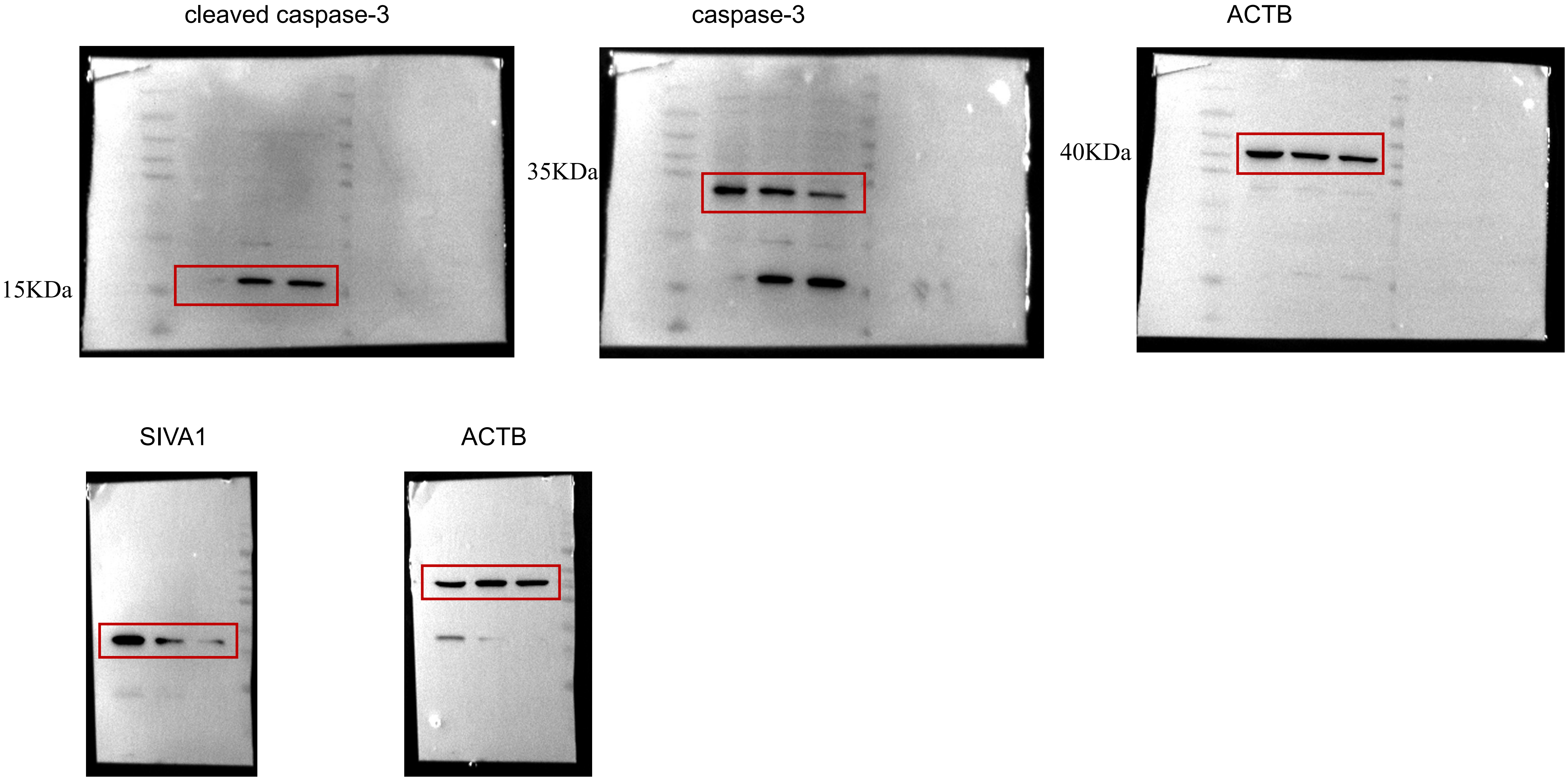

cleaved caspase-3
caspase-3
ACTB
15KDa
35KDa
40KDa
SIVA1
ACTB

Fig 8A

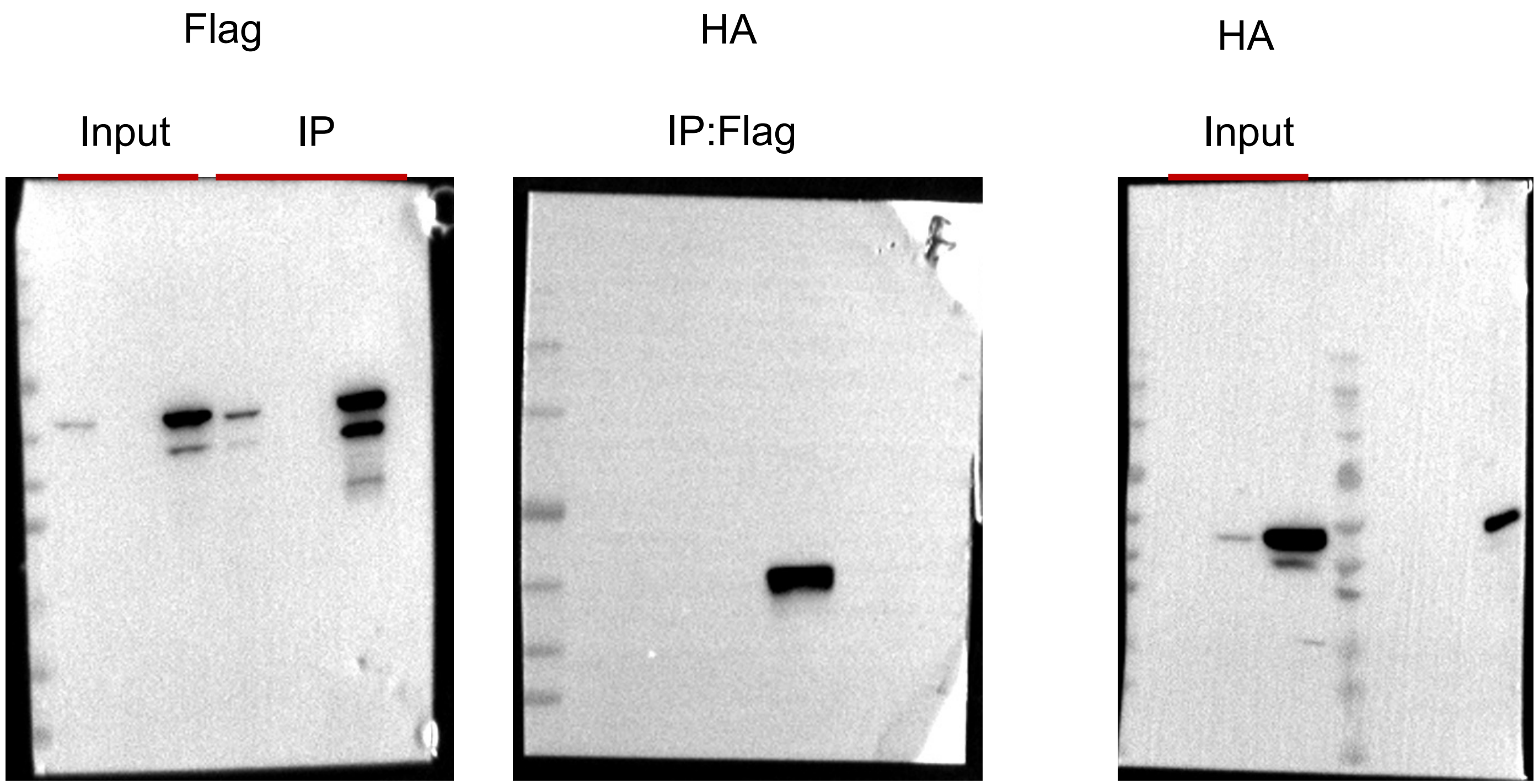

Fig 8B

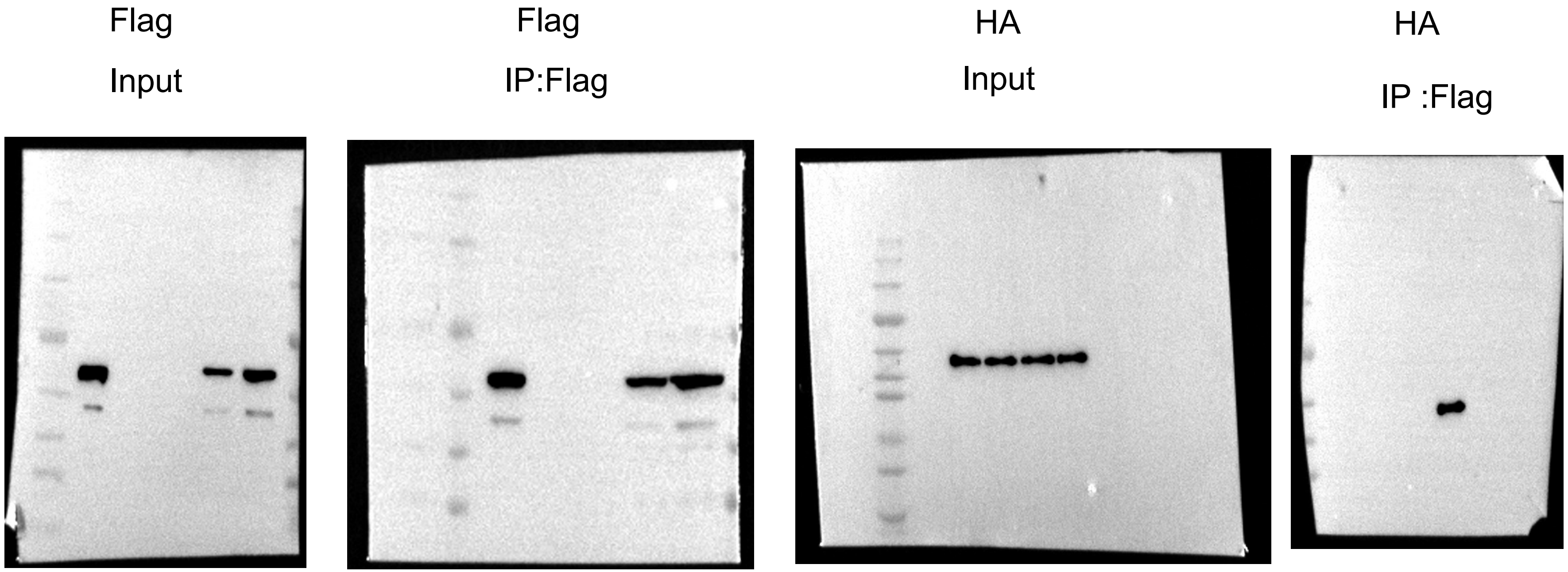

Fig 8C

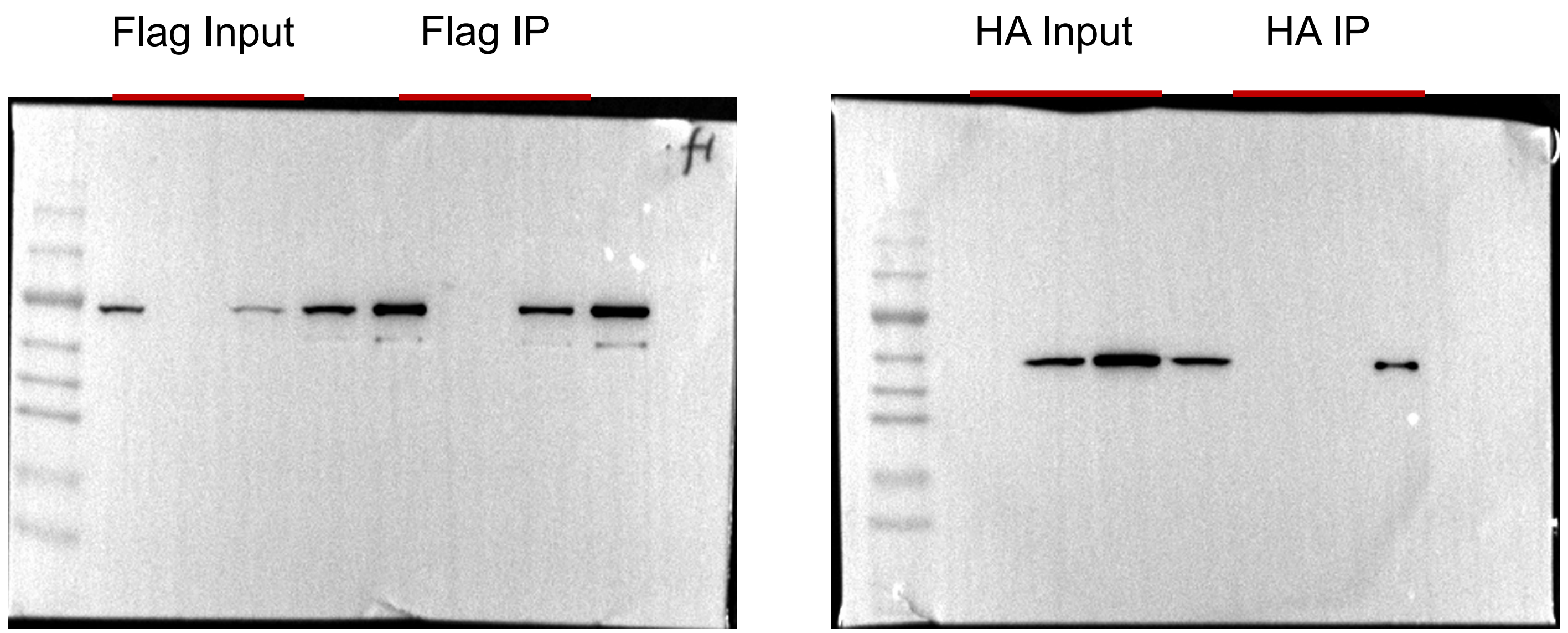

Fig 8D

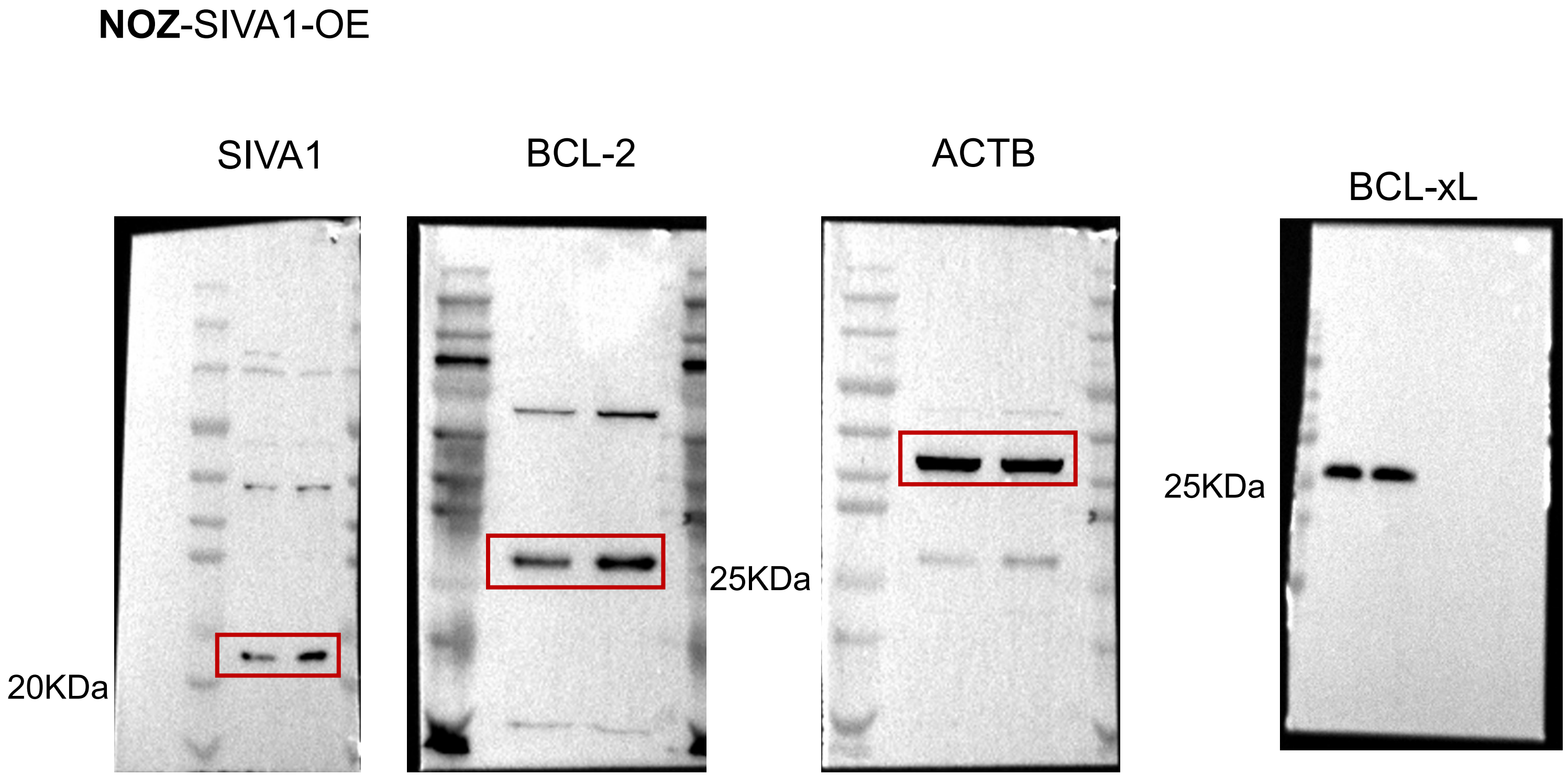

Fig 8D

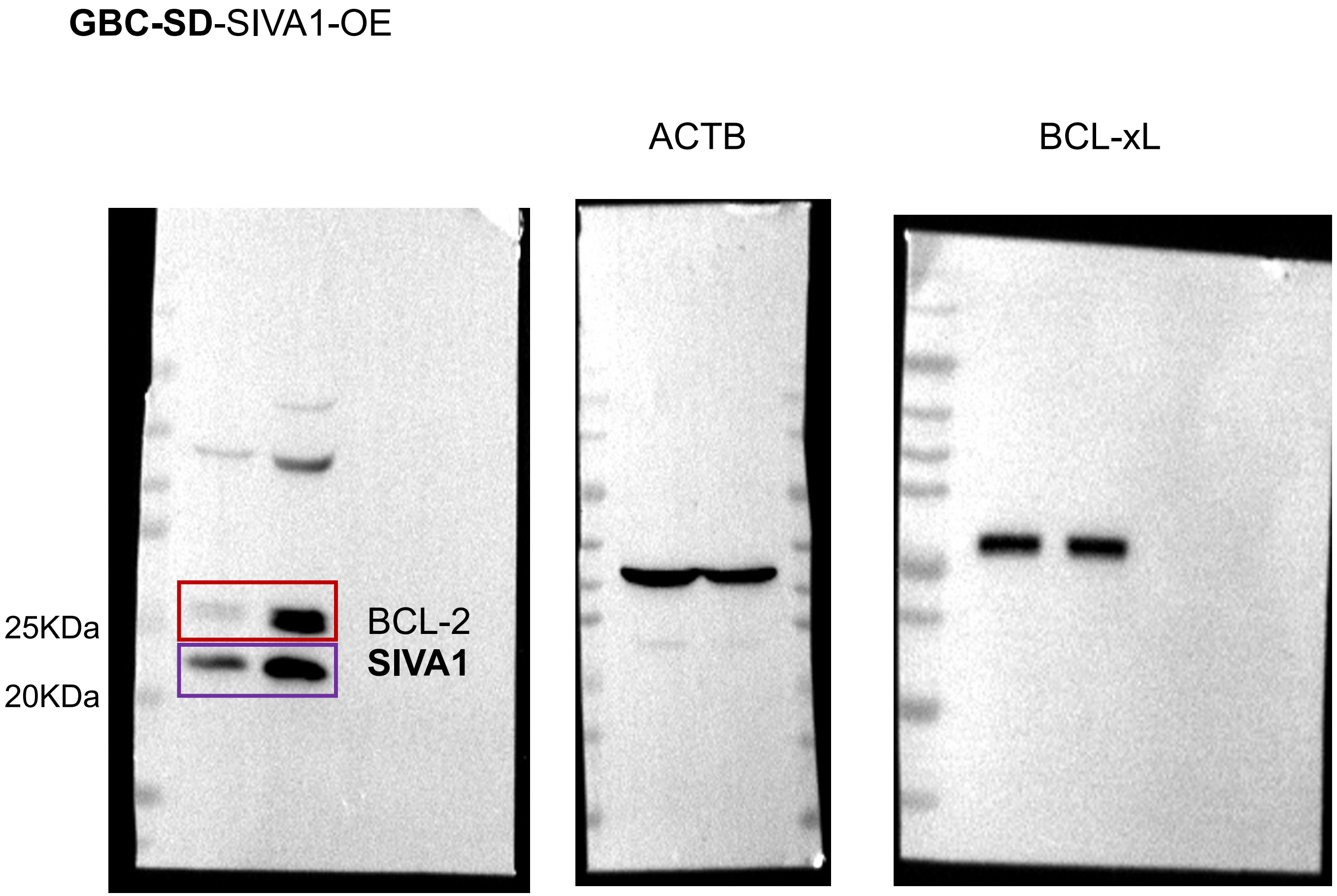

Fig 8E

NOZ-siSIVA1

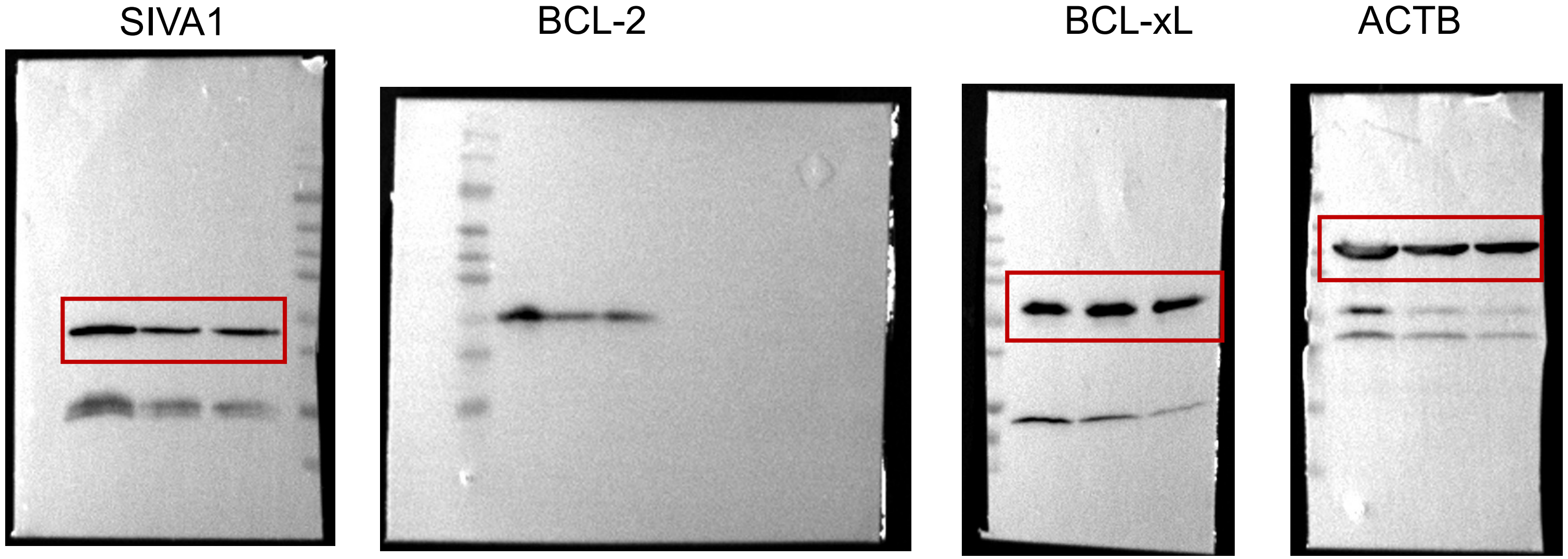

Fig 8E

GBC-SD-siSIVA1

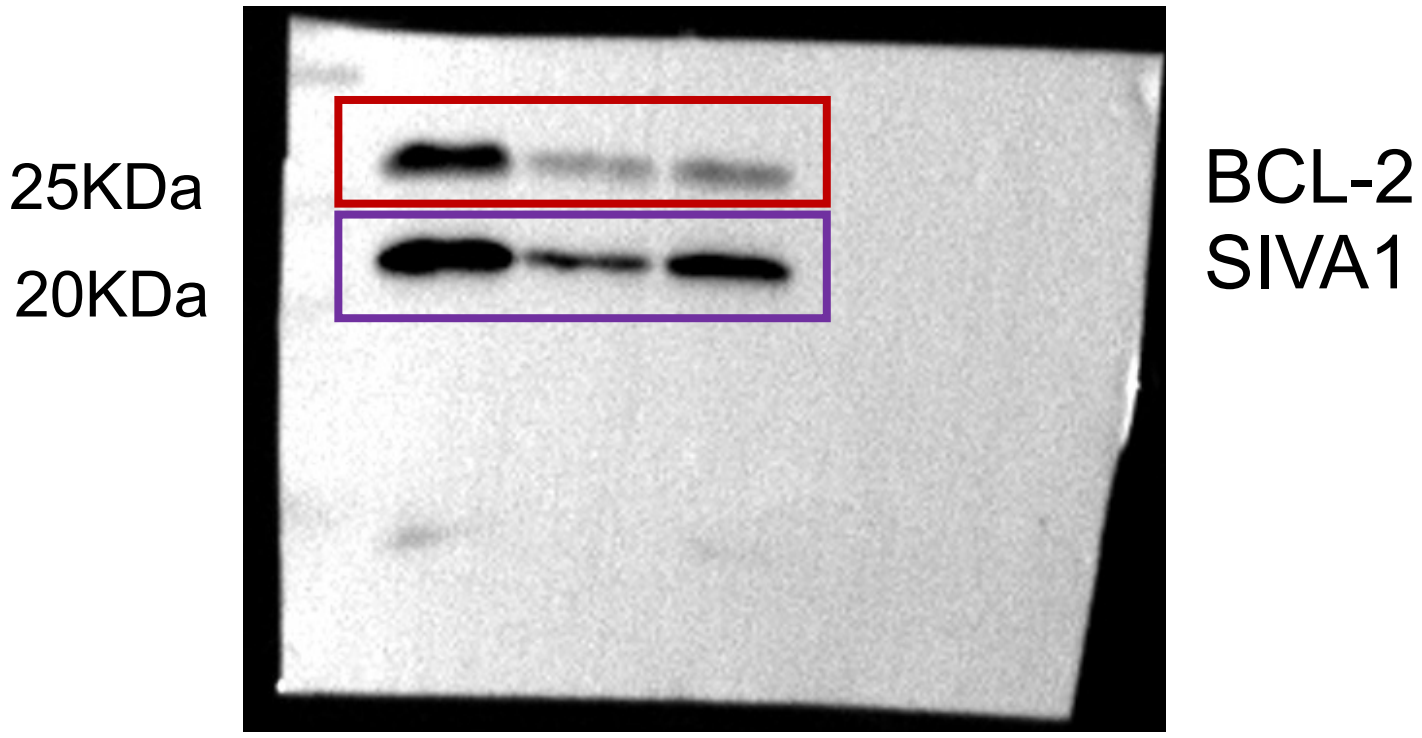

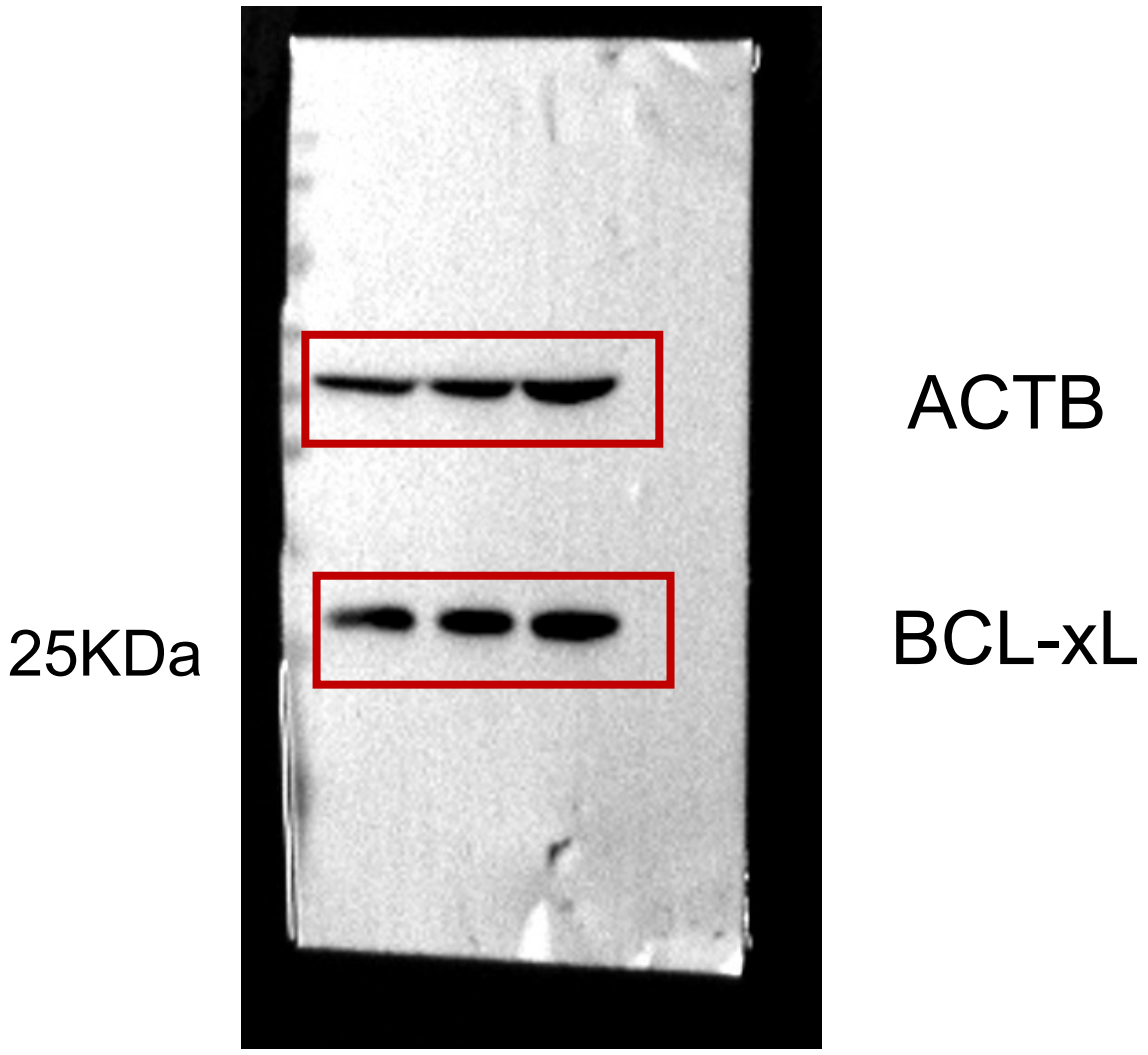

Fig 8F

C2ORF68-OE **NOZ**

ACTB

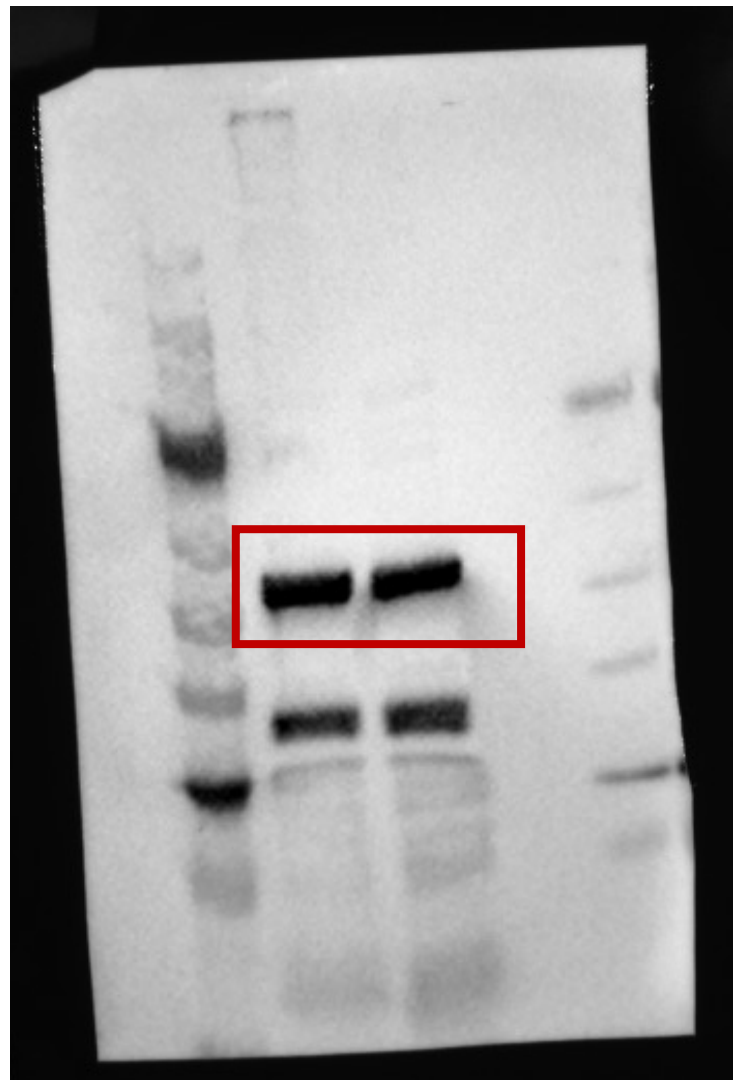

BCL-2

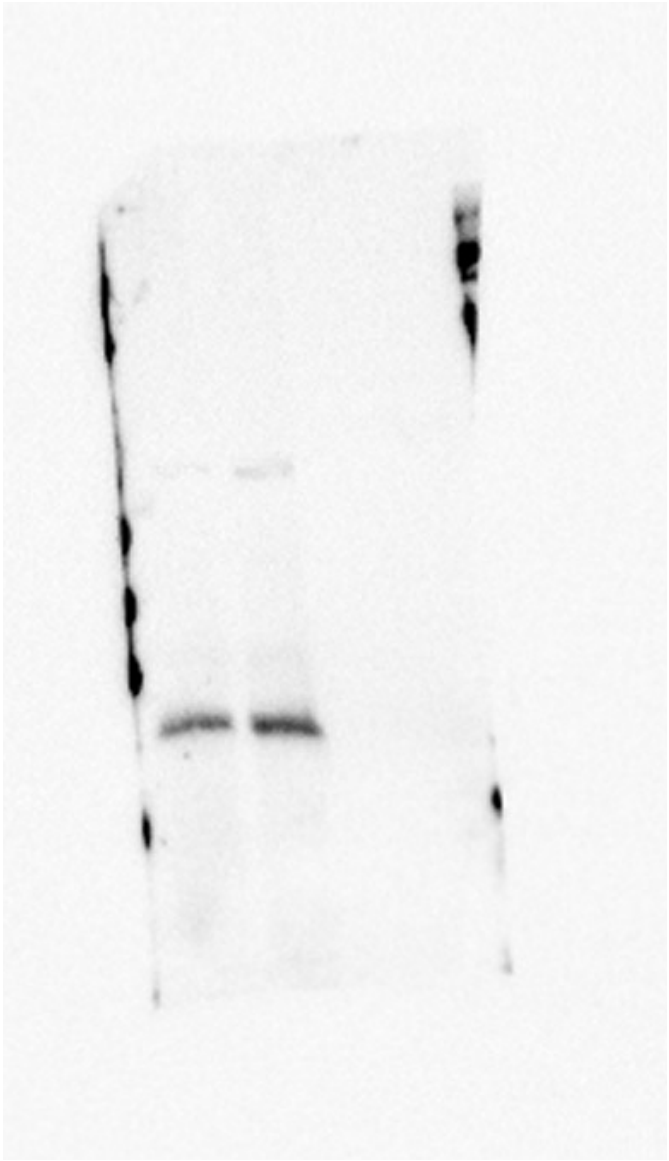

SIVA1

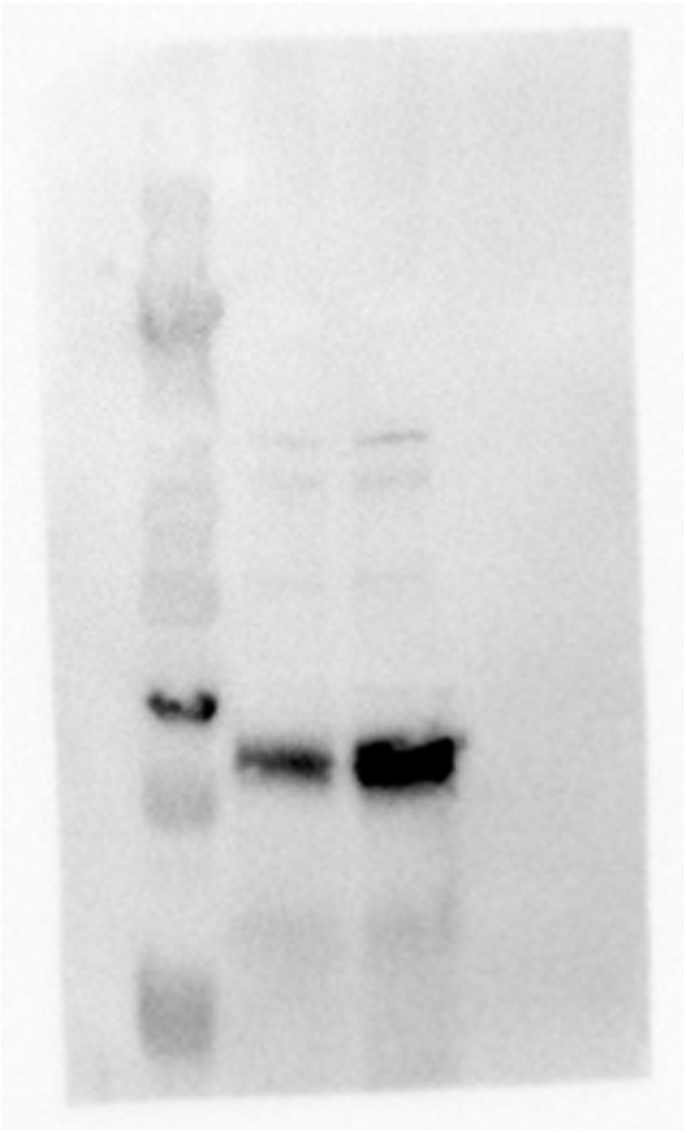

Flag

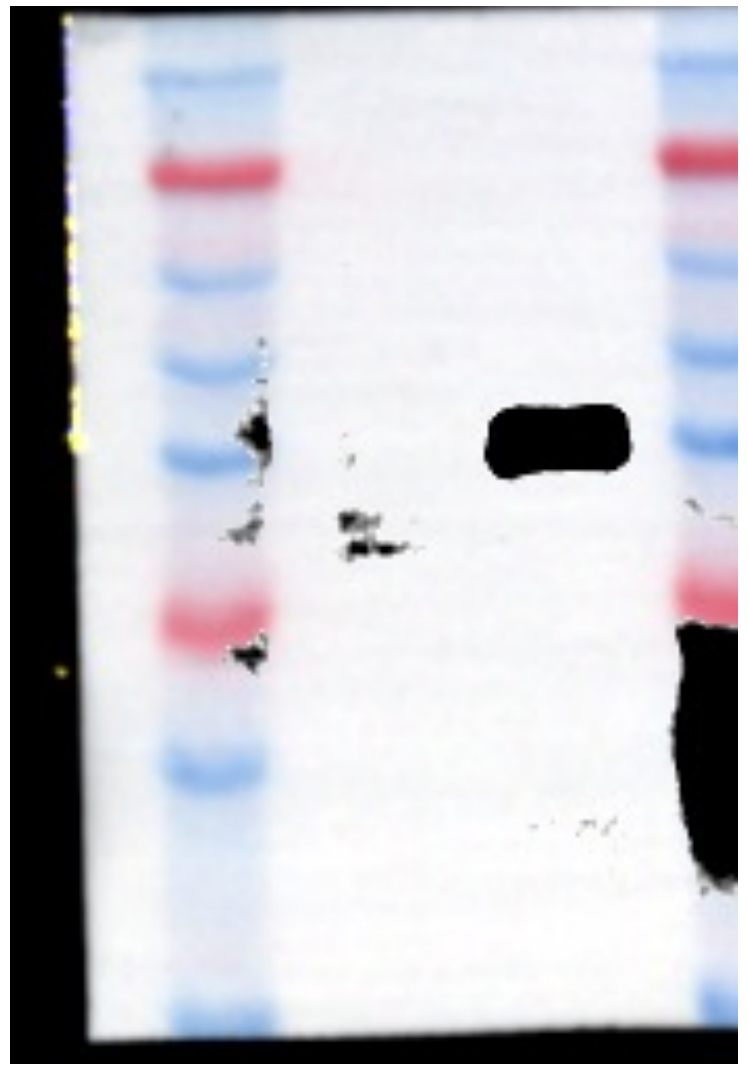

Fig 8F

C2ORF68-OE **GBC-SD**

ACTB

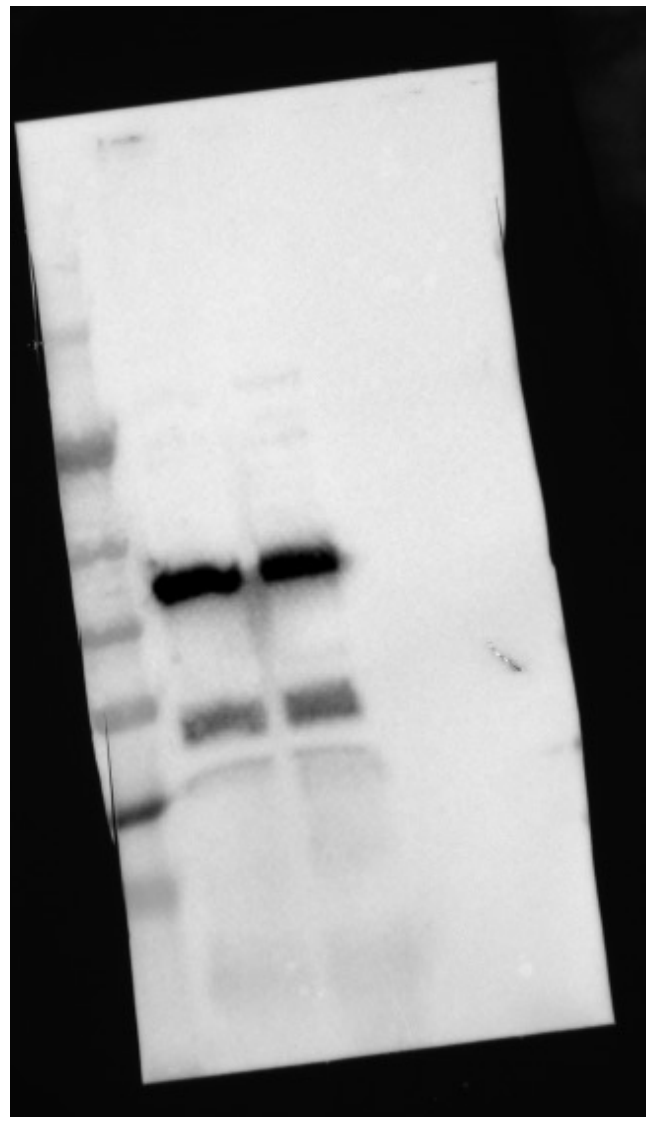

BCL-2

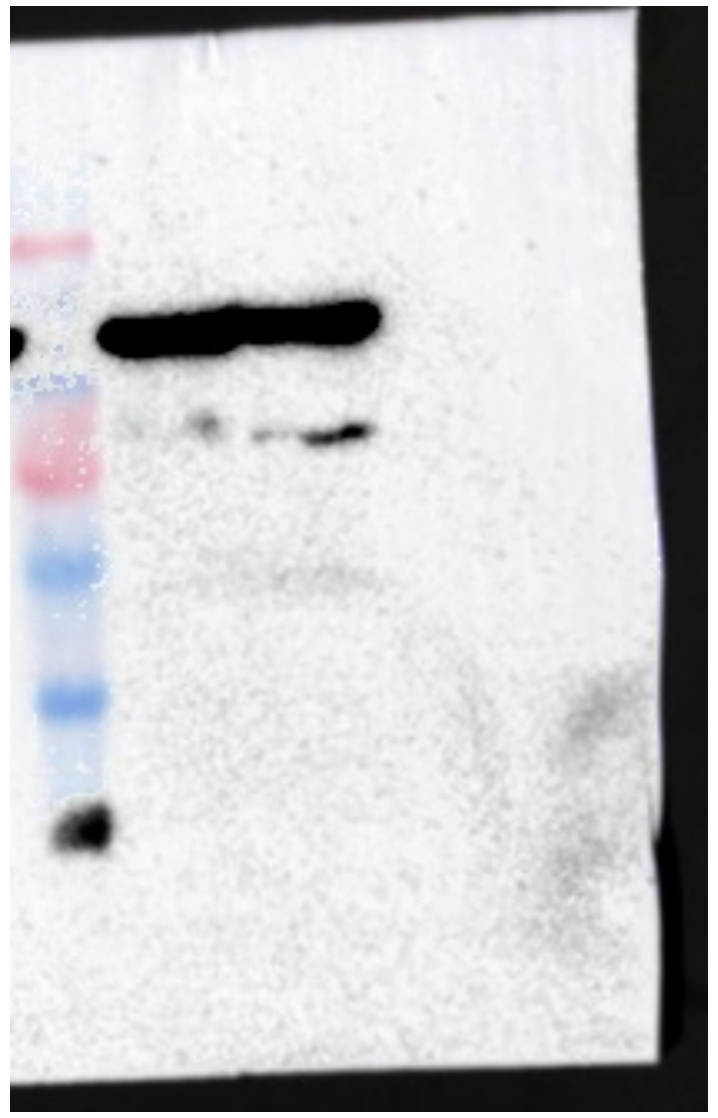

SIVA1

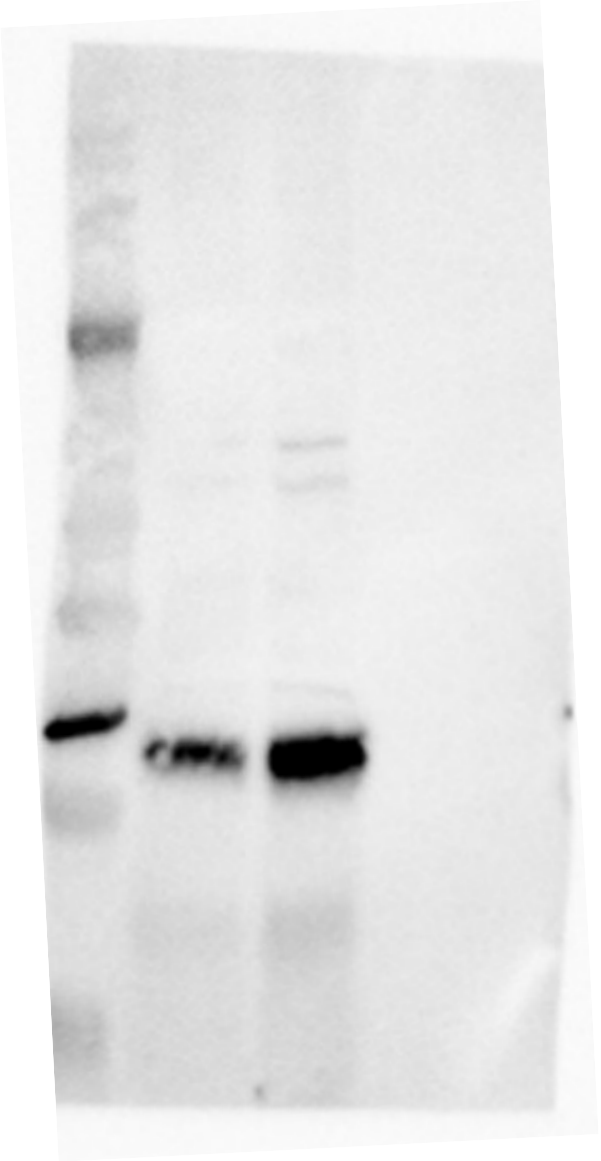

Flag

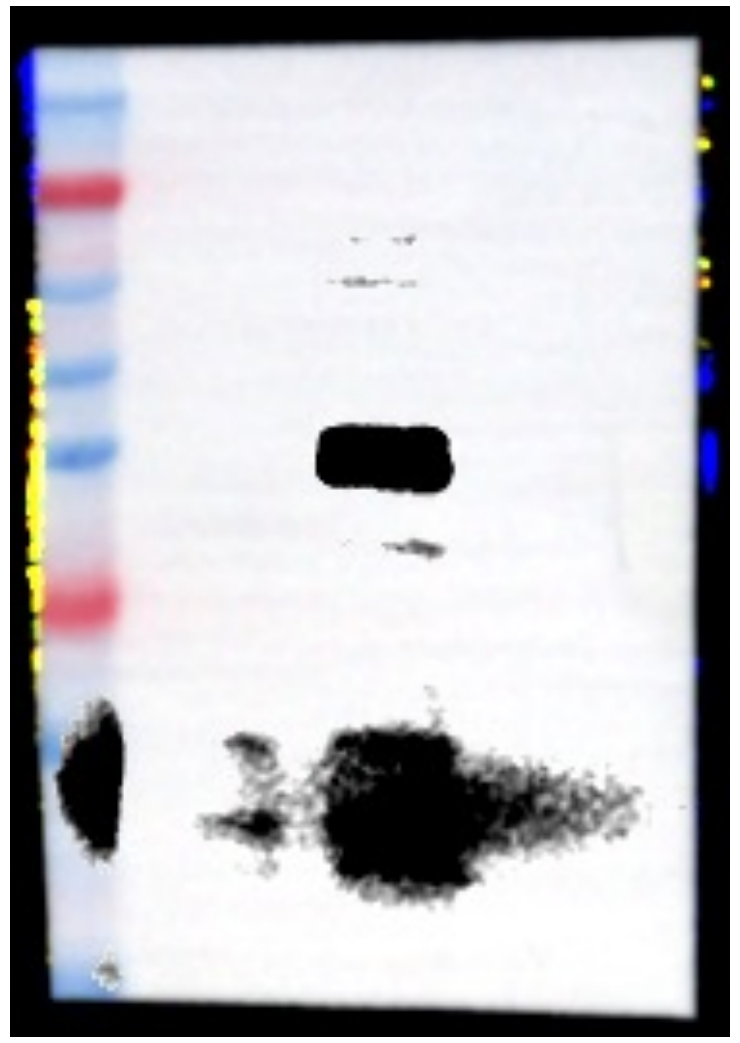

Fig 8H

siC2ORF68 **NOZ**

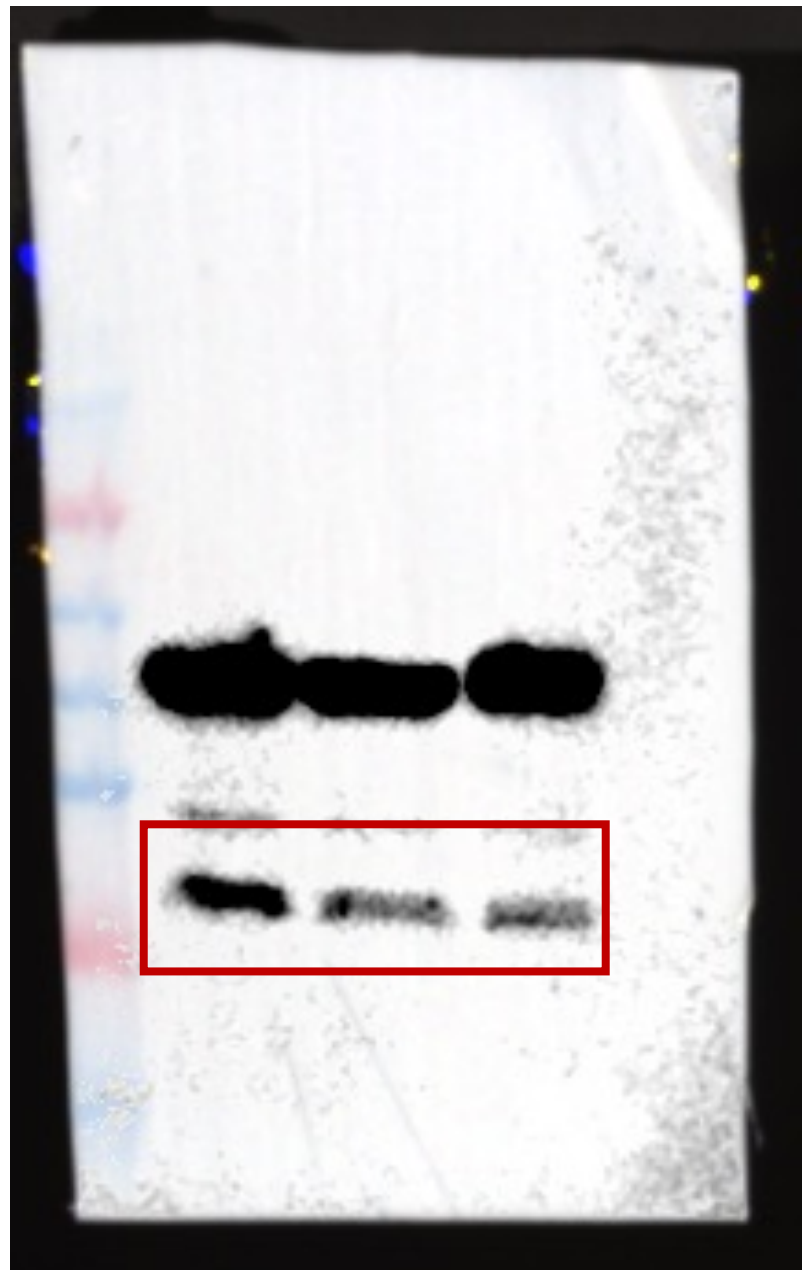

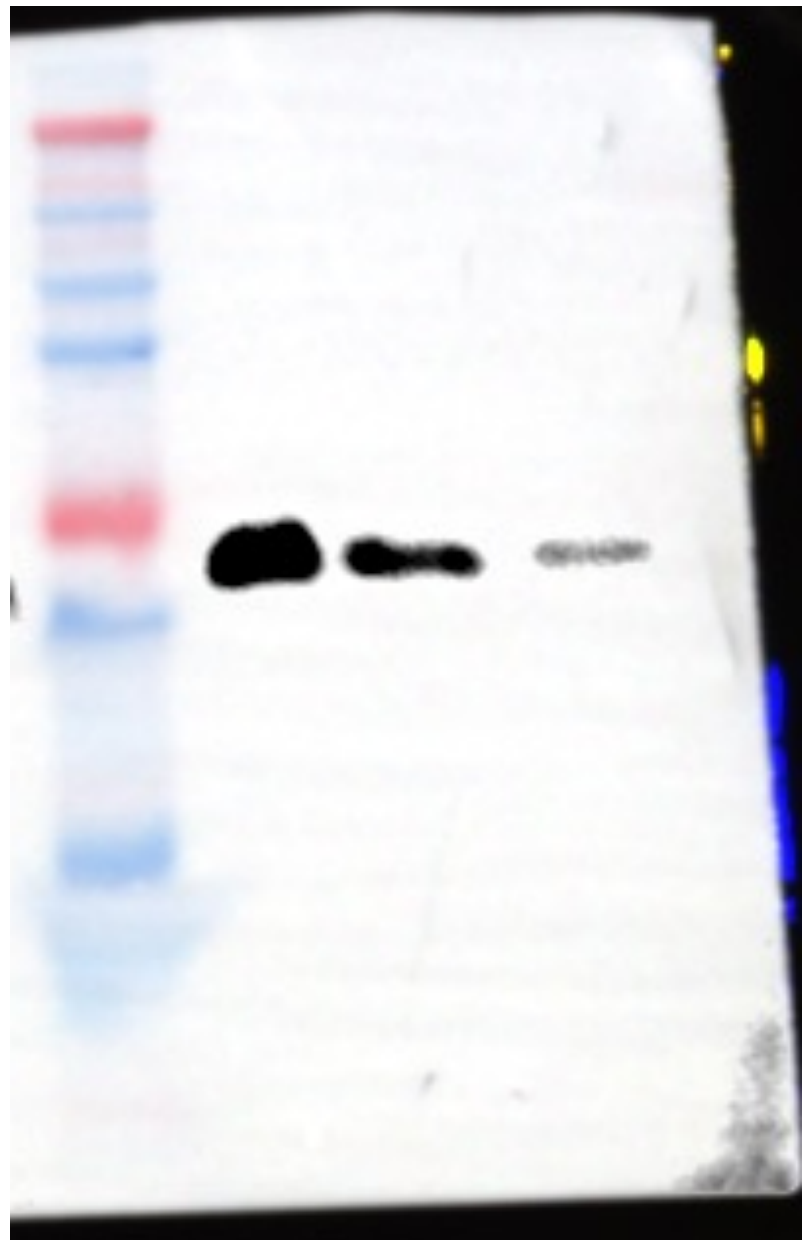

Fig 8H

siC2ORF68 **GBC-SD**

BCL-2

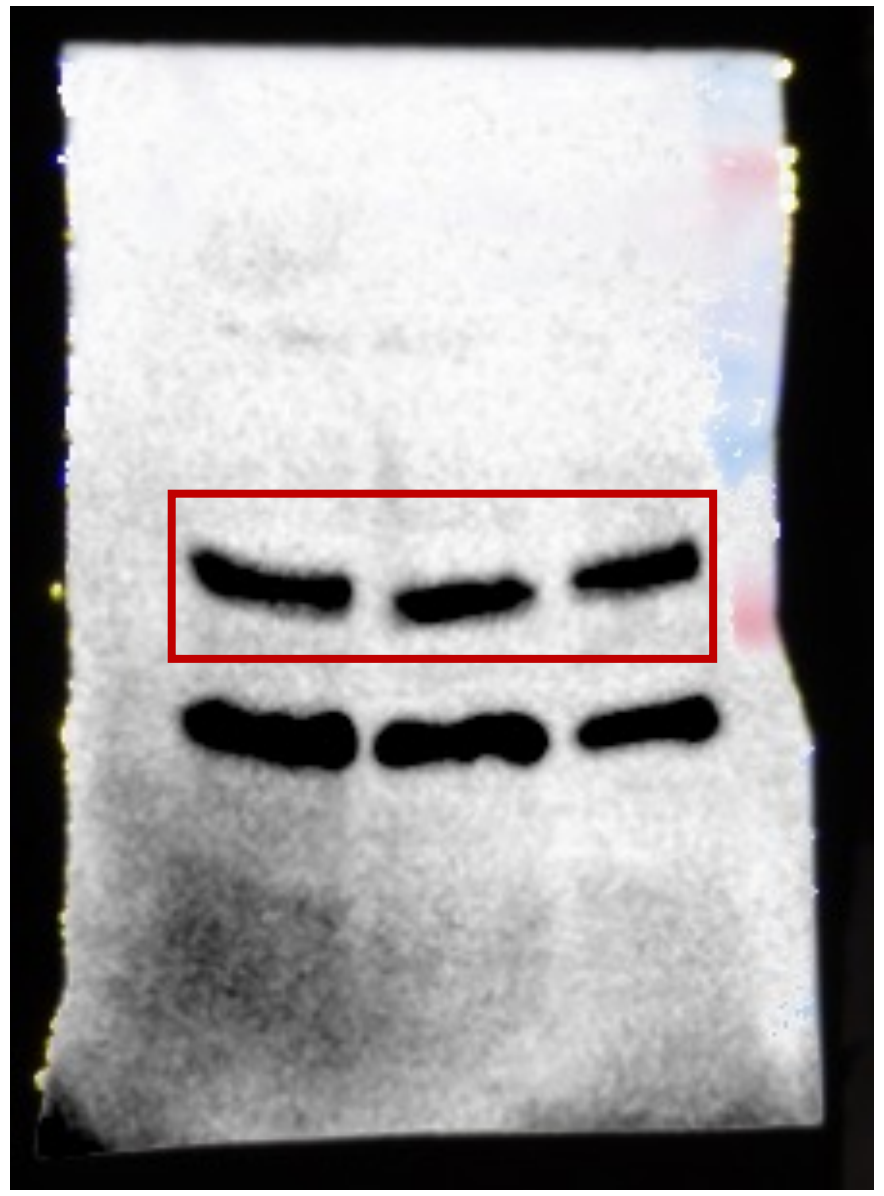

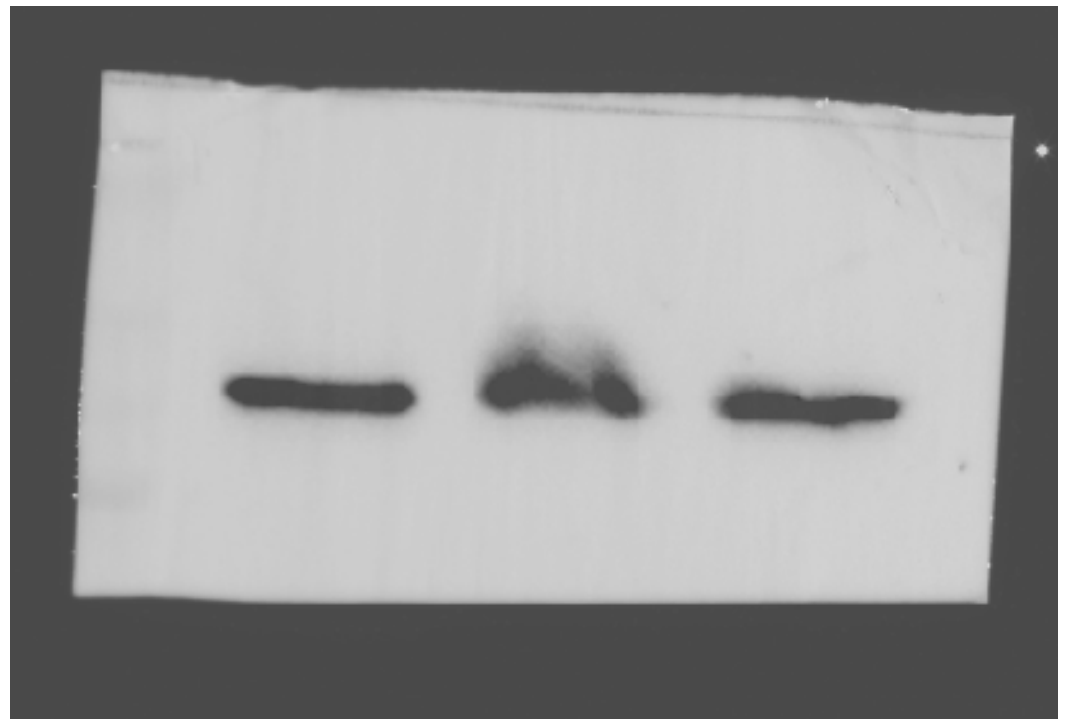

ACTB

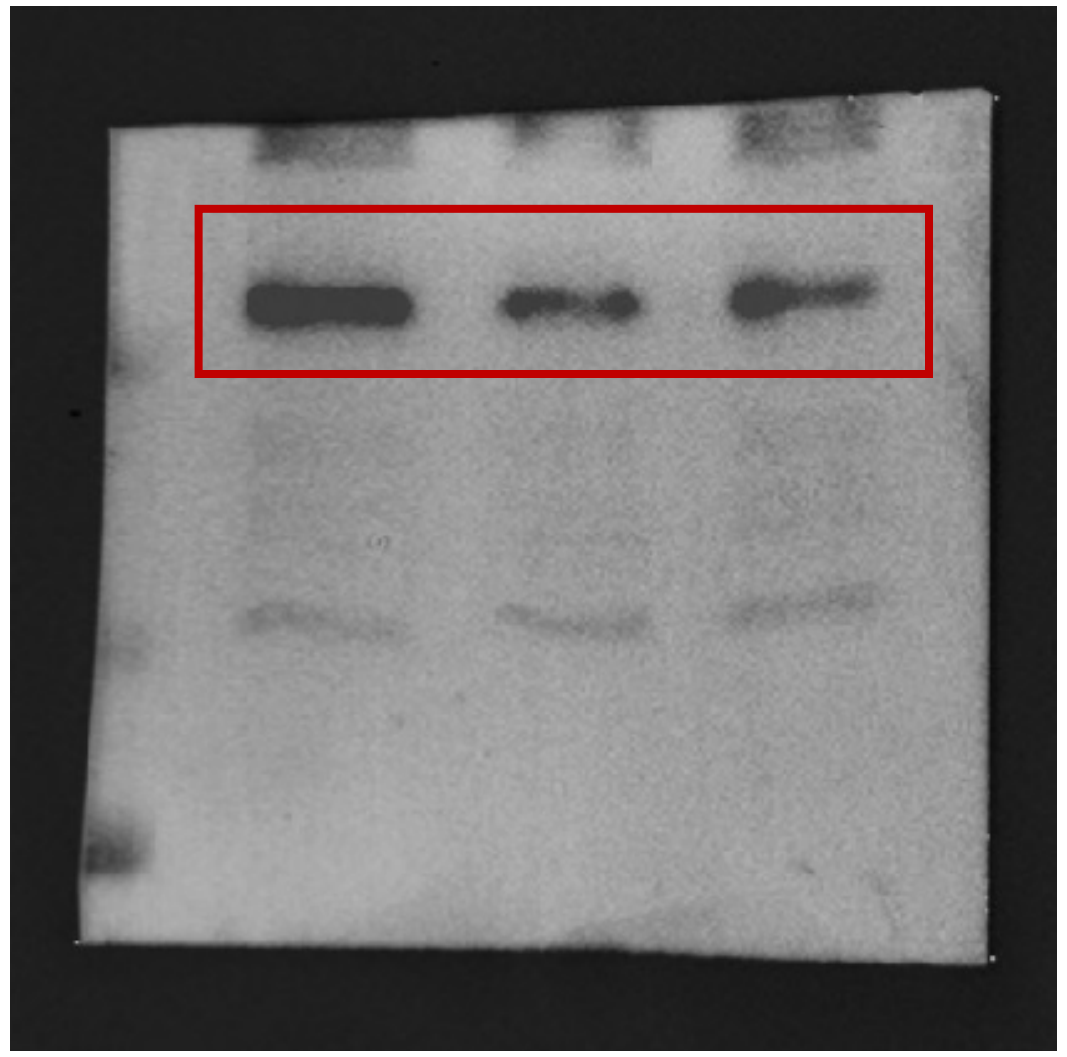

SIVA1
